# Dynamic Changes of Genome-Wide DNA Methylation during Soybean Seed Development

**DOI:** 10.1038/s41598-017-12510-4

**Published:** 2017-09-25

**Authors:** Yong-qiang Charles An, Wolfgang Goettel, Qiang Han, Arthur Bartels, Zongrang Liu, Wenyan Xiao

**Affiliations:** 1 US Department of Agriculture, Agricultural Research Service, Midwest Area, Plant Genetics Research Unit, Donald Danforth Plant Science Center, St. Louis, MO 63132 USA; 2 Department of Biology, Saint Louis University, St. Louis, MO 63103 USA; 3 US Department of Agriculture, Agricultural Research Service, Appalachian Fruit Research Station, Kearneysville, WV 25430 USA

## Abstract

Seed development is programmed by expression of many genes in plants. Seed maturation is an important developmental process to soybean seed quality and yield. DNA methylation is a major epigenetic modification regulating gene expression. However, little is known about the dynamic nature of DNA methylation and its effects on gene expression during plant development. Through whole-genome bisulfite sequencing, we showed that DNA methylation went through dynamic changes during seed maturation. An average of 66% CG, 45% CHG and 9% CHH contexts was methylated in cotyledons. CHH methylation levels in cotyledons changed greatly from 6% at the early stage to 11% at the late stage. Transcribed genes were approximately two-fold more likely to be differentially methylated than non-transcribed genes. We identified 40, 66 and 2136 genes containing differentially methylated regions (DMRs) with negative correlation between their expression and methylation in the CG, CHG and CHH contexts, respectively. The majority of the DMR genes in the CHH context were transcriptionally down-regulated as seeds mature: 99% of them during early maturation were down-regulated, and preferentially associated with DNA replication and cell division. The results provide novel insights into the dynamic nature of DNA methylation and its relationship with gene regulation in seed development.

## Introduction

Soybean is one of the most important seed crops in the world. It serves as a dual-purpose crop, which provides both highly valuable seed protein and oil mainly for animal feeds and human consumption. Soybean seed development goes through two major and distinct developmental processes, embryogenesis and seed maturation, to form mature seeds^1^. Following a rapid cell division and cell differentiation in embryogenesis, the young embryo switches into a distinct seed filling and maturation process in which seed enlarges and synthesizes storage reserve to provide energy resource for seed germination^2^. Seed maturation represents a distinct process in seed development and is important to soybean seed quality and yield. Many regulatory genes such as *LEC1*, *LEC2*, *FUSCA*, and *ABI3* have been found to control seed maturation through transcription in Arabidopsis^3–6^. Seed maturation has been extensively selected through domestication and breeding to develop soybean cultivars with desirable seed quality and yield.

Recently genomic approaches have been employed to study soybean seed development and to identify genes that are unique to a particular seed region at four developmental stages: globular, heart, cotyledon, and early maturation^1,7^. Jones and Vodkin (2013) found more than one hundred genes that are highly expressed exclusively at young seed stages^8^. By using sensitive silicon-substrate photonic crystal protein arrays, Jones *et al*. found that four transcription factors (zinc finger GATA, basic helix-loop-helix, BTF3/NAC [for basic transcription factor of the NAC family], and YABBY) have increased expression during the stages of seedling development^9^. Recently Lu *et al*. reported that 2680 genes were differentially expressed during seed maturation between cultivated and wild soybean accessions by analyzing 40 transcriptomes of developing soybean seeds^10^. They also identified two potential key regulators of seed traits, GA20OX and NFYA, and these two genes showed significantly higher expression in cultivated soybean than wild soybean^10^. Recently, we sequenced soybean seed transcriptomes of nine genotypes at mid-maturation stages, and revealed transcript sequence and expression polymorphisms. Further exploration of the seed transcriptome diversity discovered a set of novel and previously identified DNA variants including splicing mutation, gene expression variation and large DNA deletion responsible for fatty acid composition variation in those soybean genotypes^11,12^.

In plants and animals, 5-methylcytosine is an important epigenetic modification of silent chromatin and is involved in silencing transposable elements (TEs), regulating gene expression, X-chromosome inactivation, genome stability, somaclonal variation, paramutation, imprinting, growth and development^13–19^. Aberrant DNA methylation in promoters is associated with inappropriate gene silencing in animals and plays a critical role in diseases such as cancer^20–22^. DNA methylation in the symmetric CG context is an evolutionarily conserved modification in mammals, plants, and some fungi^23–26^. In mammals, DNA methylation is initiated by *de novo* DNA methyltransferase3 (Dnmt3)^27^ and maintained by maintenance DNA methyltransferase1 (Dnmt1)^28^. In *Arabidopsis*, DNA METHYLTRANSFERASE1 (MET1), an ortholog of Dnmt1 in mammals, is responsible for maintaining CG methylation^29–32^. In addition, *Arabidopsis* has CHROMOMETHYLASE 2 and 3 (CMT2 and CMT3)^33–35^ and the *de novo* DNA methyltransferases DOMAINS REARRANGED METHYLTRANSFERASE 1 and 2 (DRM1 and DRM2)^36,37^ that are responsible for DNA methylation at the CHG and CHH (H = A, C, or T) contexts. Loss of DNA methylation at the CHH context can result in production of small interfering RNA (siRNA) by RNA POLYMERASE IV (Pol IV), which can then recruit *de novo* DNA methyltransferase DRM2 via Pol V and other chromatin remodeling complexes to their target loci to induce *de novo* DNA methylation in TEs and other repetitive sequences^38^. This pathway is called RNA-directed DNA methylation (RdDM)^39–45^. Evidence has shown that DNA methylation plays a critical role in plant growth and development. DNA methylation has been shown to silence TEs, repetitive sequences, transgenes, and genes regulating leaf morphology, flowering time, floral organ identity, fertility, and embryogenesis^46–56^. Mutations in DNA methyltransferase MET1 and DECREASE IN DNA METHYLATION1 (DDM1), an ATP-dependent SWI2/SNF2 chromatin-remodeling factor, also affect seed development^54,57–59^, suggesting that epigenetic marks, such as DNA methylation, play an important role in seed development.

It has been recently reported that there are two putative MET1 homologs (GmMET1 and GmMET2) in soybean^60^. Sequence comparison indicates that GmMET1 and GmMET2 share 96% and 95% sequence identity at nucleotide and amino acid levels, respectively. In addition, the soybean genome encodes four CMT-like (GmCMT1 – GmCMT4), five DRM-like (GmDRM1 – GmDRM5) and two DNMT2-like (GmDNMT2a and GmDNMT2b) DNA methyltransferases^60^. In soybean, there is extensive CG DNA methylation, and differentially methylated regions (DMRs) exist among methylomes of different organs, such as cotyledons, leaves, stems, and roots^61^. DNA methylation in promoters generally inhibits gene expression, and small RNA (sRNA) abundance is reported to positively correlate with hypermethylated regions but negatively relate to hypomethylated regions in soybean^61^. By epigenomic analysis of soybean recombinant inbred lines (RILs) and their parents, Schmitz *et al*. showed that most DMRs cosegregated with the genotype in Mendelian inheritance, whereas some DMRs were found to contain the methylation status of the other parent indicating the uncoupling of genotype and epigenotype^62^. In addition, many methylated Quantitative Trait Loci (methylQTL) were identified which could be a reflection of epigenetic variants^62^. Kim *et al*. recently showed that genes with CG DNA methylation in the gene body are highly expressed and more abundant in duplicated regions retained from the whole-genome duplication event^63^. In addition, diverged methylation patterns in the CHG and CHH contexts were found in TEs and might play a role in regulating gene expression and evolution of genes following polyploidy and speciation^63^. Recently DNA methylation has been shown to affect transposition and splicing of a TE element from a MYB transcription factor regulating anthocyanin synthase genes in soybean seed coats^64^.

Recently, we sequenced small RNAs in soybean cotyledons at six distinct maturation stages. The study provided a comprehensive depiction of miRNAs at gene, pathway and genome levels and further inferred miRNA regulatory network^65^. Despite recent progress in studying the effects of the epigenome on plant growth and development in *Arabidopsis* and crop plants such as soybean, rice, and corn^61–63,66–70^, a comprehensive characterization of methylomes in soybean seeds at different maturation stages is not available. In the study, we sequenced methylomes and transcriptomes of soybean cotyledon at three distinct maturation stages to characterize their dynamic nature during seed maturation. Understanding epigenomic reprogramming during seed development will provide important genetic, epigenetic, and genomic resources and tools for genetic engineering to potentially improve soybean production in the future.

## Results

### DNA methylomes of soybean cotyledons at distinct seed maturation stages

**Figure 1:**
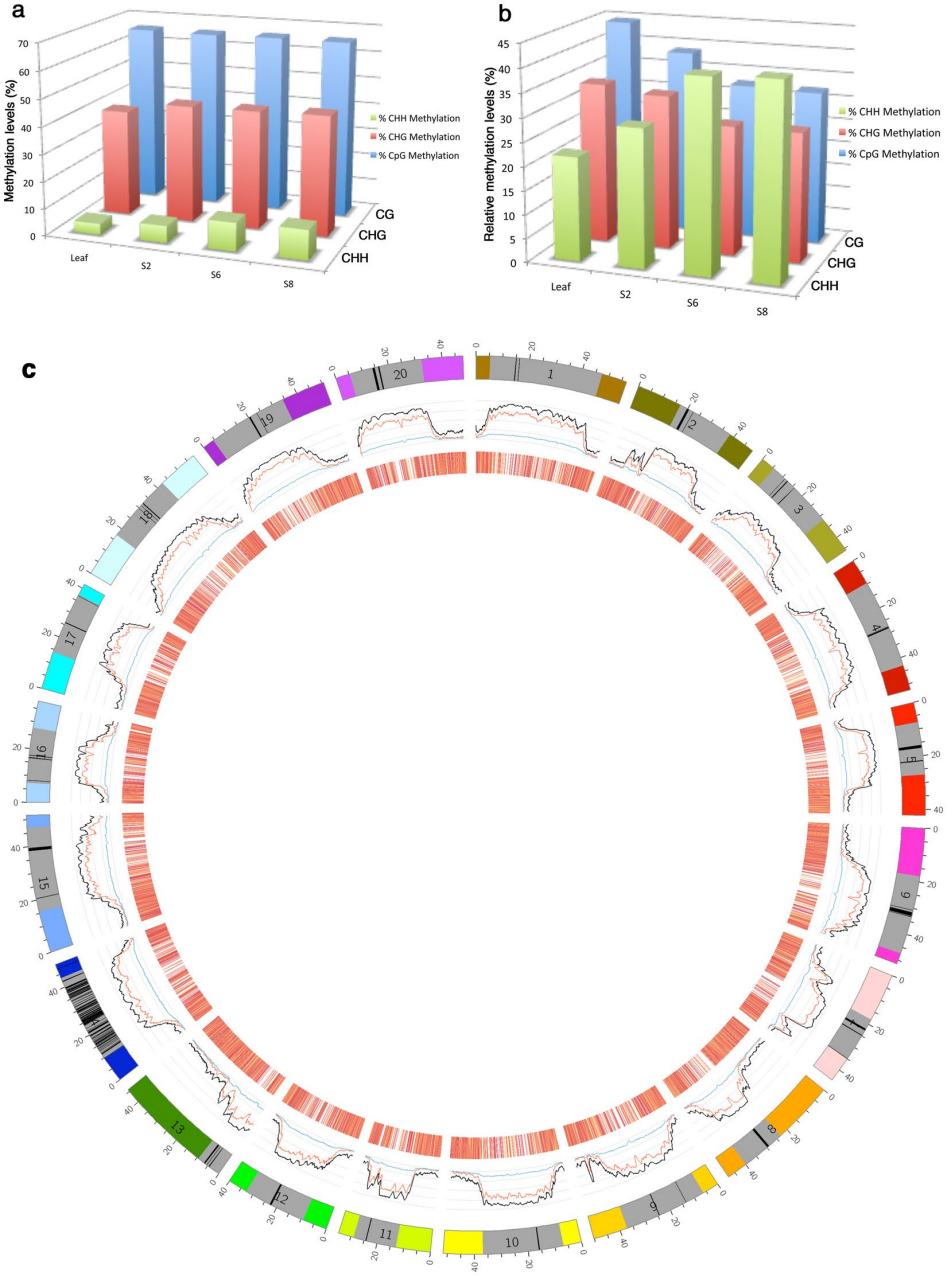
Genome-wide features of DNA methylation and transcriptome of soybean cotyledons. (**a)** The genome-wide percentage of methylated CG, CHG and CHH as a proportion of the total CG, CHG, and CHH, respectively, in leaves and cotyledons at different stages. **(b)** The relative levels of methylated CG, CHG and CHH among the total methylated cytosine in leaves and cotyledons at different stages. **(c)** A circle plot of DNA methylation, and transcriptome in soybean cotyledon at the S6 stage. The outermost circle represents the 20 soybean chromosomes, the numbers 0, 20, 40 outside the circle represent 0 Mb, 20 Mb, and 40 Mb positions on the chromosome, respectively, and solid gray boxes and black bars indicate relative locations of pericentromeric regions and centromeric repeats, respectively. The middle circle shows the percentage of DNA methylation in the CG (black), CHG (red), and CHH (blue) contexts in 1 million base pair (bp) windows that scanned the entire genome with 100,000 bp steps. The innermost circle is a heatmap of gene expression for all expressed genes in the log_2_ FPKM values. A gene with darker bar was expressed at a higher level.

To reveal genome-wide DNA methylation and gain insights into its association with gene expression changes in soybean cotyledons over the course of seed maturation, we sequenced DNA methylomes of cotyledons at the S2, S6, and S8 stages and leaf tissues from soybean cultivar Jack using bisulfite -sequencing (BS-seq) technology (Fig. 1)^71^. S2, S6 and S8 represent early, middle, and late seed maturation stages, respectively^72^. Jack genomic DNA without sodium bisulfite treatment was sequenced to control its sequence variation from soybean reference genome. A total of 720 million 100-bp paired-end reads were generated from sodium bisulfite treated DNA with higher than 99% bisulfite conversion rate for each library. An average of 86 million 100-bp sequencing reads with 8.8 -fold coverage of the soybean genome were aligned to the soybean genome for each sample. On an average, 71.9% of the read pairs were aligned to 89.8% of the soybean genome. Compared to treated DNA, a higher percentage of sequence reads from untreated DNA (91%) aligned to a larger portion of the soybean genome (93%) (Table S1). Two replicates were conducted for S2 and S6 stages to assess biological and experimental reproducibility in DNA methylome analysis. The replications showed R^2^ values of 0.86 at S2 and 0.83 at S6 for all examined DNA methylation regions.

Genome-wide cytosine methylation at CG, CHG and CHH contexts was determined in all examined tissues (Fig. 1a and Table S2). An average of 66% CG and 45% CHG was methylated in cotyledons. In contrast, only 9% CHH was methylated, i.e. a much lower level of methylation. There were no significant genome-wide changes in CG or CHG methylation levels over the course of seed maturation in cotyledons. However, CHH methylation levels changed with statistical significance from 6% at the S2 stage to 10% and 11% at the S6 and S8 stages, representing a 67% and 83% increase compared to the S2 stage, respectively. Leaf as compared to seeds had the lowest levels of DNA methylation at CHG and CHH, with only 4% CHH methylation.

We further determined the composition of all methylated cytosines with respect to CG, CHG, and CHH contexts. Although a very low percentage (4–11%) of CHH was methylated in each examined tissue (Fig. 1a and Table S2), methylated cytosines in the CHH context (^m^CHH) accounted for a relatively large portion (22–41%) of all methylated cytosines (^m^CG + ^m^CHG + ^m^CHH) (Fig. 1b). Approximately 32–44% and 27–34% of methylated cytosines occurred in CG and CHG contexts (Fig. 1b). The relative composition of methylated cytosines in three cytosine contexts varied over the course of seed maturation. Percentage of methylated cytosines in the CHH context increased as seed matured while percentage of methylated cytosines in the CG and CHG contexts remained the same or showed very little changes (Fig. 1a and Table S2), thus relative percentage of ^m^CHH in all the methylated cytosines (^m^CG + ^m^CHG + ^m^CHG) increased over the course of seed maturation while relative percentage of ^m^CG and ^m^CHG decreased (Fig. 1b). Wilcoxon rank sum test indicated that cotyledon at S2 had significant difference from cotyledon at S6 and S8 for all methylation contexts with a P value cut-off of 0.05.

DNA methylation was not evenly distributed on a chromosome in cotyledon or leaf. The chromosomal DNA methylation pattern in cotyledons at the stage S6 represented a typical example (Fig. 1c). Overall, the pericentromeric and centromeric regions, which mostly occupied a large portion in the middle of the chromosomes, were methylated at a higher level than non-pericentromeric regions in each of the three cytosine contexts (Figs 1c and S1 and Table 1). We observed that average methylation levels for CHH, CHG, and CG were 6%, 17%, and 31% in non-pericentromeric regions compared with 13%, 65%, and 85% in centrometeric and pericentrometeric regions, respectively. DNA methylation levels had an opposite trend with gene density. For soybean, density of genes in the pericentromeric regions and centromeric repeat regions (24 genes/Mb) is much lower than that in non-pericentromeric regions (100 genes/Mb). In addition, we observed that the overall chromosomal distribution of transcripts and DNA methylation across each chromosome had opposite patterns (Fig. 1c and Table 1). On an average, density of expressed genes and expression levels of the genes in non-pericentromeric regions were 6-fold and 7-fold as those in heterochromatin regions, respectively. It is likely that both higher number of expressed genes and higher expression levels of a gene in non-pericentromeric regions contributed to its overall higher transcript accumulation levels than in centromeric and pericentromeric regions (Fig. 1c and Table 1).

**Table 1:** Distribution of genes in non-pericentromeric and pericentromeric and centromeric regions on chromosomes and average gene expression and DNA methylation levels.

	Pericentromeric and centromeric regions	Non-pericentromeric regions
Transcript accumulation (FPKM)	17	119
CHH methylation (%)	13	6
CHG methylation (%)	65	17
CG methylation (%)	85	31
No. of genes/Mb	24	100
No. of expressed genes/Mb	11	65

### Distinct patterns of DNA methylation in genes and transposons in soybean cotyledons

**Figure 2:**
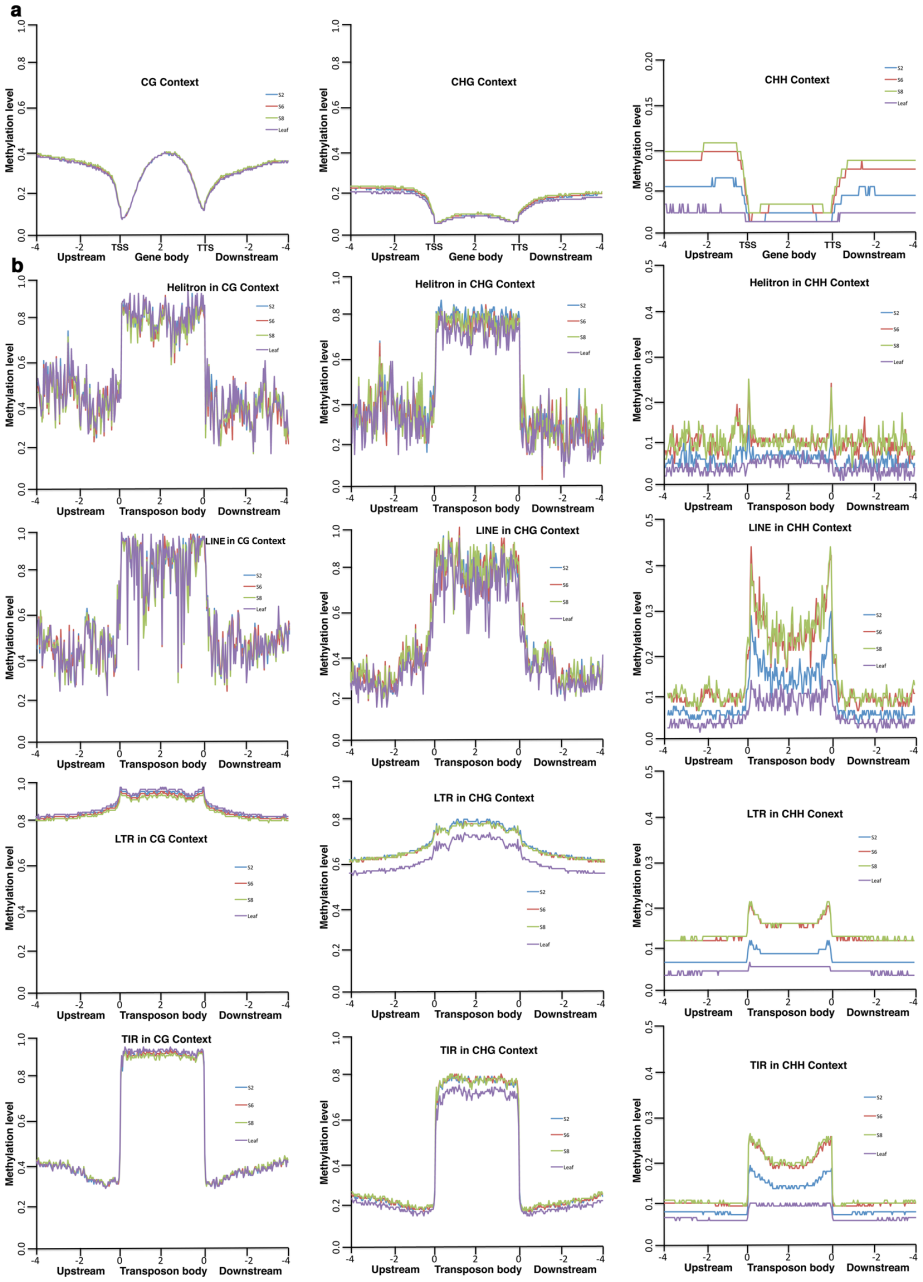
DNA methylation patterns in protein-coding genes and transposons in soybean cotyledons. (**a)** End analysis of ^m^CG, ^m^CHG, and ^m^CHH levels for each bin in gene bodies and in 4 kb upstream of the transcription start site (TSS) and 4 kb downstream of the transcription termination site (TTS) in leaf and cotyledon at S2, S6, and S8. **(b)** End analysis of ^m^CG, ^m^CHG, and ^m^CHH levels in leaf and cotyledon in the following transposons and their 4 kb upstream and downstream flanking regions: Helitron DNA transposons, Long Interspersed Nuclear Element (LINE) retrotransposons, Long Terminal Repeat (LTR) retrotransposons, and Terminal Inverted Repeat (TIR) DNA transposons. The upstream, gene body and downstream regions were divided into 100 bins respectively. Percentage of methylation in each bin is shown on Y-axis.

We determined patterns of DNA methylation in 4 kb upstream of the transcription start site (TSS), gene body, and 4 kb downstream of the transcription termination site (TTS)^73,74^. About 30–40% of CG was methylated at both gene bodies and their flanking regions with a dramatic drop near TSS and TTS (Fig. 2a). CHG and CHH methylation patterns were similar with higher methylation levels at 5′ and 3′ flanking regions, but lower levels in gene bodies (Fig. 2a). In general, there was no obvious change in CG or CHG methylation patterns or methylation levels in cotyledons at S2, S6, and S8. However, CHH methylation levels in the 5′ and 3′ flanking regions showed the biggest changes at the three developmental stages with the highest coefficient variation (Table S3). Although overall CHH methylation levels were much lower, CHH methylation levels in both 5′ and 3′ flanking regions increased over the course of seed maturation. CHH methylation levels in 5′ and 3′ flanking regions increased from 5% at the S2 stage to approximately 9–10% at the S6 and S8 stages. Interestingly, leaf tissues had the lowest CHH methylation levels (2.5%) in 5′ and 3′ flanking regions, only approximately half of that in seeds at S2, and a quarter of that in seeds at S6 and S8 (Fig. 2a).

To understand DNA methylation in transposable elements (TEs) and flanking regions during seed maturation, we measured cytosine methylation in DNA transposons (Helitrons and Terminal Inverted Repeats (TIR) transposons) and retrotransposons (Long Interspersed Nuclear Elements (LINE) and Long Terminal Repeat (LTR) retrotransposons). The TE information used here was from SoyTEdb^75^. Overall, CG and CHG methylation levels in TEs were much higher than those in protein coding genes. TEs often had more than 80% CG and CHG methylation (Fig. 2b), indicating that transposons were preferentially methylated in the soybean genome. Helitron, LINE and TIR, had very high CG and CHG methylation levels (>80%) with a reduction by about half in the 5′ and 3′ flanking regions (Fig. 2b). LTR transposons, which are mainly located in the heterochromatic pericentromeres, had extremely high CG and CHG methylation in TEs with less dramatic reduction in 5′ and 3′ flanking sequences (Fig. 2b).

Little or no obvious variation for CG and CHG methylation levels was observed in cotyledons among different maturation stages, or for CG between cotyledons and leaves. When we calculated coefficient of variation (CV) among S2, S6, and S8 stages for CG, CHG, and CHH contexts, CV for genes at CHH (29.6%) is approximately 20- and 9-times as CV at CG (1.5%) and CHG (3.4%), respectively (Table S3). A similar trend was also observed for TEs (Helitron, LINE, LTR, and TIR, Table S3). Thus, overall, CHH methylation levels had the biggest variation at different maturation stages. CHH methylation in TEs increased over the course of seed maturation. Leaf had the lowest levels of CHH methylation in TEs (Fig. 2b).

### Differentially methylated regions (DMRs) occurred more frequently in transcribed genes than non-transcribed genes

We compared DNA methylation levels in 4-kb-long 5′ flanking region of protein coding genes in cotyledons among S2, S6 and S8 stages to identify differentially methylated regions (DMRs) whose methylation levels varied by more than 30% between two compared stages. As shown in Table 2, we identified a total of 29,487, 407, and 177 DMRs in CHH, CHG, and CG contexts (CHH-DMRs, CHG-DMRs, and CG-DMRs) during early maturation (S2 to S6), respectively. Those CHH-, CHG-, and CG-DMRs were located in 13,324, 367 and 162 genes, respectively. We also identified 27,520 CHH-DMRs, 795 CHG-DMRs, and 444 CG-DMRs in 14,739, 700 and 408 genes, respectively, during late maturation (S6 to S8) (Table 2). The vast majority of DMRs (97%) were in the CHH context while only approximately 3% DMRs were in CG and CHG. For example, we identified a total of 13,324 CHH-DMR genes, only 367 CHG-DMR and 162 CG-DMR genes during early maturation (Table 2). Majority of 13,324 CHH-DMR genes (8,732) were differentially methylated during early, late and entire maturation processes (Fig. S2).

**Table 2:** Preferential presence of DMRs in expressed and non-expressed genes.

	S2 to S6			S6 to S8		
	CHH	CHG	CG	CHH	CHG	CG
Total number of DMRs	29,487	407	177	27,520	795	444
Number of DMR genes	13,324	367	162	14,739	700	408
Number of expressed DMR genes	11,430	299	138	12,489	533	332
Number of non-expressed DMR genes	1,894	68	24	2,250	167	76
Number of expressed DMR genes/Total number of expressed genes (%)	27.52%	0.72%	0.33%	30.06%	1.28%	0.80%
Number of non-expressed DMR genes/Total number of non-expressed genes	13.06%	0.47%	0.17%	15.51%	1.15%	0.52%

Total number of expressed and non-expressed genes were 41,540 and 14504, respectively.

We observed that transcribed genes were more likely differentially methylated than un-transcribed genes. Our result showed that 41,540 genes were transcribed in the examined tissues while 14,504 genes were not (Table 2). The percentage of transcribed genes that contained DMRs was about 2-fold as that of un- transcribed genes. For instance, approximately 28% and 30% of transcribed genes had CHH-DMRs from S2 to S6 and from S6 to S8, respectively, while approximately only 13% and 16% of un-transcribed genes had CHH-DMRs (Table 2).

### Genes potentially regulated by dynamic DNA methylation during seed maturation

**Figure 3:**
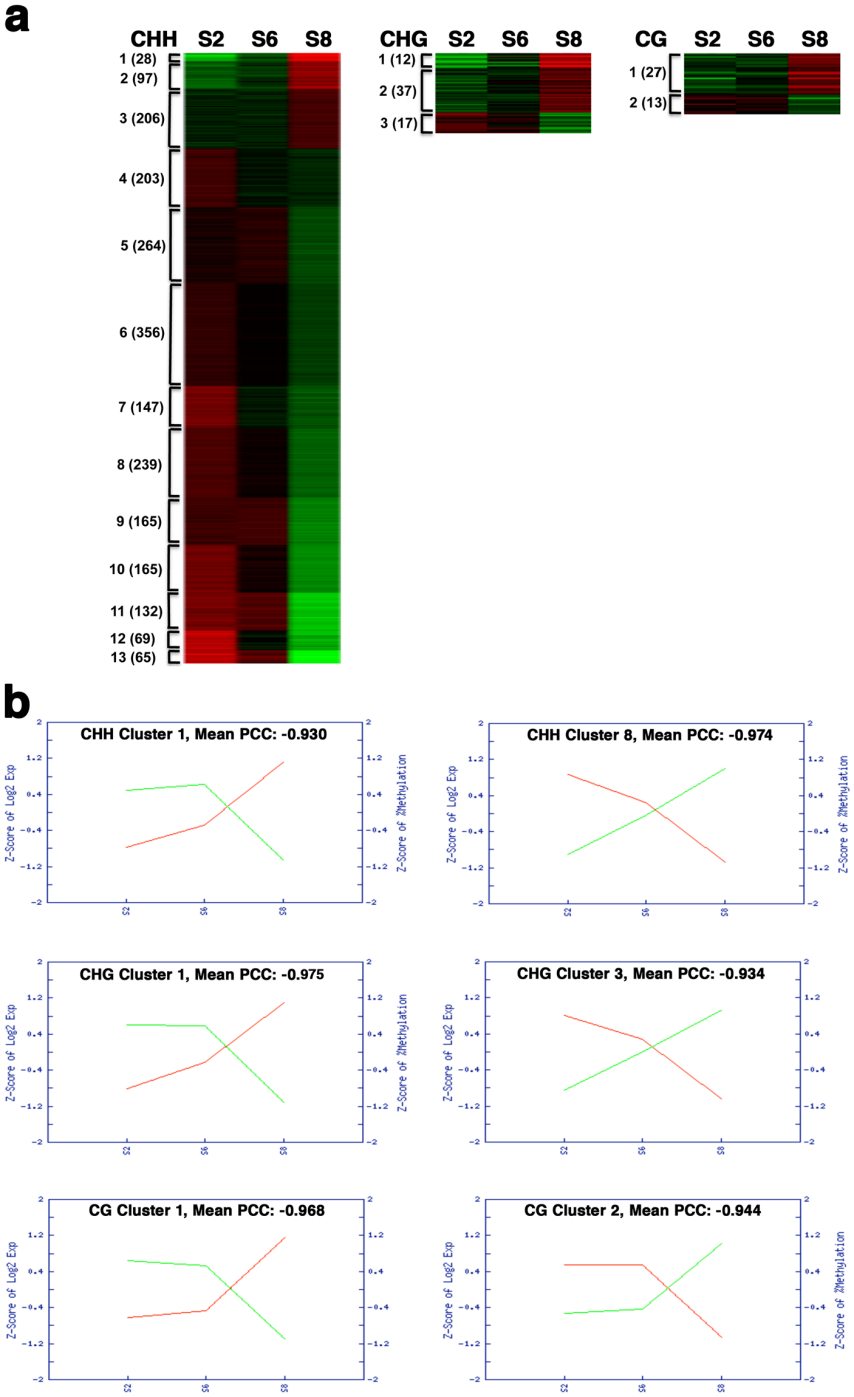
Distinct expression patterns of genes that were potentially regulated by DNA methylation during seed maturation. (**a)** Expression patterns of gene clusters based on gene transcription patterns in cotyledon at three stages and DNA methylation in DMRs in ^m^CHH, ^m^CHG and ^m^CG contexts. The green to red color gradient represents low to high gene expression, respectively. Genes with 1) more than 30% DNA methylation changes among three different seed stages S2, S6, and S8; 2) statistically significant changes in gene expression; and 3) negative correlation (PCC < −0.85) between gene expression and methylation levels, were used for a cluster analysis. **(b)** Relationship between gene transcription and DNA methylation in clusters. For each specific cluster, the Z-score of log_2_ expression (Red) and the Z-score of DNA methylation levels (Green) were shown at each stage. The mean PCC between Z-score of log_2_ expression and Z-score of DNA methylation levels was also calculated and shown. The complete data set is shown in Figure S3.

We identified 40 CG-DMR, 66 CHG-DMR, and 2136 CHH-DMR genes with negative correlation between their expression and DNA methylation at three maturation stages. Those DMR genes were clustered into 2, 3, and 13 distinct expression patterns for CG, CHG, and CHH contexts, respectively, based on their transcript accumulation levels (Fig. 3a and Table S4). In each of these clusters, the mean PCC between Z-scores of log_2_ expression and Z-scores of DNA methylation is smaller than – 0.9 (Figs 3b and S3). Genes in each cluster had a similar expression pattern during seed maturation, and their overall expression levels at three maturation stages were inversely correlated with DNA methylation levels. For example, expression of 28 genes in the CHH-DMR cluster 1 was the lowest at the S2 stage, increased slightly at S6, and reached the highest level at S8, and their methylation levels were opposite: higher at S2 and S6, and lower at S8 (Fig. 3b). In contrast, expression of 239 genes in the CHH cluster 8 was the highest at S2, decreased at S6, and was further reduced to the lowest level at S8, and their methylation levels were exactly opposite: lowest at S2, increased at S6, and reached the highest at S8 (Fig. 3b). For the CHG-DMR cluster 1 and 3, and the CG-DMR cluster 1 and 2, their expression and DNA methylation profiles were similar to those in the CHH cluster 1 and 8, respectively (Fig. 3b). Despite that those genes had overall negative correlation between their expression and methylation at three maturation points, several clusters such as CHH cluster 1 had strong negative correlation during one of three maturation processes, but weak or less obvious positive correlation in another maturation process. It implies that methylation potentially regulates expression of genes during one, but not all of three maturation processes.

A total of 6,187 and 14,778 genes were differentially expressed during early and late maturation processes respectively (Table 3). Number of genes with increased expression (7361) was similar to that with decreased expression (7417) during late maturation, but approximately 2.5-fold genes (4414 versus 1773) had decreased as increased expression during early maturation. Among the genes with negative correlation between their expression and methylation, more genes occurred from S6 to S8 than from S2 to S6 (982 versus 559 genes, or 64% versus 36%, Table 3). Interestingly, there were much more DMR genes with decreased rather than increased expression at each process of seed maturation: early maturation from S2 to S6 (552 versus 7 genes, or 98.7% versus 1.3%), and late maturation from S6 to S8 (733 versus 249, or 74.6% versus 25.4%).

**Table 3:** Differentially expressed genes regulated by differential CHH DNA methylation in seed.

		S2 to S6	S6 to S8
Differentially expressed genes	Increased expression	1773	7361
	Decreased expression	4414	7417
	Total	6187	14778
Differentially expressed genes regulated by differential DNA methylation	Increased expression	7	249
	Decreased expression	552	733
	Total	559	982

We conducted a Gene Ontology (GO) enrichment analysis for the 552 DMR genes (98.7% of the total CHH DMR genes) showing more than 2-fold decreased expression and higher than 30% increased DNA methylation during early seed maturation (Table 4). The enrichment analysis revealed that genes involved in cell division and DNA replication related GO terms such as cytokinesis, cell proliferation, DNA replication, cell cycle regulation, microtubule cytoskeleton organization and motor activity, and spindle assembly were significantly over-represented, suggesting that activities of the genes related to cell division and growth are likely suppressed by DNA methylation during early seed maturation. This is consistent with cellular activity switch from embryogenesis with rapid cell division and differentiation to seed maturation with active cell expansion and production of seed storage reserve, but little cell dividing activity. Interestingly, we also observed that the down-regulated genes were overrepresented in gene silencing and DNA methylation pathways. This result was obtained from the whole genome approach and it requires support from future experimental evidence.

**Table 4:** Over-represented Gene Ontology (GO) of 552 differentially regulated CHH-DMR genes with decreased expression during early seed maturation*.

GO ID	Genome GO Count	Observed no of genes	Expected no of genes	Represen-tation	Corrected Probability	GO description
GO:0000911	471	33	8.652901474	Overrepresented	4.20E-08	cytokinesis by cell plate formation
GO:0008283	388	27	7.128080195	Overrepresented	2.49E-06	cell proliferation
GO:0051567	443	29	8.138503934	Overrepresented	2.69E-06	histone H3-K9 methylation
GO:0016458	156	17	2.865929151	Overrepresented	2.72E-06	gene silencing
GO:0006275	255	20	4.684691881	Overrepresented	3.53E-05	regulation of DNA replication
GO:0006270	159	15	2.921043173	Overrepresented	0.000146972	DNA replication initiation
GO:0051726	345	22	6.338112544	Overrepresented	0.000290873	regulation of cell cycle
GO:0003777	184	13	2.453055891	Overrepresented	0.000321363	microtubule motor activity
GO:0006260	270	19	4.960261991	Overrepresented	0.000395838	DNA replication
GO:0034968	258	18	4.739805903	Overrepresented	0.000872093	histone lysine methylation
GO:0006306	421	23	7.734334438	Overrepresented	0.002243621	DNA methylation
GO:0010389	155	13	2.84755781	Overrepresented	0.003397437	regulation of G2/M transition of mitotic cell cycle
GO:0000226	379	21	6.962738129	Overrepresented	0.004694561	microtubule cytoskeleton organization
GO:0051225	98	10	1.800391389	Overrepresented	0.007293078	spindle assembly

*Those 552 genes have a negative correlation between their DNA methylation and gene expression levels during early seed maturation (P value < 0.01).

We also did a GO analysis of the 733 CHH DMR genes showing more than 2-fold decreased expression and higher than 30% increased DNA methylation during late seed maturation (Table S5). We observed that those genes were less biased with respect to GO terms. They were over-represented in only two GO terms with statistical significance: pattern specification and anthocyanin accumulation in response to UV light (Table S5).

### Seed-specific expressed genes that were differentially methylated

**Figure 4:**
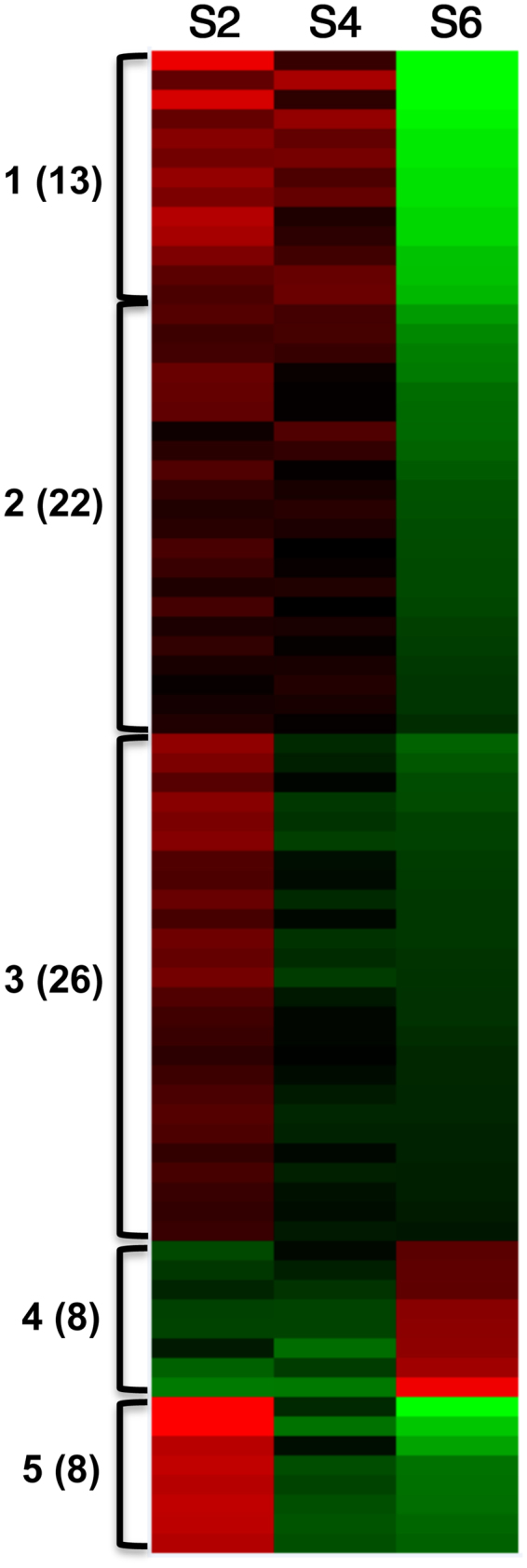
Cluster analysis of 77 genes with seed-specific CHH DMRs based on gene expression at stages S2, S6, and S8. Gene clusters based on gene transcription patterns in cotyledon at three stages and DNA methylation in DMRs in ^m^CHH, ^m^CHG and ^m^CG contexts. The green to red color gradient represents low to high gene expression, respectively. Genes with 1) more than 30% DNA methylation changes among three different seed stages S2, S6, and S8, 2). statistically significant changes in gene expression and 3). a negative correlation (PCC < −0.85) between gene expression and methylation level were used for cluster analysis.

We identified 640 DMR genes expressed in seeds, but not in leaf. Eighty-one (77 in CHH, 2 in CHG, and 2 CG) out of the above 640 DMR genes had a significant negative correlation between their DNA methylation and gene expression. The 77 CHH-DMR genes can be clustered into 5 groups based on their gene expression patterns at three maturation stages (Fig. 4 and Table S6). Seventy-seven CHH-DMR genes were preferentially involved in cotyledon vascular tissue pattern formation, organ senescence, nutrient detection, promoting transcription factor binding, nuclear ubiquitin ligase complex, skotomorphogenesis, 2-isopropylmalate synthase activity, reproductive development, leucine biosynthesis, and transferase activity. This result indicates that these seed-specific genes are likely regulated by DNA methylation during seed maturation directly or indirectly.

## Discussion

In plants significant progress has been made in the epigenetic field. Recently DNA methylomes have been sequenced in many plant species including *Arabidopsis*, rice, maize, soybean, tomato, and wild cabbage^61,66–68,70,76,77^. Studies of DNA methylome reveal many genome-wide DNA methylation features in soybean^61–63,78^.

Levels of DNA methylation vary in different tissues and organs^61,79–82^, and DNA methylation plays a pivotal role in plant development^48,57^. Although the genome undergoes dynamic genome-wide demethylation and methylation processes during gametogenesis and embryogenesis in mammals^83,84^, DNA methylation was thought to be a relatively stable modification, so it is not clear whether a gene’s methylation profile would undergo dynamic changes during plant development. During the transition from early embryogenesis to seed maturation, seeds undergo a dramatic change of metabolic and cellular activities from active cell divisions and differentiation to synthesis of seed storage reserve. It remains unknown whether the epigenetic status also undergoes dynamic change, and affects gene expression, thus potentially altering metabolism during seed maturation. Different from mammals, plants have DNA methylation in CHG and CHH in addition to the CG context. In this research, we show there are no obvious changes of overall DNA methylation levels at the genomic level in the CG and CHG context, but a small number of DMRs in CG and CHG exist during seed maturation (Figs 1a and 3a, and Table 2). However, there is a significant change of CHH DNA methylation in cotyledons among different stages of seed development, S2, S6, and S8, and overall CHH DNA methylation levels increase as seed matures from S2 to S8 (Fig. 1a). A total of 40 CG-, 66 CHG-, and 2136 CHH-DMRs have been identified, suggesting that they potentially regulate differential gene expression during seed development in soybean. Fewer CG- and CHG-DMRs suggest that they are more stable or less dynamic over the course of seed maturation. CHH-DMRs accounted for most DMRs, implying that CHH methylation is likely involved in regulating seed maturation. A recent study using the cotton fiber, the epidermal hair on the cotton ovule, as a model, shows that DNA methylation is dynamic during fiber differentiation^85^. Our study shows that DNA methylation, particularly CHH methylation, is highly dynamic and may play an important role in regulating gene expression during seed maturation. DNA methylation likely represents an integrated part of gene regulatory network underlying seed maturation.

Epigenetics has been shown to affect growth and development. After comparing methylation among leaf and seeds at the three maturation stages, we did not observe any obvious change in overall levels of CG and CHG methylation among the samples, but DNA methylation in CHH increased from 4% in leaves to 6%, 10%, and 11% in seeds at S2, S6, S8, representing a 50%, 150%, and 175% increase, respectively (Fig. 1a). Furthermore, the relative levels of methylated CHH (^m^CHH) among the total methylated cytosine in three contexts (^m^CG, ^m^CHG, and ^m^CHH) show the same trend. Methylated CHH levels among the total methylated cytosine were the lowest in leaf (22%) compared with those in seeds at three different stages (Fig. 1b). Relative levels of methylated CHH also increase as seeds mature: from 29% in S2 seed to 41% and 42% in S6 and S8 seeds, respectively (Fig. 1b). The change of methylation in the CHH context might represent dynamic epigenetic regulation and difference between plant vegetative growth and reproductive development. The relative levels of methylated CG were significantly higher in leaves (44%) than those in seeds at S2, S6, and S8 (32–39%). We found 3465 DMRs in 1641 genes that were mostly expressed specifically in leaves (Fig. S4). Most of these genes had low DNA methylation in promoter regions and high expression in leaf. The GO term analysis showed that genes involved in growth and fatty acid biosynthesis were over-represented among DMR genes in leaves (Table S7). This suggests that these epigenetically regulated genes can be critical for plant vegetative growth. It requires further investigation how these genes are epigenetically regulated and what functions these genes have in growth and development.

Centromeric and pericentromeric regions, which constitute a significant portion of soybean chromosomes, are methylated and silenced. TEs have been an integral part of plant genomes during millions of years of evolution. Kim *et al*. compared DNA methylomes in soybean and common bean by sequencing leaves and roots and discovered that DNA methylation plays a significant role in evolution of duplicated genes during pre- and post –whole-genome duplication, suggesting TEs and DNA methylation are involved in the evolution of genes in polyploidy and speciation^63^. In general, TEs are highly methylated, thus silenced in soybean seed (Fig. 2b). Plants have specific RNA polymerases IV, V, and RDR2 that are involved in generating 24-nt small interfering RNAs (siRNA) to induce DNA methylation in the RdDM pathway^40,44,45,86^. Recent research has shown that TEs can be expressed when DNA methylation is lost, produce 24-nt siRNA, and trigger targeted DNA methylation through the RdDM pathway^40^. We found 821 clusters in LTR TEs linked to 24-nt small RNA expression, and 499 clusters in TIR TEs linked to 24-nt small RNA expression (data not shown). These findings suggest that DNA methylation in TEs and RdDM is likely an integral part of overall epigenetic regulation or broad gene regulation in plant growth and development.

A recent study in soybean epigenome shows that DNA methylation also plays a role in regulating gene expression and gene evolution following polyploidy and speciation^63^. Very intriguingly, we found that DMRs occur more frequently in transcribed genes than non-transcribed genes (Table 3). During early seed maturation (S2 to S6), 27.52%, 0.72%, and 0.33% expressed genes have CHH-, CHG-, and CG- DMRs, while only 13.06%, 0.47%, and 0.17% non-transcribed genes had CHH-, CHG-, and CG- DMRs. The same trend was maintained during late seed maturation (S6 to S8) (Table 2). Why do DMRs appear more frequently in transcribed genes than non-transcribed genes? It would be interesting to know whether this phenomenon also occurs in other plant species. One speculation is that DMRs evolved more frequently in expressed genes as an additional mechanism to regulate gene expression during evolution. Examining DMR frequency in transcribed genes and non-transcribed genes in ancestor species, which evolved before soybean (*Glycine max*), may provide a clue for this speculation.

What can be the mechanism for dynamic changes of DNA methylation in genes and TEs during seed maturation? One can speculate that expression of DNA methyltransferases that are responsible for adding methyl group to the 5-position of cytosine undergoes a dynamic change during seed maturation. We examined expression of putative DNA methyltransferases *MET1*, *CMT*, *DRM* in soybean in our RNA-seq data, but did not observe an increased expression of these methyltransferase genes that could explain increased DNA methylation in the CHH context from S2 to S6 and S8. The other explanation is that a similar RdDM mechanism exists and is responsible for the dynamic CHH methylation changes in soybean. In the future, if we examine small RNA expression at different seed developmental stages and study their correlation with DNA methylation levels in genes and TEs, we might be able to illuminate the potential mechanism for the dynamic changes of DNA methylation.

### Experimental procedures

#### Plant materials

Soybean (*Glycine max* [L.] Merrill Cv. Jack) was grown in growth chambers at the following conditions: temperature: 25 °C day/ 23 °C night, humidity: 50%, and light: 16 hour per day. Based on fresh seed weight and color, cotyledon at S2 (green seed weighing 25–50 mg), S6 (green seed weighing 390–420 mg), and S8 (yellow seed weighing 200–250 mg) were harvested. Fully expanded leaves were collected. The harvested cotyledons and leaves were frozen in liquid nitrogen and stored at −80 °C until needed.

#### DNA and RNA library construction and sequencing

The bisulfite treatment, library construction and sequencing were conducted by the Beijing Genomics Institute (Shenzhen, China) as described in ref.^87^. Un-methylated lambda DNA was spiked in to determine non-conversion rate. The conversion rate for all libraries was higher than 99%. Six paired-end bisulfite-treated sample libraries were constructed and sequenced for two independent biological replicates for cotyledon at S2 and S6 stages, respectively, one for cotyledon at S8 stage and one for leaf. One library was constructed from untreated Jack DNA and sequenced as a control. Purification of total RNA and construction of RNA-seq libraries were performed as described in Goettel *et al*.^12^. Three transcriptome sequencing libraries representing three independent biological replicates were constructed for transcriptome sequencing for cotyledon at each seed stage. 100-bp paired-end reads were generated on the Illumina HiSeq. 2000 platform.

#### Sequence data processing and analysis

Untreated Jack genome sequencing reads were aligned to the Williams 82 v2 reference assembly (phytozome v10) using the Burrows-Wheeler Aligner with default parameters (Version: 0.7.12-r1039)^78,88^. SNPs were then filtered, excluding those with a read depth less than 5, those which clustered within 10 bases of each other, and those with a SNP calling quality score of less than 50. SNPs were called with the GATK’s UnifiedGenotyper (v2.1-13) and SAMtools mpileup (v0.1.19)^89,90^. The SNPs identified in *G. max* cv Jack were used to produce a Jack-corrected version of the *G. max* cv Williams 82 reference assembly phytozome v10. The Bismark alignment software (Version: v0.14.3)^91^ with the default parameters were used to make the initial alignments against the Jack-corrected reference genome and to produce methylation calls. Genome-wide cytosine methylation reports for each methylation context were generated with the Bismark methylation extractor with the parameters (–paired-end–no_overlap–comprehensive–ignore 2–ignore_r2 2–ignore_3prime 6–ignore_3prime_r2 6). Alignments for the bisulfite sequence reads required both mates of each read pair to align in a single unique location.

Methylation level for each given DNA region was expressed as its percent methylation (total number of bases that were methylated divided by the total number of bases evaluated for methylation) in each context. The averaged value from two replicates was used for S2 and S6 stages respectively. The average percent methylation in gene bodies of all genes was calculated using phytozome v10 gene annotations Gmax_275_Wm82.a2.v1.gene.gff3^92^. Each gene body (including UTR, CDS, and INTRONS) was divided into 100 equally sized bins and the average methylation level in percentage was derived for each bin. The 4-kb 5′ and 3′ flanking regions were treated likewise by splitting each into 100 40-bp bins.

For identifying differentially methylated region (DMR), percent methylation for each context was calculated in adjacent 100-bp windows scanning across promoter region (4-kb upstream regions of coding genes) in each sample, and the average percent methylation for two replicates was then calculated for cotyledons at S2 and S6 stages respectively. 100-bp DNA regions (windows) with more than 30% difference in their methylation levels between two compared stages were defined as differentially methylated regions. A gene containing a DMR was defined as a DMR gene. If no more than 30% methylation difference was detected in cotyledon at different development stages, comparisons were made between each cotyledon stage and leaf. A 30% difference between cotyledon at each maturation stage and leaf was required to be considered a significant leaf DMR in these comparisons.

RNA sequence processing and analysis was conducted as described by^93^. CASAVA 1.8.2 (Illumina, Inc., San Diego, CA) was used to produce purity-filtered reads from sequencing RNA libraries. Purity-filtered RNA-Seq reads from each of the three replicates for each maturation stage were used for alignment with tophat2 v2.0.10. The transcript accumulation level for each gene noted in FPKMs was derived using Cufflinks and Cuffdiff v2.2.1. One-way ANOVA in the R statistical package (version 2.11.1) was used to compare transcript accumulation for each gene in cotyledons at different maturation stages to identify differentially regulated genes with P value of 0.05 as a cut-off.

The Person Correlation Coefficient (PCC) was used to quantitate the relationship between expression and methylation levels for DMR genes that were differentially expressed by more than two fold and with a P value less than 0.05 between any compared stages. Genes with a PCC value less than −0.85 were used in clustering analysis. DMR genes at each methylation context were grouped and clustered separately. The R package mclust (https://www.R-project.org) was used to determine the model-based optimal number of clusters to use. The log2-transfromed expression levels in FPKM were loaded into Cluster 3; the expression levels were adjusted to center genes on the mean expression; and then k-means clustering was performed using Euclidean distance and the optimal number of clusters previously defined. The heat map resulting from clustering was viewed using Java Treeview^94^.

#### Disclaimer note

Names are necessary to report factually on available data; however, the USDA neither guarantees nor warrants the standard of the product, and the use of the name by USDA implies no approval of the product to the exclusion of others that may also be suitable. USDA is an equal opportunity provider and employer.

## Electronic supplementary material

Supplementary Info
