# Author Correction: Dynamic Changes of Genome-Wide DNA Methylation during Soybean Seed Development

**DOI:** 10.1038/s41598-018-25805-x

**Published:** 2018-05-15

**Authors:** Yong-qiang Charles An, Wolfgang Goettel, Qiang Han, Arthur Bartels, Zongrang Liu, Wenyan Xiao

**Affiliations:** 1 US Department of Agriculture, Agricultural Research Service, Midwest Area, Plant Genetics Research Unit, Donald Danforth Plant Science Center, St. Louis, MO 63132 USA; 2 Department of Biology, Saint Louis University, St. Louis, MO 63103 USA; 3 US Department of Agriculture, Agricultural Research Service, Appalachian Fruit Research Station, Kearneysville, WV 25430 USA

Correction to: *Scientific Reports* 10.1038/s41598-017-12510-4, published online 25 September 2017

In Figure 4, the labels ‘S2, S6, S8’ are incorrectly given as ‘S2, S4, S6’. The correct Figure 4 appears below as Figure 1.

**Figure 1:**
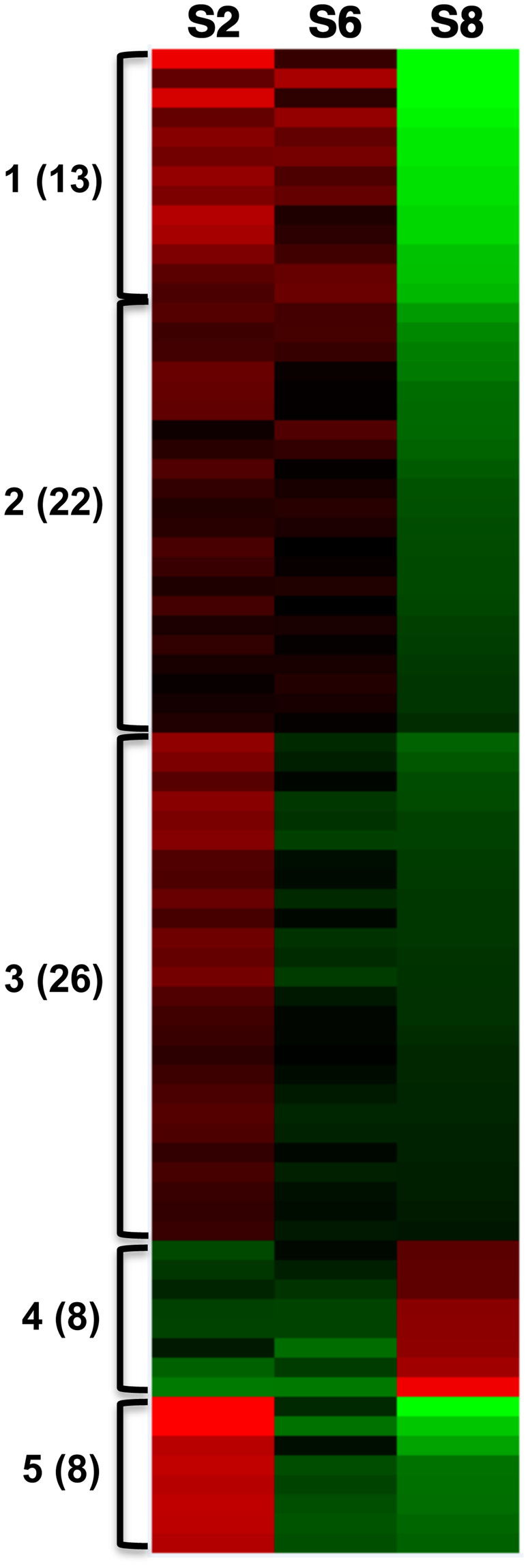
Cluster analysis of 77 genes with seed-specific CHH DMRs based on gene expression at stages S2, S6, and S8. Gene clusters based on gene transcription patterns in cotyledon at three stages and DNA methylation in DMRs in ^m^CHH, ^m^CHG and ^m^CG contexts. The green to red color gradient represents low to high gene expression, respectively. Genes with 1) more than 30% DNA methylation changes among three different seed stages S2, S6, and S8, 2). statistically significant changes in gene expression and 3). a negative correlation (PCC < −0.85) between gene expression and methylation level were used for cluster analysis.

