# Supplementary material for: Dynamic Changes of Genome-Wide DNA Methylation during Soybean Seed Development
Source: Sci Rep. 2017 Sep 25;7:12263. doi: 10.1038/s41598-017-12510-4 (PMC5613027; doi:10.1038/s41598-017-12510-4)
Supplement: Supplementary file 1 — Supplementary Info [file 41598_2017_12510_MOESM1_ESM.pdf]

Supplementary information

Dynamic Changes of Genome-Wide DNA Methylation during Soybean Seed Development

Yong-qiang Charles An[1*], Wolfgang Goettel[1], Qiang Han[2], Arthur Bartels[2], Zongrang Liu[3], Wenyan Xiao[2*]

[1]US Department of Agriculture, Agricultural Research Service, Midwest Area, Plant Genetics Research Unit, Donald Danforth Plant Science Center, MO 63132 USA

[2]Department of Biology, Saint Louis University, St. Louis, MO 63103 USA

[3]US Department of Agriculture, Agricultural Research Service, Appalachian Fruit Research Station, Kearneysville, WV 25430 USA

*Corresponding authors: Yong-qiang Charles An, Phone: 314-587-1669, Email: yong-qiang.an@ars.usda.gov; Wenyan Xiao, Phone: 314-977-2547, Email: wxiao@slu.edu

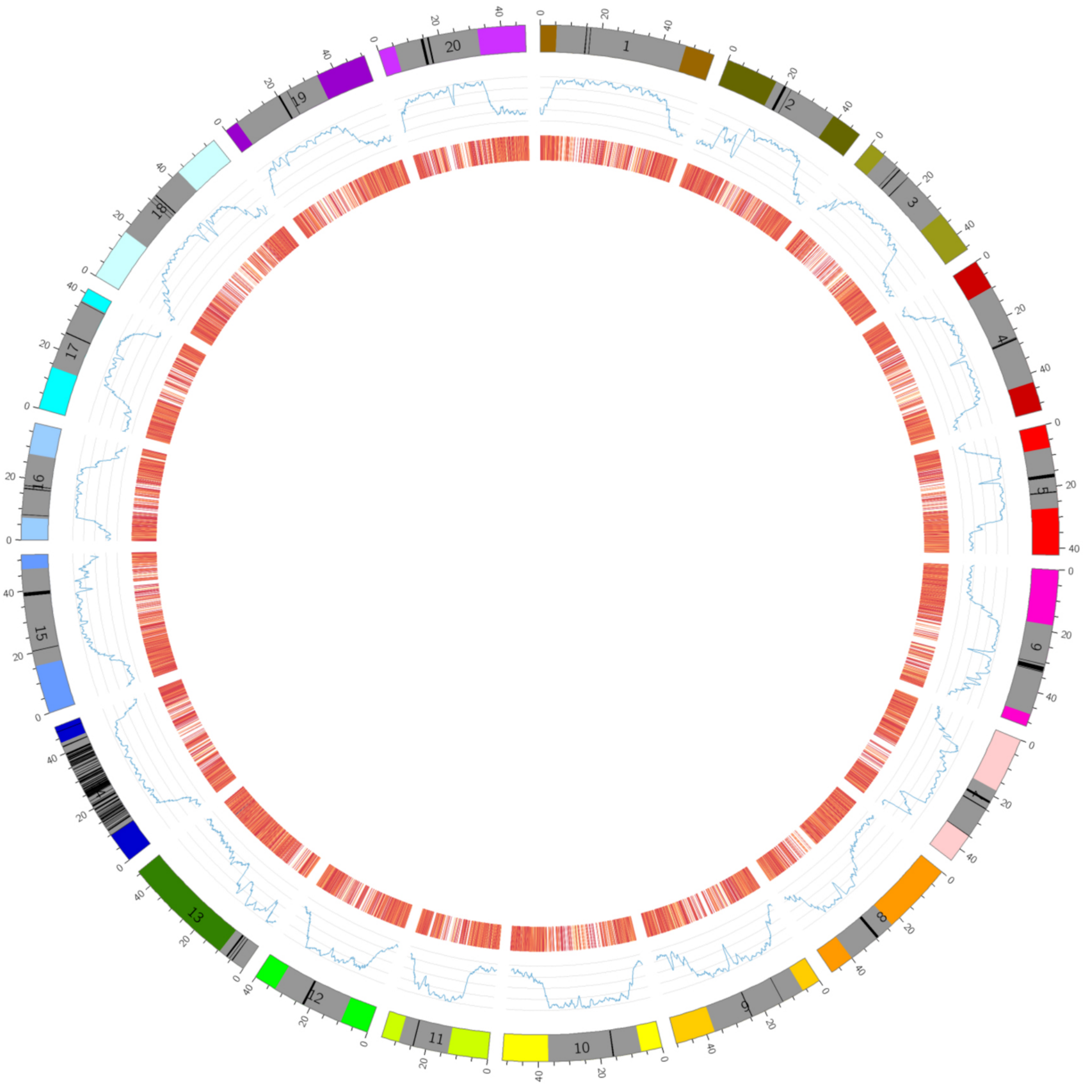

Figure S1

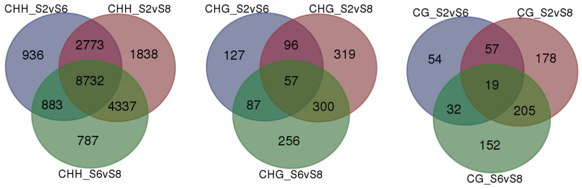

CHH_S2vS6
CHH_S2vS8
936
2773
1838
8732
883
4337
787
CHH_S6vS8
CHG_S2vS6
CHG_S2vS8
127
96
319
57
87
300
256
CHG_S6vS8
CG_S2vS6
CG_S2vS8
54
57
178
19
32
205
152
CG_S6vS8

Figure S2

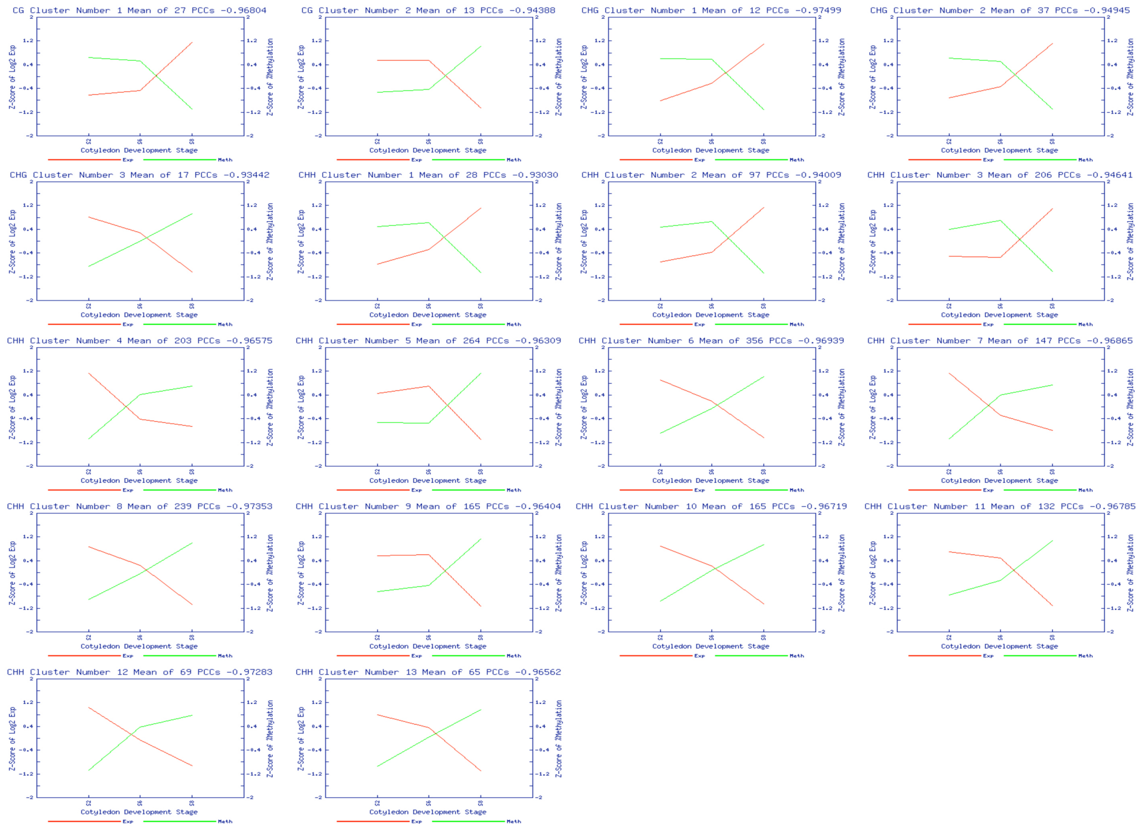
CG Cluster Number 1 Mean of 27 PCCs -0.96804
CG Cluster Number 2 Mean of 13 PCCs -0.94388
CHG Cluster Number 1 Mean of 12 PCCs -0.97499
CHG Cluster Number 2 Mean of 37 PCCs -0.94945
CHG Cluster Number 3 Mean of 17 PCCs -0.93442
CHH Cluster Number 1 Mean of 28 PCCs -0.93030
CHH Cluster Number 2 Mean of 97 PCCs -0.94009
CHH Cluster Number 3 Mean of 206 PCCs -0.94641
CHH Cluster Number 4 Mean of 203 PCCs -0.96575
CHH Cluster Number 5 Mean of 264 PCCs -0.96309
CHH Cluster Number 6 Mean of 356 PCCs -0.96939
CHH Cluster Number 7 Mean of 147 PCCs -0.96865
CHH Cluster Number 8 Mean of 239 PCCs -0.97353
CHH Cluster Number 9 Mean of 165 PCCs -0.96404
CHH Cluster Number 10 Mean of 165 PCCs -0.96719
CHH Cluster Number 11 Mean of 132 PCCs -0.96785
CHH Cluster Number 12 Mean of 69 PCCs -0.97283
CHH Cluster Number 13 Mean of 65 PCCs -0.96562
Z-Score of Log2 Exp
Z-Score of %Methylation
Cotyledon Development Stage
Exp
Meth

Figure S3

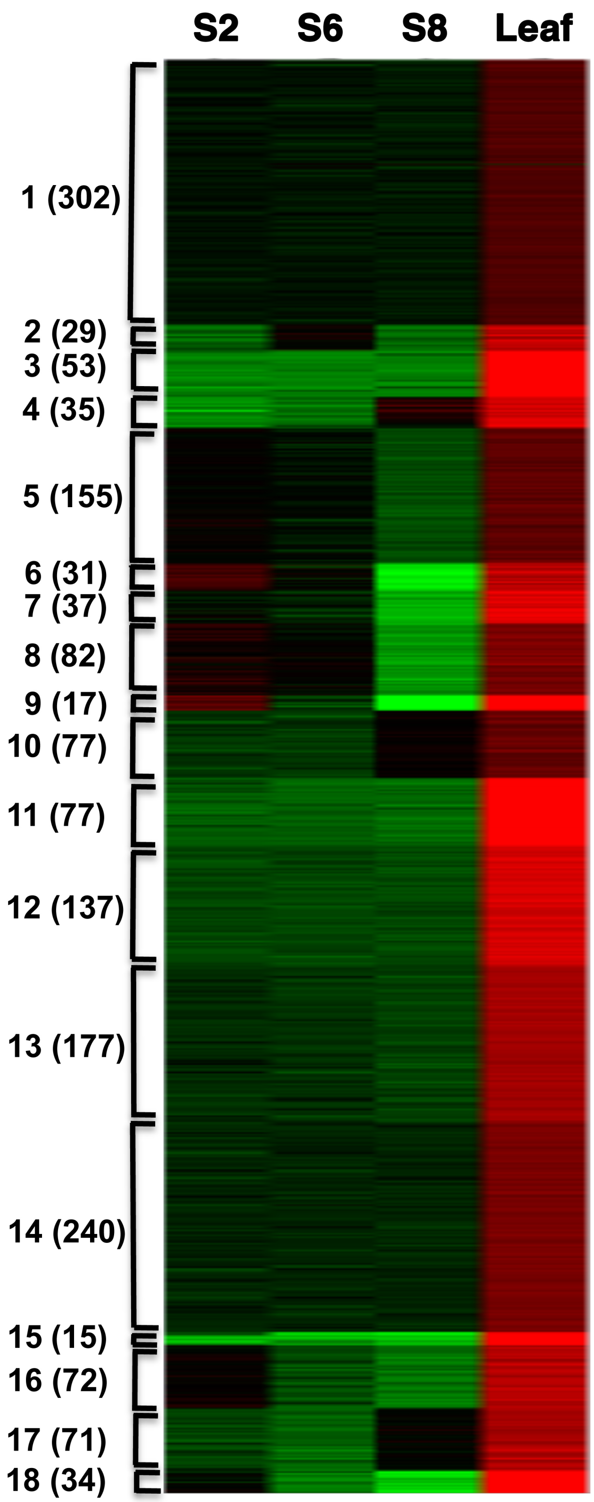
S2
S6
S8
Leaf
1 (302)
2 (29)
3 (53)
4 (35)
5 (155)
6 (31)
7 (37)
8 (82)
9 (17)
10 (77)
11 (77)
12 (137)
13 (177)
14 (240)
15 (15)
16 (72)
17 (71)
18 (34)
Figure S4

The supplementary figure legends

Figure S1. **Genome-wide features of CHH DNA methylation landscapes and transcriptome of soybean seed.** Figure S1 was drawn by using the same data as Figure 1 by deleting CG and CHG methylation data. Figure S1 showed percentage of CHH methylation in the blue line rescaled from a minimum of 1% to a maximum of 20% with grey lines at 5% increments.

Figure S2. DMRs in CHH, CHG, CG contexts during seed maturation process. Venn diagrams show overlapping DMR genes between stages S2 and S6, between S2 and S8, and between S6 and S8 in CHH, CHG, and CG contexts.

Figure S3. Relationship between DNA methylation and gene transcription on the whole genome scale. Z-scores of DNA methylation in the promoter regions were plotted against the corresponding Z-scores of log_2 converted expression values for clustered genes at S2, S6, and S8 stages. The linear correlation equation was y = -0.9545 x + 4E-15 with R^2 = 0.9112.

Figure S4. Cluster analysis of genes with CHH DMRs and high expression in leaf. To identify genes that have DMRs and differential expression pattern between leaf and seed, genes with larger than 30% in DNA methylation differences between leaf and seeds at three different seed stages S2, S6, and S8, and with significant change in gene expression (the ANOVA test) that was negatively correlated with DNA methylation (PCC value smaller than -0.8), were found and then a cluster analysis was performed.

Table S1. Raw data of bisulfite sequencing of soybean methylome in seeds and leaves

Sample	%Reads Aligned*	Average Depth of covered bases	% of Covered Bases (978,495,272)
**S2_1**	72.0	8.8	90.8
**S2_2**	71.9	8.8	90.2
**S6_1**	73.6	9.1	89.3
**S6_2**	71.9	8.8	89.5
**S8**	71.8	8.9	88.5
**Leaf**	69.9	8.5	90.2
**Jack Reference**	91.4	10.9	92.9

***Alignments for the bisulfite sequence require both mates for read pairs to align in a single unique location making % aligned lower.**

Table S2. DNA methylation percentage of soybean methylome in seed and leaf samples

	Leaf	S2	S6	S8	Average Three Seed Stages
**Methylation in CG**	63.2%	65.6%	65.1%	67.0%	65.9%
**Methylation in CHG**	38.4%	44.1%	44.7%	47.2%	45.3%
**Methylation in CHH**	4.1%	6.2%	10.4%	11.0%	9.2%

Table S3. Coefficient of Variation (CV, %) among S2, S6, and S8 stages for CG, CHG, and CHH contexts

Gene/TEs	CG	CHG	CHH
**Genes**	1.5	3.4	29.6
**Helitron**	5.4	8.0	30.7
**LINE**	4.1	4.9	28.5
**LTR**	0.9	0.7	27.1
**TIR**	1.0	1.9	26.7
**Average**	2.6	3.8	28.5

Table S4. The 2136 genes with CHH DMRs during seed maturation

Genes	Log2 S2	Log2 S6	Log2 S8	S2 %Meth	S6 %Meth	S8 %Meth	PCC
**Glyma.01G002600**	3.06	2.46	0.56	0	0	60.9	-0.91
**Glyma.01G008900**	2.36	2.74	1.51	0	0	47.6	-0.92
**Glyma.01G009700**	4.17	2.39	0.25	29.8	55.9	93.1	-0.92
**Glyma.01G015500**	4.24	4.37	5.38	63.9	72.7	42.4	-0.94
**Glyma.01G016500**	1.52	1.04	0	0	0	47.4	-0.90
**Glyma.01G020900**	4.83	4.75	0.13	20.3	45.1	73.0	-0.91
**Glyma.01G022500**	3.26	3.12	5.11	36.5	53.6	15.0	-0.91
**Glyma.01G023000**	1.32	0.18	0.74	32.8	67.6	48.7	-0.99
**Glyma.01G025200**	1.54	0.81	0.03	48.2	75.7	100.0	-1.00
**Glyma.01G026700**	3.14	2.93	1.29	11.1	31.8	66.7	-0.98
**Glyma.01G026800**	4.34	3.17	2.21	5.0	33.3	62.5	-0.97
**Glyma.01G028200**	2.17	1.73	0.20	12.6	33.1	63.0	-1.00
**Glyma.01G028700**	4.39	3.88	1.21	7.7	39.9	93.3	-1.00
**Glyma.01G028900**	3.35	2.89	2.09	7.1	26.4	57.7	-1.00
**Glyma.01G031300**	2.10	0.29	0.01	47.4	86.6	90.0	-1.00
**Glyma.01G032000**	1.96	1.53	0.06	0	0	43.6	-0.94
**Glyma.01G034900**	3.34	3.41	0.13	7.5	31.8	72.7	-0.91
**Glyma.01G035600**	2.14	1.72	0.09	9.3	20.4	44.4	-1.00
**Glyma.01G038900**	1.21	0.85	0.01	48.2	69.2	85.0	-0.98
**Glyma.01G040000**	3.61	2.47	0.17	5.3	22.0	40.6	-0.99
**Glyma.01G040300**	3.77	3.54	5.50	68.9	83.3	30.8	-0.98
**Glyma.01G040600**	1.51	1.00	0.09	11.5	45.5	100.0	-0.99
**Glyma.01G044000**	6.61	5.93	1.16	40.0	54.9	88.0	-1.00
**Glyma.01G046000**	2.08	1.06	0.01	35.0	57.5	85.0	-0.97
**Glyma.01G046300**	4.56	3.73	0.17	8.6	37.7	68.0	-1.00
**Glyma.01G046400**	1.51	1.08	0.14	31.3	64.6	70.8	-0.90
**Glyma.01G050100**	2.65	1.26	0.05	18.8	50.0	68.8	-0.99
**Glyma.01G053100**	1.21	0.71	0.01	64.8	72.4	96.6	-0.95
**Glyma.01G053300**	2.81	2.44	1.01	0	0	30.0	-0.95
**Glyma.01G056100**	6.19	5.49	0.42	22.9	52.1	66.7	-0.95
**Glyma.01G056300**	2.73	2.63	1.28	8.3	6.0	56.0	-0.99
**Glyma.01G058000**	3.96	3.23	1.56	0	0	30.4	-0.87
**Glyma.01G067900**	3.22	2.54	2.04	0	38.9	55.6	-1.00
**Glyma.01G068900**	0.54	0.45	1.92	20.0	30.0	0	-0.95
**Glyma.01G078800**	2.88	2.22	0.17	12.5	22.5	45.0	-0.99
**Glyma.01G079500**	3.42	3.34	1.28	2.1	10.4	41.7	-0.99
**Glyma.01G080900**	3.01	2.80	0.31	5.0	20.0	36.8	-0.94
**Glyma.01G081100**	1.70	1.11	0.13	12.5	37.1	62.5	-1.00
**Glyma.01G081300**	6.27	4.89	1.40	2.4	30.4	60.0	-0.99

**Glyma.01G081900**	2.86	0.42	0.01	27.4	61.8	55.9	-0.98
**Glyma.01G084600**	3.06	2.29	0.30	19.2	54.9	89.5	-1.00
**Glyma.01G086900**	2.75	2.55	0.78	2.4	33.3	66.7	-0.94
**Glyma.01G089800**	1.12	0.13	0	17.3	52.7	57.7	-1.00
**Glyma.01G092900**	1.24	0.59	0	13.9	38.3	45.5	-0.99
**Glyma.01G102400**	3.33	3.39	1.75	8.9	12.5	39.3	-0.99
**Glyma.01G104100**	1.56	0.82	5.31E-04	25.7	36.5	88.5	-0.89
**Glyma.01G106200**	4.47	4.05	2.02	5.0	8.3	47.4	-0.97
**Glyma.01G115900**	7.37	6.36	5.36	11.7	48.1	57.7	-0.99
**Glyma.01G118900**	2.63	3.04	1.67	31.4	16.8	60.0	-1.00
**Glyma.01G121200**	1.67	1.12	0	2.2	7.7	38.1	-0.94
**Glyma.01G121700**	2.32	0.47	0.04	31.3	100.0	72.2	-0.88
**Glyma.01G125800**	2.39	0.39	0.01	1.7	30.0	41.4	-0.98
**Glyma.01G133400**	6.48	5.55	4.26	36.7	55.0	75.9	-0.99
**Glyma.01G136300**	1.10	1.62	0.31	7.1	0	45.5	-0.93
**Glyma.01G137500**	0.57	3.05	0.53	18.8	0	37.5	-0.87
**Glyma.01G139000**	2.86	2.73	1.75	6.8	11.5	39.3	-1.00
**Glyma.01G144200**	2.00	1.78	0.25	3.1	10.0	52.9	-1.00
**Glyma.01G144900**	3.85	3.36	2.49	3.8	7.1	60.0	-0.91
**Glyma.01G145000**	4.29	3.52	2.23	5.8	20.8	37.5	-1.00
**Glyma.01G146600**	2.65	2.13	1.03	7.1	14.3	38.1	-0.97
**Glyma.01G150700**	3.09	3.03	1.26	25.9	32.1	66.7	-1.00
**Glyma.01G152000**	2.38	1.53	0	20.3	59.4	70.8	-0.97
**Glyma.01G153200**	5.32	5.82	4.73	23.6	15.9	47.1	-0.94
**Glyma.01G160300**	2.72	1.44	0.10	63.0	97.8	91.3	-0.89
**Glyma.01G162800**	4.43	3.72	4.81	23.3	61.5	15.4	-0.96
**Glyma.01G162900**	2.00	1.89	0.74	0	0	41.7	-0.99
**Glyma.01G164300**	3.22	2.74	1.10	27.8	61.1	68.8	-0.88
**Glyma.01G166100**	1.28	0.95	0.25	0	0	45.5	-0.92
**Glyma.01G167500**	3.31	3.42	2.04	16.2	12.9	42.9	-1.00
**Glyma.01G175900**	3.67	3.70	2.68	50.0	67.7	100.0	-0.93
**Glyma.01G180400**	4.65	2.33	2.46	16.5	52.2	50.0	-1.00
**Glyma.01G185200**	2.73	2.36	4.04	41.7	31.0	0	-0.93
**Glyma.01G186800**	6.62	5.88	0.63	0	0	40.0	-0.91
**Glyma.01G188000**	2.01	1.80	0.80	0	30.0	60.0	-0.96
**Glyma.01G189100**	2.00	2.01	3.39	54.5	48.5	21.2	-0.99
**Glyma.01G189500**	2.12	2.07	0.22	38.7	50.3	80.8	-0.97
**Glyma.01G189600**	1.23	1.21	2.90	77.8	96.3	59.3	-0.87
**Glyma.01G190000**	3.33	1.12	0.01	11.1	36.1	45.5	-0.99
**Glyma.01G193500**	1.45	0.58	0.25	0	26.5	33.3	-1.00
**Glyma.01G199600**	2.47	2.51	0.23	48.9	48.7	86.7	-1.00
**Glyma.01G199700**	5.31	2.81	0.42	37.5	72.3	80.0	-1.00
**Glyma.01G203600**	6.56	5.58	3.90	9.9	41.7	55.6	-0.99

**Glyma.01G212900**	2.17	2.01	0.93	17.6	25.9	53.6	-1.00
**Glyma.01G213200**	3.39	3.10	0.32	26.3	36.4	57.9	-0.99
**Glyma.01G214100**	1.30	1.11	3.04	29.7	43.3	0	-0.96
**Glyma.01G215700**	1.36	1.56	0.04	0	0	30.0	-0.98
**Glyma.01G226600**	1.76	1.79	0.27	38.9	34.8	74.1	-1.00
**Glyma.01G226700**	3.41	2.55	1.71	17.6	29.4	82.4	-0.87
**Glyma.01G236400**	1.33	1.86	0.52	12.5	6.7	50.0	-0.92
**Glyma.01G245500**	3.89	4.20	2.31	25.6	15.4	50.0	-1.00
**Glyma.02G000900**	5.39	4.71	4.07	19.5	50.8	51.5	-0.94
**Glyma.02G001700**	1.66	1.25	0.20	4.0	7.7	36.8	-0.96
**Glyma.02G002500**	2.23	1.82	1.00	11.1	47.2	72.7	-0.98
**Glyma.02G003800**	1.72	0.65	0.05	48.8	86.0	89.7	-0.99
**Glyma.02G004400**	3.61	2.80	2.40	3.3	19.2	47.4	-0.90
**Glyma.02G005600**	1.13	0.14	0.02	59.3	90.5	81.5	-0.94
**Glyma.02G025400**	1.39	1.57	0.10	37.9	43.4	79.3	-0.96
**Glyma.02G026800**	3.13	2.54	0.41	0	0	40.0	-0.92
**Glyma.02G030200**	4.70	4.03	0.23	0	0	40.7	-0.92
**Glyma.02G031200**	1.40	0.91	0.15	0	2.1	90.5	-0.88
**Glyma.02G033400**	2.44	1.98	9.49E-06	0	12.5	31.3	-1.00
**Glyma.02G034000**	0.59	0.37	2.04	32.4	55.9	5.9	-0.91
**Glyma.02G035600**	6.91	6.51	4.71	38.6	50.0	70.8	-1.00
**Glyma.02G036300**	5.01	4.70	3.59	33.3	66.7	100.0	-0.98
**Glyma.02G040700**	3.02	1.99	0.63	7.7	50.4	55.2	-0.96
**Glyma.02G041100**	3.36	4.09	0.08	0	0	43.8	-0.91
**Glyma.02G042500**	0.18	0.24	3.91	37.0	51.2	13.0	-0.93
**Glyma.02G045400**	2.71	2.72	1.63	0	7.5	35.7	-0.98
**Glyma.02G050200**	3.86	3.46	2.04	25.3	41.7	70.0	-1.00
**Glyma.02G051300**	1.29	1.18	0.12	8.3	25.8	52.2	-0.96
**Glyma.02G057200**	4.30	3.81	0.27	0	0	52.9	-0.95
**Glyma.02G062700**	2.72	0.94	0.73	17.2	65.4	100.0	-0.93
**Glyma.02G075100**	5.73	5.03	2.14	0	0	33.3	-0.91
**Glyma.02G080100**	1.07	3.86E-04	1.12	14.8	56.5	31.8	-0.89
**Glyma.02G083900**	0.43	1.06	1.56	36.0	5.2	0	-0.91
**Glyma.02G084800**	3.31	0.26	0.05	0	30.1	15.8	-0.87
**Glyma.02G085500**	3.97	3.79	2.84	19.1	39.8	50.0	-0.87
**Glyma.02G087300**	4.12	3.23	0.14	0	19.9	40.0	-1.00
**Glyma.02G087400**	0.65	0.61	2.80	61.9	56.7	23.8	-0.99
**Glyma.02G090800**	4.62	5.58	9.51	53.3	74.6	29.4	-0.87
**Glyma.02G091200**	3.93	4.33	6.46	28.6	42.9	7.1	-0.89
**Glyma.02G093100**	2.31	0.63	0	41.8	77.3	93.8	-0.98
**Glyma.02G095200**	2.84	3.01	1.06	20.9	30.7	52.9	-0.91
**Glyma.02G097000**	3.54	2.44	0.27	60.7	85.7	100.0	-1.00
**Glyma.02G097400**	1.22	0.97	0.15	1.9	0	31.3	-0.94

**Glyma.02G099100**	1.82	0.50	0.22	3.6	75.0	66.7	-0.98
**Glyma.02G100800**	1.48	0.65	0.05	5.6	35.2	44.4	-1.00
**Glyma.02G101300**	3.03	3.37	0.57	2.0	0	69.6	-0.98
**Glyma.02G104200**	3.22	3.84	4.98	82.9	85.0	45.8	-0.96
**Glyma.02G105500**	4.04	3.59	1.09	32.1	45.3	75.0	-1.00
**Glyma.02G109100**	2.17	1.03	0.07	40.0	85.0	77.8	-0.91
**Glyma.02G111600**	2.24	1.01	0.05	24.2	81.4	90.5	-0.99
**Glyma.02G114200**	1.87	2.91	1.73	26.8	8.9	50.0	-0.87
**Glyma.02G114800**	3.17	3.33	1.67	0	0	39.3	-0.99
**Glyma.02G120500**	3.58	4.09	5.29	68.5	81.7	23.3	-0.92
**Glyma.02G126200**	1.47	1.33	0.02	11.0	21.3	44.4	-0.98
**Glyma.02G130100**	0.45	0.14	1.28	21.5	47.6	0	-0.93
**Glyma.02G132100**	0.65	1.96	4.38	72.9	80.0	37.1	-0.96
**Glyma.02G136700**	0.62	0.77	4.92	48.6	55.2	20.0	-0.98
**Glyma.02G137400**	3.63	3.32	0.11	10.7	3.6	58.3	-0.95
**Glyma.02G146600**	2.33	3.34	4.51	36.6	43.8	3.2	-0.90
**Glyma.02G147200**	1.91	0.62	0.13	8.0	30.0	40.0	-0.99
**Glyma.02G149700**	4.26	4.12	2.14	6.5	15.2	39.1	-0.99
**Glyma.02G166300**	2.37	2.24	0.88	15.5	30.3	95.7	-1.00
**Glyma.02G168000**	3.31	3.33	2.16	43.7	56.8	81.8	-0.94
**Glyma.02G169000**	1.42	0.65	0	2.4	40.8	69.0	-1.00
**Glyma.02G171300**	2.76	2.36	1.30	17.3	26.1	57.1	-0.99
**Glyma.02G174300**	3.03	2.32	1.77	6.7	28.6	42.9	-1.00
**Glyma.02G175400**	1.06	1.13	0.05	0	8.3	33.3	-0.95
**Glyma.02G177500**	1.65	1.57	0.24	9.6	18.3	64.3	-1.00
**Glyma.02G182200**	1.05	0.17	8.90E-06	13.0	61.1	50.0	-0.95
**Glyma.02G184800**	6.18	5.31	3.42	1.9	24.2	33.3	-0.98
**Glyma.02G186600**	1.77	0.50	6.22E-08	30.4	63.9	70.0	-1.00
**Glyma.02G189100**	2.59	2.04	0.71	0	13.6	40.9	-0.99
**Glyma.02G189600**	3.39	3.13	1.20	8.7	24.6	45.8	-0.97
**Glyma.02G191100**	1.03	1.14	4.87E-03	7.7	2.1	52.9	-1.00
**Glyma.02G192200**	1.56	0.52	0.03	4.0	28.0	50.0	-0.96
**Glyma.02G192500**	2.57	2.51	4.21	42.9	49.9	11.1	-0.99
**Glyma.02G198400**	5.27	3.48	3.16	7.9	54.8	57.1	-1.00
**Glyma.02G200300**	4.38	3.19	3.63	26.5	64.5	78.3	-0.86
**Glyma.02G202900**	1.01	2.43	3.03	78.5	74.1	48.3	-0.90
**Glyma.02G203900**	3.39	2.60	1.47	16.0	53.5	80.0	-1.00
**Glyma.02G206500**	1.08	1.25	2.40	30.1	50.0	5.7	-0.86
**Glyma.02G206800**	4.02	3.72	2.82	12.9	32.0	60.0	-1.00
**Glyma.02G208400**	4.61	4.59	2.77	7.1	4.8	63.6	-1.00
**Glyma.02G208900**	5.22	4.85	4.08	4.8	28.3	67.7	-1.00
**Glyma.02G209000**	3.58	1.87	0.16	3.8	65.9	95.8	-1.00
**Glyma.02G209400**	0.24	0.89	1.84	45.5	45.9	0	-0.96

**Glyma.02G211000**	0.07	0.17	1.98	62.3	67.1	10.0	-1.00
**Glyma.02G215000**	2.86	1.29	0.07	49.1	75.0	92.9	-0.98
**Glyma.02G216800**	1.05	2.41	3.18	36.2	33.3	0	-0.92
**Glyma.02G217300**	1.10	0.55	2.25	64.3	65.2	34.8	-0.99
**Glyma.02G218300**	1.14	0.59	0	6.5	47.4	58.6	-0.97
**Glyma.02G228400**	2.68	1.83	1.08	0	47.5	46.7	-0.94
**Glyma.02G228700**	2.73	2.53	1.17	23.1	25.0	73.7	-0.99
**Glyma.02G229300**	4.26	4.56	0.40	16.1	13.4	55.0	-0.99
**Glyma.02G230500**	5.63	4.40	0.96	17.9	38.1	51.9	-1.00
**Glyma.02G231800**	3.54	3.37	2.49	16.7	24.1	47.4	-1.00
**Glyma.02G238200**	2.61	2.31	1.25	22.3	20.7	55.2	-0.94
**Glyma.02G239000**	1.99	0.33	4.31E-03	42.9	64.3	85.7	-0.90
**Glyma.02G239500**	2.60	1.34	4.32	53.3	51.1	0	-0.97
**Glyma.02G240300**	2.18	1.35	2.83	75.0	78.6	34.3	-0.93
**Glyma.02G250200**	4.35	4.50	1.79	5.7	13.4	58.6	-0.97
**Glyma.02G250400**	3.39	2.22	0.43	15.5	46.6	41.4	-0.87
**Glyma.02G254300**	1.35	2.67	5.90	59.1	83.1	25.0	-0.89
**Glyma.02G258900**	7.00	6.11	4.63	3.5	22.8	41.4	-1.00
**Glyma.02G266300**	3.13	3.59	5.02	81.8	79.8	46.2	-1.00
**Glyma.02G266700**	1.75	0.69	0.29	50.0	89.7	76.5	-0.87
**Glyma.02G268600**	3.61	3.49	2.56	0	0	77.3	-0.99
**Glyma.02G273100**	3.68	3.15	1.64	0	0	54.6	-0.92
**Glyma.02G274900**	6.03	5.04	0.96	0	20.0	40.0	-1.00
**Glyma.02G275300**	3.81	3.47	2.61	0	7.1	42.9	-0.98
**Glyma.02G281100**	3.33	1.63	0.25	8.9	49.3	53.9	-0.99
**Glyma.02G281400**	6.49	3.49	0.87	26.1	86.6	100.0	-1.00
**Glyma.02G287300**	1.69	0.89	0.01	46.4	60.7	80.8	-0.97
**Glyma.02G290100**	1.46	2.17	2.84	62.1	63.2	26.3	-0.91
**Glyma.02G291300**	1.45	1.73	3.82	75.0	76.1	21.9	-1.00
**Glyma.02G294500**	3.89	3.22	2.54	39.6	72.9	75.0	-0.94
**Glyma.02G295100**	5.28	4.68	0.57	0	0	70.0	-0.94
**Glyma.02G296400**	2.95	2.68	0.82	37.5	77.1	95.0	-0.87
**Glyma.02G302500**	6.63	5.46	4.68	45.0	80.0	75.0	-0.93
**Glyma.02G302700**	1.02	0.64	3.54E-04	1.9	32.1	46.4	-0.97
**Glyma.02G304000**	2.12	1.79	0.93	11.1	23.6	45.5	-1.00
**Glyma.02G305500**	4.13	4.20	0.41	54.1	50.8	88.0	-1.00
**Glyma.02G308100**	2.38	0.98	0.64	30.9	73.5	61.8	-0.93
**Glyma.03G000900**	2.18	1.45	0.95	20.8	76.6	91.7	-0.99
**Glyma.03G001000**	0.95	1.36	0.09	41.2	33.7	65.5	-0.98
**Glyma.03G002500**	2.96	2.91	1.67	0	4.6	36.4	-1.00
**Glyma.03G002900**	1.50	1.11	0.18	29.3	41.4	65.5	-1.00
**Glyma.03G005300**	1.83	1.24	1.93E-05	0	33.2	58.6	-0.99
**Glyma.03G008100**	2.55	2.05	0.07	0	11.3	47.8	-0.99

**Glyma.03G009700**	1.64	1.65	0.61	11.1	6.0	42.4	-0.99
**Glyma.03G018500**	3.01	2.79	3.85	65.2	84.6	45.0	-0.93
**Glyma.03G030800**	3.61E-06	1.00	5.74	42.5	34.6	0	-0.99
**Glyma.03G031900**	1.79	0.40	2.63	27.3	46.4	5.3	-1.00
**Glyma.03G033200**	1.13	0.74	1.87	35.0	40.1	0	-0.99
**Glyma.03G035600**	3.20	3.07	0.80	8.6	22.2	40.0	-0.94
**Glyma.03G041800**	3.05	3.06	1.78	9.4	34.4	61.3	-0.87
**Glyma.03G056700**	2.11	1.63	0.13	18.8	31.3	54.2	-1.00
**Glyma.03G059200**	1.23	1.28	0.24	0	5.0	30.0	-0.98
**Glyma.03G065900**	4.77	3.10	0.13	19.7	45.2	92.1	-0.92
**Glyma.03G068000**	1.41	0.76	0.23	6.7	57.4	70.4	-0.99
**Glyma.03G070200**	2.78	2.41	4.17	20.3	40.5	2.7	-0.90
**Glyma.03G072200**	0	0	1.14	36.3	28.8	2.5	-0.98
**Glyma.03G074400**	6.72	5.68	4.57	12.0	39.1	59.4	-0.99
**Glyma.03G076100**	5.57	4.40	2.37	21.6	41.9	68.0	-0.98
**Glyma.03G078700**	2.14	1.04	0.34	29.5	50.0	80.0	-0.93
**Glyma.03G079800**	2.72	2.47	0.40	7.9	29.1	56.7	-0.97
**Glyma.03G080100**	3.55	2.24	0.81	11.8	40.1	47.1	-0.99
**Glyma.03G082900**	3.88	3.59	1.25	22.0	26.9	66.7	-0.99
**Glyma.03G083500**	2.51	2.71	0.31	23.2	22.5	63.6	-0.99
**Glyma.03G084600**	4.88	3.76	0.18	6.3	21.9	37.5	-1.00
**Glyma.03G086100**	6.92	6.03	3.57	16.7	38.2	56.7	-1.00
**Glyma.03G088900**	4.30	3.44	0.99	3.6	18.0	45.5	-0.99
**Glyma.03G091200**	3.41	3.19	1.90	12.5	25.6	90.0	-1.00
**Glyma.03G092700**	5.35	3.79	2.18	16.4	37.8	55.0	-0.98
**Glyma.03G102700**	3.26	2.60	1.47	18.1	35.4	58.3	-0.99
**Glyma.03G104700**	2.96	0.64	0.05	2.5	35.0	40.0	-1.00
**Glyma.03G104800**	1.37	0.12	0	21.4	45.0	66.7	-0.90
**Glyma.03G105300**	5.19	4.67	4.06	13.1	46.5	63.0	-0.99
**Glyma.03G107700**	3.24	3.02	4.82	34.7	20.9	0	-0.89
**Glyma.03G108500**	1.23	1.25	0.11	13.5	15.1	43.8	-1.00
**Glyma.03G109500**	7.68	6.37	5.02	6.9	21.9	38.5	-0.96
**Glyma.03G112100**	4.43	4.98	0.36	12.9	0	72.0	-0.99
**Glyma.03G115300**	2.51	2.70	0.70	43.9	33.3	73.3	-0.99
**Glyma.03G117800**	4.88E-03	4.83E-03	1.66	76.0	96.0	44.0	-0.92
**Glyma.03G119300**	2.73	2.99	1.13	5.4	1.8	35.7	-0.99
**Glyma.03G123100**	2.26	1.82	0.05	29.8	32.3	73.9	-0.96
**Glyma.03G123200**	5.31	5.21	4.14	40.4	50.0	71.4	-0.98
**Glyma.03G125200**	5.18	4.41	0.08	50.5	67.4	90.9	-1.00
**Glyma.03G126100**	1.19	0.44	0.10	8.3	33.8	48.0	-0.99
**Glyma.03G131600**	2.30	1.24	0.06	16.7	44.1	57.1	-1.00
**Glyma.03G132700**	3.61	1.81	0.24	52.3	84.1	86.4	-0.99
**Glyma.03G134100**	3.01	2.37	0.33	55.3	72.0	90.0	-1.00

**Glyma.03G136300**	1.93	1.91	0.29	11.1	28.6	50.0	-0.90
**Glyma.03G137300**	2.82	2.02	0.10	47.5	75.0	79.0	-0.92
**Glyma.03G140600**	1.09	1.64	5.05	86.8	76.3	18.2	-0.99
**Glyma.03G140700**	3.65	2.62	0.04	44.8	64.6	82.8	-1.00
**Glyma.03G141800**	3.03	1.01	0.02	37.8	61.7	76.5	-0.97
**Glyma.03G141900**	3.25	3.74	0.05	16.7	3.3	57.1	-1.00
**Glyma.03G143200**	3.19	3.20	1.60	23.0	23.8	72.4	-1.00
**Glyma.03G154200**	2.34	0.78	0.04	24.8	50.0	72.0	-0.95
**Glyma.03G154300**	2.04	0.11	0.06	21.0	47.5	56.7	-0.97
**Glyma.03G154600**	2.51	1.89	0.40	25.5	55.0	88.2	-1.00
**Glyma.03G154800**	4.40	3.92	5.04	55.6	70.2	33.3	-1.00
**Glyma.03G155700**	5.24	5.10	2.95	36.3	55.2	88.2	-0.97
**Glyma.03G156600**	2.00	1.09	0.27	52.6	86.8	94.4	-0.99
**Glyma.03G159400**	1.53	1.51	9.11E-04	2.3	0	45.5	-1.00
**Glyma.03G166000**	3.85	1.37	0.04	37.0	76.2	92.9	-0.98
**Glyma.03G166800**	3.68	2.29	0.06	27.8	41.5	58.3	-0.97
**Glyma.03G169200**	2.43	1.04	0.01	19.4	66.0	80.0	-1.00
**Glyma.03G169500**	1.42	1.29	0.14	0	0	56.3	-0.99
**Glyma.03G173900**	2.32	1.72	0.25	18.0	36.0	52.0	-1.00
**Glyma.03G177100**	1.79	1.04	0.78	29.9	76.3	84.2	-1.00
**Glyma.03G181800**	3.60	4.04	0.99	0	0	34.6	-0.96
**Glyma.03G191700**	3.16	2.72	0.20	0	0	64.0	-0.96
**Glyma.03G195600**	2.75	1.27	0.07	44.0	85.7	84.0	-0.96
**Glyma.03G197600**	8.24	7.15	6.66	6.7	36.7	26.7	-0.86
**Glyma.03G200700**	1.39	1.19	2.55	59.1	57.1	26.7	-0.99
**Glyma.03G226200**	2.74	2.21	0.26	0	2.5	31.8	-0.95
**Glyma.03G232000**	4.15	2.35	0.09	29.0	53.2	67.7	-0.99
**Glyma.03G236600**	2.30	1.09	1.46	22.2	66.7	66.7	-0.98
**Glyma.03G238700**	6.87	6.56	4.50	54.9	55.1	90.9	-0.97
**Glyma.03G255700**	2.74	0.56	0	32.2	71.6	55.0	-0.87
**Glyma.03G257800**	1.19	1.21	2.34	31.8	42.9	9.5	-0.94
**Glyma.04G002000**	2.60	2.15	4.19	48.1	59.6	23.1	-0.98
**Glyma.04G003200**	8.45	7.56	4.04	59.7	83.9	96.8	-0.98
**Glyma.04G005900**	3.11	2.34	0.73	0	0	30.0	-0.86
**Glyma.04G007200**	1.17	0.84	0.05	19.2	32.7	53.9	-1.00
**Glyma.04G009100**	1.57	2.00	0.17	43.1	49.1	73.7	-0.85
**Glyma.04G010300**	2.57	3.00	0.23	32.6	15.8	52.4	-0.98
**Glyma.04G013900**	2.86	2.60	0.02	3.8	8.6	76.2	-0.99
**Glyma.04G017800**	2.82	0.69	0.08	45.5	70.5	77.3	-0.99
**Glyma.04G018500**	3.68	3.51	2.19	26.3	37.0	63.2	-0.99
**Glyma.04G023300**	2.77	2.20	0.59	0	0	45.8	-0.91
**Glyma.04G023900**	5.07	3.87	0.15	47.8	75.9	81.5	-0.96
**Glyma.04G024300**	5.73	5.21	1.36	34.0	56.0	80.0	-0.98

**Glyma.04G025800**	2.55	1.62	1.43	61.8	91.2	94.1	-1.00
**Glyma.04G026000**	3.09	2.68	1.01	0	0	30.4	-0.95
**Glyma.04G044000**	0.94	1.36	2.30	72.6	88.1	42.9	-0.85
**Glyma.04G051900**	1.70	2.70	4.47	74.5	74.4	30.0	-0.99
**Glyma.04G060000**	5.52	5.05	4.23	28.6	38.1	60.0	-0.98
**Glyma.04G062200**	2.59	2.70	3.69	69.2	59.6	38.5	-0.97
**Glyma.04G063500**	2.33	1.22	4.45E-03	59.0	89.7	93.1	-0.97
**Glyma.04G068700**	1.91	1.38	2.88	33.3	38.1	4.8	-0.99
**Glyma.04G069300**	5.73	4.72	4.13	61.5	79.5	92.3	-0.98
**Glyma.04G070500**	3.08	2.11	0.34	31.3	87.5	91.7	-0.93
**Glyma.04G071700**	3.32	2.22	1.03	19.6	29.2	50.0	-0.92
**Glyma.04G076300**	3.39	2.66	0.26	7.1	34.2	47.4	-0.97
**Glyma.04G080700**	2.83	3.53	4.60	37.5	39.6	4.6	-0.95
**Glyma.04G082800**	2.12	1.69	0.05	16.9	19.7	75.9	-0.96
**Glyma.04G085200**	2.46	2.31	0.39	0	0	42.3	-0.99
**Glyma.04G087700**	3.29	2.67	0.18	50.0	58.0	87.5	-0.98
**Glyma.04G088500**	6.27	4.40	0.42	26.1	37.0	68.8	-0.86
**Glyma.04G089700**	4.44	2.50	0.13	2.3	13.6	36.4	-0.88
**Glyma.04G089900**	5.28	4.45	2.63	10.1	40.4	61.5	-1.00
**Glyma.04G090300**	1.70	0.49	0.05	4.2	21.9	37.5	-0.95
**Glyma.04G091500**	4.29	3.54	1.26	30.8	28.7	62.5	-0.86
**Glyma.04G091800**	7.99	5.85	0.66	16.7	48.0	57.1	-1.00
**Glyma.04G092600**	4.72	4.40	2.38	20.0	35.4	54.2	-0.97
**Glyma.04G094600**	4.40	3.82	2.72	4.7	19.5	41.2	-1.00
**Glyma.04G096100**	2.46	2.87	3.89	84.5	73.3	38.5	-1.00
**Glyma.04G100400**	2.94	1.23	0.22	29.1	75.2	86.7	-1.00
**Glyma.04G108700**	1.76	2.16	2.81	35.4	25.0	0	-1.00
**Glyma.04G109900**	2.97	1.21	0.10	2.9	47.2	53.3	-1.00
**Glyma.04G110000**	6.60	4.50	0.32	21.1	38.1	60.6	-0.92
**Glyma.04G111500**	7.28	6.29	6.07	7.5	32.9	40.0	-1.00
**Glyma.04G112500**	3.44	3.16	1.17	2.9	37.6	71.4	-0.95
**Glyma.04G114800**	2.84	2.80	1.79	4.2	4.5	40.0	-1.00
**Glyma.04G118700**	2.81	1.90	0.47	9.2	42.9	50.0	-0.97
**Glyma.04G120600**	2.22	1.62	0.51	6.7	11.5	75.0	-0.90
**Glyma.04G121900**	1.10	0.79	0	4.4	17.0	39.1	-1.00
**Glyma.04G122900**	5.57	4.43	1.50	21.0	53.6	66.7	-0.99
**Glyma.04G123100**	2.18	2.41	3.42	76.9	72.7	30.8	-1.00
**Glyma.04G123900**	2.38	1.82	1.01	19.8	67.5	100.0	-1.00
**Glyma.04G124100**	10.49	6.54	1.93	18.5	53.7	40.7	-0.91
**Glyma.04G125500**	3.30	3.13	1.96	11.4	26.7	54.2	-0.98
**Glyma.04G125800**	3.90	3.12	2.67	12.5	22.0	48.2	-0.87
**Glyma.04G126100**	2.15	1.74	0.86	7.8	26.6	46.9	-1.00
**Glyma.04G133100**	2.88	3.00	5.65	32.1	65.6	0	-0.85

**Glyma.04G133900**	2.47	2.36	3.78	50.8	52.4	15.4	-1.00
**Glyma.04G139800**	6.77	2.71	0.24	31.7	50.4	66.7	-0.90
**Glyma.04G141900**	2.05	1.73	0.55	11.4	19.8	46.7	-1.00
**Glyma.04G146100**	2.84	2.20	0.06	50.8	69.1	85.7	-0.99
**Glyma.04G147100**	4.68	4.18	1.28	28.3	46.2	65.2	-0.98
**Glyma.04G147600**	2.47	1.09	0.23	2.8	33.3	42.9	-1.00
**Glyma.04G151600**	1.06	4.97	7.15	43.8	31.6	10.5	-0.99
**Glyma.04G156200**	0.90	0.26	1.75	50.6	73.6	14.3	-1.00
**Glyma.04G158400**	1.24	0.65	0.02	8.6	34.5	51.7	-1.00
**Glyma.04G159600**	1.47	0.79	0.02	5.0	33.9	43.3	-0.98
**Glyma.04G160100**	1.92	0.83	0.01	0	20.6	52.4	-0.93
**Glyma.04G161000**	1.34	0.78	0	40.0	65.6	77.8	-0.99
**Glyma.04G162700**	1.70	1.60	0.35	6.3	20.5	41.9	-0.95
**Glyma.04G166900**	1.37	2.28	0.18	24.3	19.1	52.4	-0.88
**Glyma.04G173200**	2.11	0.48	0.06	38.1	75.5	81.3	-1.00
**Glyma.04G174800**	2.85	2.58	0.51	0	0	33.3	-0.98
**Glyma.04G177600**	2.11	1.51	0.45	22.4	45.0	73.1	-1.00
**Glyma.04G178100**	5.59	5.27	1.88	13.0	17.9	60.0	-0.99
**Glyma.04G178300**	1.43	0.57	2.27E-03	37.5	57.5	68.8	-1.00
**Glyma.04G178400**	2.29	0.69	0.40	35.8	93.8	93.8	-1.00
**Glyma.04G179900**	2.33	0.93	0.20	26.6	57.5	61.3	-1.00
**Glyma.04G180200**	2.41	2.11	1.40	17.5	57.8	79.0	-0.95
**Glyma.04G182300**	1.76	1.03	0.32	54.2	79.2	100.0	-1.00
**Glyma.04G184000**	1.50	1.18	0.09	24.2	29.0	58.1	-0.98
**Glyma.04G184400**	2.37	2.21	0.06	22.7	26.2	55.6	-1.00
**Glyma.04G185000**	1.95	0.38	0.01	5.8	23.3	39.1	-0.92
**Glyma.04G185700**	3.86	4.40	6.22	47.4	60.0	30.0	-0.86
**Glyma.04G187800**	2.79	2.12	1.62	2.6	32.1	46.2	-1.00
**Glyma.04G191700**	1.17	0.37	0.01	18.5	39.9	50.0	-1.00
**Glyma.04G192100**	3.33	3.42	2.41	7.3	6.7	38.5	-1.00
**Glyma.04G193900**	0.39	1.12	3.81	40.7	67.6	7.4	-0.87
**Glyma.04G196500**	2.30	2.03	0.28	0	38.2	100.0	-0.99
**Glyma.04G197900**	4.05	3.42	2.86	10.8	43.6	66.7	-1.00
**Glyma.04G199300**	3.14	2.88	1.02	0	0	54.6	-0.98
**Glyma.04G199500**	2.76	2.96	1.54	65.4	57.7	96.2	-1.00
**Glyma.04G203600**	2.10	1.37	0.79	0	56.6	54.6	-0.94
**Glyma.04G205300**	5.58	4.26	1.68	44.4	73.3	94.4	-1.00
**Glyma.04G214000**	4.84	3.14	0.05	25.0	59.8	83.3	-0.99
**Glyma.04G216200**	4.18	3.99	0.27	40.0	35.0	67.5	-0.97
**Glyma.04G217400**	1.99E-06	1.93E-06	3.89	49.8	60.5	21.1	-0.97
**Glyma.04G218500**	3.65	3.10	2.24	0	2.6	31.3	-0.90
**Glyma.04G219500**	3.35	2.58	2.29	3.1	17.7	35.7	-0.92
**Glyma.04G219900**	1.21	1.26	0	0	0	52.0	-1.00

**Glyma.04G220100**	1.37	1.50	2.45	22.6	39.9	0	-0.87
**Glyma.04G223400**	8.63	7.85	7.40	11.7	46.7	44.4	-0.95
**Glyma.04G226900**	8.04	7.03	7.69	16.4	66.7	52.2	-0.95
**Glyma.04G231000**	1.93	2.00	0.84	0	0	37.5	-1.00
**Glyma.04G232000**	2.19	1.92	0.51	0	0	30.4	-0.97
**Glyma.04G235500**	3.24	2.44	0.59	36.1	80.8	90.9	-0.94
**Glyma.04G239800**	2.81	1.78	0.49	16.1	34.4	59.3	-0.97
**Glyma.04G240000**	2.58	1.74	0.04	5.0	7.1	63.6	-0.86
**Glyma.04G240700**	1.67	1.66	0.04	13.8	19.6	54.6	-0.99
**Glyma.04G241600**	2.50	0.62	0	13.2	35.2	43.8	-0.99
**Glyma.04G241800**	4.58	4.62	0.48	67.8	60.4	100.0	-0.99
**Glyma.04G243800**	3.02	2.27	0.09	18.2	36.1	53.3	-1.00
**Glyma.04G251300**	3.99	3.60	1.97	6.3	27.1	66.7	-1.00
**Glyma.04G255500**	3.35	2.40	0.03	28.5	68.8	100.0	-1.00
**Glyma.04G256600**	3.49	3.08	2.21	9.3	8.3	60.9	-0.90
**Glyma.04G256900**	2.04	1.35	0.75	33.9	62.5	85.7	-0.99
**Glyma.05G001200**	4.09	4.75	5.90	47.1	51.4	9.5	-0.95
**Glyma.05G003600**	1.50	1.22	0.48	3.7	13.5	40.0	-1.00
**Glyma.05G006600**	0.24	1.15	3.37	79.7	96.4	46.7	-0.90
**Glyma.05G007200**	2.58	1.92	1.07	9.8	36.1	50.0	-0.99
**Glyma.05G011000**	0.87	2.08	1.17	53.8	23.1	50.0	-1.00
**Glyma.05G011200**	7.00	3.72	0.76	25.9	46.4	60.7	-0.94
**Glyma.05G011600**	3.42	3.23	0.26	0	3.8	35.7	-1.00
**Glyma.05G014000**	2.22	2.02	0.86	0	11.1	41.7	-1.00
**Glyma.05G018200**	2.81	2.61	0.97	41.9	39.5	73.7	-0.97
**Glyma.05G023100**	1.53	0.93	0.19	31.0	51.9	76.2	-0.99
**Glyma.05G025200**	0.08	0.26	2.57	60.7	78.3	20.0	-0.95
**Glyma.05G027300**	3.25	3.02	1.91	0	0	65.8	-0.97
**Glyma.05G030100**	3.21	2.05	0.08	30.6	78.6	100.0	-1.00
**Glyma.05G034200**	2.30	2.19	0.91	0	10.0	58.3	-1.00
**Glyma.05G039800**	2.56	3.25	0.55	0	0	36.4	-0.90
**Glyma.05G039900**	2.42	1.95	0.51	32.5	61.7	90.0	-0.99
**Glyma.05G041400**	1.20	0.33	0.10	2.5	35.0	55.0	-0.97
**Glyma.05G042000**	1.47	1.77	0.01	83.0	46.9	95.0	-0.86
**Glyma.05G042800**	0.19	1.45	6.16	49.1	36.4	7.7	-0.96
**Glyma.05G043200**	5.66	4.46	5.74	37.1	64.3	28.6	-0.99
**Glyma.05G046100**	3.70	2.97	0.80	5.3	26.3	61.1	-1.00
**Glyma.05G047300**	2.22	2.03	0.41	6.7	11.9	50.0	-1.00
**Glyma.05G049400**	1.44	2.20	2.61	61.3	37.5	30.0	-0.97
**Glyma.05G050600**	1.01	1.76	0.40	6.5	0	38.5	-0.85
**Glyma.05G051600**	4.10	4.08	5.32	47.3	40.0	7.1	-0.98
**Glyma.05G053000**	3.43	3.81	6.05	77.3	56.8	22.7	-0.95
**Glyma.05G056100**	4.54	2.64	0.35	34.8	68.7	65.2	-0.95

**Glyma.05G056400**	4.67	3.15	1.20	35.4	65.2	83.3	-0.99
**Glyma.05G059100**	3.58	3.73	5.08	67.9	72.6	6.9	-0.99
**Glyma.05G062800**	0.15	1.67	3.52	37.0	34.8	4.4	-0.99
**Glyma.05G064600**	0.24	0.78	2.02	38.5	35.6	7.7	-1.00
**Glyma.05G065900**	3.19	2.42	2.18	14.3	33.7	57.9	-0.91
**Glyma.05G066500**	3.96	3.18	1.26	12.0	24.3	57.1	-0.97
**Glyma.05G068800**	3.49	0.87	0.01	0	39.0	31.8	-0.97
**Glyma.05G070200**	2.40	1.26	5.02E-04	58.0	81.3	100.0	-0.99
**Glyma.05G072200**	2.58	0.72	0.40	12.1	41.4	55.2	-0.97
**Glyma.05G075100**	4.31	3.27	0.36	3.7	27.8	46.2	-1.00
**Glyma.05G076800**	2.59	1.96	0	10.0	62.5	78.6	-0.93
**Glyma.05G079700**	5.17	4.35	1.49	14.3	30.4	60.7	-0.99
**Glyma.05G079800**	1.66	1.03	0.27	0	25.7	38.1	-0.99
**Glyma.05G080800**	3.13	2.67	4.19	70.0	70.0	35.0	-0.98
**Glyma.05G086900**	2.56	3.17	1.45	1.6	3.2	41.9	-0.85
**Glyma.05G088700**	2.18	2.43	0.91	8.2	9.6	38.5	-0.96
**Glyma.05G090900**	4.32	4.00	0.35	11.8	14.6	54.2	-0.99
**Glyma.05G091100**	2.97	2.61	1.70	2.1	21.1	42.9	-0.99
**Glyma.05G091600**	1.42	0	1.97	15.0	43.5	13.3	-0.92
**Glyma.05G091700**	2.92	2.75	0.47	31.6	40.4	68.2	-0.99
**Glyma.05G091800**	1.09	0.96	0.01	14.9	18.9	46.0	-1.00
**Glyma.05G095600**	2.47	4.32	4.90	56.5	27.9	8.3	-1.00
**Glyma.05G095900**	0.42	1.30	3.26	48.3	47.7	12.5	-0.99
**Glyma.05G097300**	3.67	2.57	2.58	28.8	61.5	69.2	-0.98
**Glyma.05G099500**	5.90	5.67	4.70	31.3	45.8	70.8	-0.99
**Glyma.05G101700**	3.63	2.30	0.07	18.1	37.0	52.2	-0.99
**Glyma.05G102100**	5.34	3.17	0.28	48.1	88.4	74.1	-0.85
**Glyma.05G103600**	1.91	0.40	0	4.0	51.9	63.0	-1.00
**Glyma.05G105400**	6.92	5.84	2.20	4.8	21.2	46.2	-0.98
**Glyma.05G105800**	3.56	2.75	1.05	10.0	26.7	46.7	-1.00
**Glyma.05G106100**	4.48	3.61	2.11	3.9	36.0	50.0	-0.99
**Glyma.05G108600**	1.89	1.01	0	34.3	44.4	84.2	-0.89
**Glyma.05G110200**	8.72	8.19	3.17	5.8	11.6	73.1	-0.97
**Glyma.05G111800**	2.62	2.17	0.74	16.0	47.0	88.0	-1.00
**Glyma.05G113700**	5.05	5.00	2.99	42.9	48.2	75.0	-0.99
**Glyma.05G115100**	3.33	3.53	4.41	41.7	26.7	10.0	-0.93
**Glyma.05G119000**	3.92	3.10	0.11	5.6	16.5	72.2	-0.95
**Glyma.05G119100**	3.44	1.85	1.42	36.1	70.8	72.2	-1.00
**Glyma.05G122900**	1.42	1.81	2.78	87.1	87.5	41.7	-0.98
**Glyma.05G126800**	0.04	0.73	2.01	40.9	54.5	13.6	-0.86
**Glyma.05G127200**	4.86	4.70	3.08	0	0	30.8	-0.99
**Glyma.05G128700**	1.66	1.23	0.03	15.0	38.3	68.4	-1.00
**Glyma.05G129300**	2.98	3.07	1.58	2.1	0	36.8	-1.00

**Glyma.05G133100**	3.61	3.70	4.97	74.0	78.0	40.7	-0.99
**Glyma.05G134100**	2.84	2.37	1.63	0	0	32.1	-0.87
**Glyma.05G136600**	1.24	0.68	0	8.8	32.4	45.5	-0.99
**Glyma.05G136900**	1.11	0.78	0	4.5	18.3	42.9	-1.00
**Glyma.05G137100**	1.94	0.63	1.13E-03	1.9	30.8	40.0	-1.00
**Glyma.05G138600**	3.82	3.33	1.78	32.2	44.1	72.4	-1.00
**Glyma.05G139500**	0.05	0.40	3.33	43.3	54.6	12.5	-0.96
**Glyma.05G140300**	3.93	3.71	2.78	27.3	34.6	71.4	-1.00
**Glyma.05G140900**	1.81	1.22	0.03	23.4	46.9	87.1	-0.99
**Glyma.05G141000**	4.23	2.95	1.02	50.5	79.6	100.0	-1.00
**Glyma.05G141900**	2.24	1.94	3.50	62.5	70.8	26.3	-1.00
**Glyma.05G142500**	0.11	0.68	7.30	25.0	37.5	0	-0.94
**Glyma.05G144000**	2.88	2.81	0.87	0	33.9	83.3	-0.94
**Glyma.05G144900**	2.29	1.18	1.65	17.9	55.0	64.3	-0.87
**Glyma.05G145700**	2.01	0.74	4.44E-06	2.1	22.5	45.8	-0.94
**Glyma.05G148900**	2.72	1.84	0.17	29.5	43.2	65.0	-0.98
**Glyma.05G150200**	1.35	0.99	0.12	0	4.2	33.3	-0.96
**Glyma.05G157300**	7.69	7.53	3.74	15.4	5.6	70.0	-0.97
**Glyma.05G166400**	2.75	1.55	0.34	22.0	38.5	82.4	-0.89
**Glyma.05G169500**	4.04	3.59	2.63	63.6	86.4	95.5	-0.95
**Glyma.05G174700**	2.33	1.70	0.42	47.9	71.4	87.5	-0.99
**Glyma.05G178000**	4.88	3.87	5.38	15.4	38.3	7.7	-0.98
**Glyma.05G179000**	2.39	2.15	0.59	3.3	26.1	52.9	-0.96
**Glyma.05G181700**	3.32	2.68	1.76	32.3	57.4	70.4	-0.99
**Glyma.05G197700**	3.21	3.02	1.81	40.3	37.6	67.7	-0.96
**Glyma.05G198300**	3.64	2.77	2.59	32.9	67.2	72.2	-1.00
**Glyma.05G207300**	2.15	1.23	0.61	1.7	17.2	36.4	-0.95
**Glyma.05G208400**	2.65	2.12	3.15	47.3	55.8	15.4	-0.97
**Glyma.05G208700**	6.43	3.04	0.25	29.9	84.7	84.6	-1.00
**Glyma.05G212800**	1.07	0.34	0	9.4	40.6	50.0	-1.00
**Glyma.05G216400**	1.28	1.11	0.03	0	0	33.3	-0.98
**Glyma.05G218700**	1.04	1.06	0.04	0	0	46.7	-1.00
**Glyma.05G219400**	7.25	6.38	5.38	8.3	22.9	60.0	-0.93
**Glyma.05G219600**	4.44	2.79	3.05	47.7	70.5	81.8	-0.91
**Glyma.05G220600**	2.82	2.77	1.26	23.9	43.5	65.2	-0.90
**Glyma.05G229300**	4.07	3.52	0.21	4.2	37.5	63.6	-0.97
**Glyma.05G231200**	2.43	2.14	0.06	0	0	60.0	-0.98
**Glyma.05G231900**	1.93	1.83	0.16	0	0	30.8	-1.00
**Glyma.05G232000**	1.62	1.92	0.64	0	0	50.0	-0.95
**Glyma.05G236600**	2.87	2.15	0.03	50.0	60.5	94.7	-0.97
**Glyma.05G237000**	4.45	3.71	0.46	0	0	42.9	-0.91
**Glyma.05G239400**	3.02	2.62	1.02	12.7	21.4	61.9	-0.99
**Glyma.05G241900**	2.93	1.63	0.52	0	52.6	83.3	-0.99

**Glyma.05G243400**	2.72	2.35	3.79	46.4	82.6	25.0	-0.87
**Glyma.05G245900**	1.60	1.70	1.27E-03	0	0	30.8	-1.00
**Glyma.05G246900**	2.58	2.06	1.19	23.3	46.0	66.7	-1.00
**Glyma.05G247300**	4.30	3.54	0.16	21.7	26.9	53.9	-0.96
**Glyma.06G001100**	1.41	0.97	2.34	82.6	89.9	58.1	-1.00
**Glyma.06G001700**	2.85	2.39	0.37	3.5	24.1	46.7	-0.99
**Glyma.06G005000**	2.15	0.89	0.05	34.9	50.0	72.2	-0.92
**Glyma.06G005600**	2.32	2.44	1.14	0	0	33.3	-0.99
**Glyma.06G005900**	1.78	1.06	0.10	8.3	43.1	63.9	-1.00
**Glyma.06G010400**	1.79	0.52	0.90	0	48.7	57.1	-0.94
**Glyma.06G010600**	2.20	1.52	0.85	51.9	94.4	92.6	-0.91
**Glyma.06G012600**	4.16	4.23	2.46	0	0	33.3	-1.00
**Glyma.06G012700**	1.56	1.71	0.52	6.3	14.7	38.5	-0.91
**Glyma.06G013100**	3.71	3.08	2.05	0	0	50.0	-0.85
**Glyma.06G013600**	2.33	1.44	0.12	0	58.7	62.5	-0.93
**Glyma.06G027100**	1.73	2.03	3.43	43.7	23.5	0	-0.93
**Glyma.06G031700**	4.09	4.14	2.17	45.5	44.0	77.3	-1.00
**Glyma.06G034200**	3.32	2.15	0.10	62.7	76.4	100.0	-0.96
**Glyma.06G034900**	2.36	1.48	1.35	34.7	94.1	81.3	-0.96
**Glyma.06G039800**	2.50	0.88	0.08	11.5	38.5	42.3	-1.00
**Glyma.06G042900**	5.17	6.02	1.75	0	0	31.3	-0.88
**Glyma.06G045500**	2.67	1.87	0.58	29.7	59.4	68.8	-0.97
**Glyma.06G052400**	1.67	1.87	0.34	0	8.3	30.4	-0.89
**Glyma.06G052600**	2.61	2.22	1.16	7.7	38.8	52.0	-0.93
**Glyma.06G052800**	3.70	0.98	0.02	0	47.1	29.2	-0.90
**Glyma.06G058600**	1.12	0.98	3.59	76.2	80.0	45.0	-1.00
**Glyma.06G058800**	2.34	0.91	0.40	41.7	85.0	100.0	-0.99
**Glyma.06G063500**	2.14	1.56	0.75	12.5	16.7	62.5	-0.88
**Glyma.06G066200**	1.41	0.70	9.81E-06	28.1	84.1	77.8	-0.88
**Glyma.06G067900**	0.49	0.40	3.72	77.8	80.6	44.4	-1.00
**Glyma.06G068400**	4.96	4.99	3.15	0	0	31.4	-1.00
**Glyma.06G070400**	2.13	2.53	3.99	31.7	25.6	0	-1.00
**Glyma.06G076400**	3.14	3.57	4.35	46.0	48.4	16.7	-0.95
**Glyma.06G077200**	4.61	4.44	5.98	43.3	66.3	5.9	-0.95
**Glyma.06G089600**	3.80	3.00	0.08	0	0	38.9	-0.89
**Glyma.06G090800**	0.72	2.15	4.56	58.2	45.5	27.3	-0.95
**Glyma.06G090900**	0.23	0.64	3.27	23.5	38.2	0	-0.91
**Glyma.06G092200**	2.24	2.39	1.07	13.3	30.0	83.3	-0.93
**Glyma.06G093600**	5.48	4.53	0.17	0	0	33.3	-0.87
**Glyma.06G096700**	4.92	4.36	3.07	50.0	72.0	80.0	-0.95
**Glyma.06G098600**	3.58	2.80	1.93	0	9.1	38.5	-0.91
**Glyma.06G102400**	2.04	1.02	0.17	54.5	86.4	100.0	-1.00
**Glyma.06G114500**	0.92	0.76	2.79	40.8	44.7	8.3	-1.00

**Glyma.06G116100**	3.02	0.79	0.01	42.5	86.5	78.6	-0.97
**Glyma.06G119500**	4.59	4.03	2.38	0	0	30.8	-0.91
**Glyma.06G121900**	1.86	0.91	0.36	43.3	78.5	86.7	-1.00
**Glyma.06G122900**	0.25	0.58	1.81	68.9	79.6	48.2	-0.90
**Glyma.06G124500**	3.18	3.61	1.22	0	3.3	31.6	-0.91
**Glyma.06G125500**	2.79	0.73	0.01	27.3	60.0	63.6	-1.00
**Glyma.06G127200**	3.10	3.09	0.56	27.6	22.7	59.3	-0.99
**Glyma.06G127400**	2.62	2.72	0.10	0	0	69.2	-1.00
**Glyma.06G134300**	2.64	2.28	3.53	39.7	59.6	20.0	-0.95
**Glyma.06G137100**	5.90	5.22	0.87	0	0	69.2	-0.92
**Glyma.06G137300**	2.69	2.28	0.16	21.3	36.7	53.3	-0.98
**Glyma.06G139300**	0.72	1.48	0.09	53.5	6.9	73.7	-1.00
**Glyma.06G140900**	5.62	5.99	4.94	27.8	27.8	59.3	-0.90
**Glyma.06G150300**	6.58	5.22	0.52	42.9	71.4	85.7	-1.00
**Glyma.06G151500**	1.75	1.14	0.05	8.3	30.6	38.5	-0.96
**Glyma.06G153000**	2.16	1.13	0.03	20.3	62.5	68.8	-0.98
**Glyma.06G160700**	2.84	1.23	0	16.1	44.0	57.7	-0.99
**Glyma.06G166200**	4.22	3.12	1.85	40.0	80.8	80.0	-0.94
**Glyma.06G169600**	4.11	3.70	2.97	18.2	22.4	60.0	-0.93
**Glyma.06G170300**	2.09	2.75	0.12	65.9	47.4	95.8	-1.00
**Glyma.06G176800**	3.10	2.91	0.91	50.0	70.0	87.0	-0.91
**Glyma.06G177900**	4.29	3.93	5.07	45.7	60.0	21.1	-0.99
**Glyma.06G178200**	1.84	1.44	0.25	12.0	27.8	61.3	-1.00
**Glyma.06G181600**	5.00	3.57	2.04	2.3	18.0	33.3	-0.97
**Glyma.06G184900**	2.19	1.86	0.85	37.0	39.1	69.6	-0.96
**Glyma.06G185000**	4.48	3.56	0.17	51.8	75.0	95.5	-1.00
**Glyma.06G186300**	0.18	3.86	6.98	80.4	67.5	38.1	-0.98
**Glyma.06G186900**	3.96	3.15	1.72	10.0	27.3	50.0	-0.99
**Glyma.06G191000**	3.06	1.42	0.05	7.4	16.7	40.0	-0.85
**Glyma.06G191900**	2.63	2.41	1.53	10.9	23.9	47.8	-0.99
**Glyma.06G195600**	1.63	2.18	0.01	18.4	12.5	43.8	-0.97
**Glyma.06G196800**	1.28	0.84	3.57E-04	13.1	31.3	43.8	-0.99
**Glyma.06G198000**	1.77	1.32	0.48	19.6	37.5	58.3	-1.00
**Glyma.06G201700**	2.99	3.18	4.27	46.2	53.8	19.2	-0.95
**Glyma.06G205400**	3.02	2.89	0.10	6.9	15.3	47.1	-0.99
**Glyma.06G206400**	1.77	2.23	0.55	15.3	0	33.3	-1.00
**Glyma.06G206600**	1.59	1.78	0.30	1.7	3.3	46.7	-0.98
**Glyma.06G211600**	3.00	1.29	0.04	25.0	54.2	63.2	-1.00
**Glyma.06G219600**	1.60	1.07	0.48	7.8	24.1	44.4	-0.99
**Glyma.06G219700**	3.42	2.49	0.76	39.7	63.6	73.9	-0.99
**Glyma.06G223800**	3.05	2.73	1.76	28.1	41.5	64.5	-1.00
**Glyma.06G227200**	7.53	6.21	3.27	17.4	39.4	54.6	-1.00
**Glyma.06G228800**	2.81	2.47	0.31	31.3	37.0	67.9	-0.99

**Glyma.06G230400**	4.01	3.76	1.50	0	19.4	50.0	-0.98
**Glyma.06G231500**	5.77	4.86	3.31	32.1	50.0	64.3	-1.00
**Glyma.06G235000**	3.30	1.52	0	0	34.5	28.6	-0.94
**Glyma.06G235900**	2.48	1.41	0.05	23.7	56.9	78.6	-1.00
**Glyma.06G239300**	2.16	1.87	0.51	8.2	26.3	52.0	-0.99
**Glyma.06G239500**	2.33	1.30	0.36	7.7	38.9	66.7	-0.98
**Glyma.06G240000**	1.90	1.82	2.98	56.7	70.2	7.1	-0.99
**Glyma.06G240400**	1.54	0.31	0.02	7.1	31.0	42.9	-0.98
**Glyma.06G243500**	3.45	3.81	1.24	17.6	23.1	61.5	-0.93
**Glyma.06G246600**	2.43	1.51	0.52	10.0	41.9	55.6	-1.00
**Glyma.06G247200**	2.00	2.11	1.73E-05	51.1	47.1	88.2	-1.00
**Glyma.06G254500**	2.41	1.83	0.31	63.2	84.8	100.0	-0.98
**Glyma.06G261500**	2.09	2.41	0.62	35.7	24.4	60.0	-1.00
**Glyma.06G265800**	3.30	2.38	0.27	50.5	76.3	84.2	-0.97
**Glyma.06G276500**	0.08	1.74	8.13	36.6	19.7	2.9	-0.87
**Glyma.06G277500**	4.07	4.05	2.87	36.8	47.0	73.3	-0.97
**Glyma.06G284700**	1.19	0.81	0.14	11.7	17.7	51.6	-0.95
**Glyma.06G289300**	3.02	3.05	1.96	55.2	36.5	82.4	-0.93
**Glyma.06G290000**	1.49	4.37	6.50	92.6	83.9	44.0	-1.00
**Glyma.06G292200**	0.32	0.82	1.77	50.0	40.4	0	-1.00
**Glyma.06G293200**	3.95	4.43	2.56	0	0	52.6	-0.92
**Glyma.06G294500**	1.35	0.44	0	31.4	75.2	64.3	-0.90
**Glyma.06G296100**	0.12	0.28	2.20	66.0	97.5	20.0	-0.90
**Glyma.06G299500**	1.46	0.51	0.09	7.1	45.2	53.6	-1.00
**Glyma.06G299700**	1.18	0.18	0	37.5	80.0	75.0	-0.98
**Glyma.06G302800**	1.49	0.87	3.41	21.6	34.7	0	-0.96
**Glyma.06G308300**	2.41	3.05	0.74	13.2	0	36.8	-0.99
**Glyma.06G313500**	1.36	1.56	0.02	17.6	2.9	53.9	-1.00
**Glyma.06G315300**	1.74	2.17	3.14	77.4	84.6	26.9	-0.95
**Glyma.06G320200**	4.45	3.90	5.26	21.0	53.5	0	-0.94
**Glyma.06G324300**	5.66	5.12	0.54	8.0	26.0	52.0	-1.00
**Glyma.07G004300**	3.15	3.13	0.17	34.5	36.7	78.6	-1.00
**Glyma.07G004500**	5.50	5.30	1.46	0	0	74.2	-0.99
**Glyma.07G010800**	2.88	2.53	1.23	10.3	18.4	64.3	-0.98
**Glyma.07G013500**	3.37	2.79	3.92E-05	1.8	0	33.3	-0.92
**Glyma.07G014500**	3.69	1.13	0.71	30.6	59.1	65.4	-0.99
**Glyma.07G014600**	1.50	0.07	0.04	33.3	68.3	60.0	-0.97
**Glyma.07G015200**	1.60	0.61	0.17	5.4	30.4	42.9	-0.99
**Glyma.07G018100**	0.17	1.23	0.07	26.6	0	32.1	-0.99
**Glyma.07G018700**	1.30	1.33	0.18	2.4	16.7	41.7	-0.92
**Glyma.07G020000**	8.07	6.97	7.36	9.6	67.7	41.9	-0.98
**Glyma.07G020200**	2.81	2.65	1.28	35.2	55.6	100.0	-0.99
**Glyma.07G023500**	0.65	0.72	7.29	39.6	56.4	18.8	-0.90

**Glyma.07G035900**	3.96	2.54	2.04	27.8	59.0	83.3	-0.95
**Glyma.07G043500**	2.31	2.08	0.15	27.8	50.7	68.8	-0.92
**Glyma.07G048500**	1.44	0.47	0.16	8.9	32.1	42.9	-0.99
**Glyma.07G049100**	3.89	3.96	2.38	0	0	70.4	-1.00
**Glyma.07G049700**	1.88	1.78	0.74	31.8	25.0	70.0	-0.97
**Glyma.07G052200**	0	6.32E-06	2.06	47.2	54.2	22.2	-0.98
**Glyma.07G054700**	3.64	1.72	0.14	54.5	100.0	100.0	-0.98
**Glyma.07G068200**	1.65	1.57	0.48	47.2	65.3	80.6	-0.88
**Glyma.07G068300**	3.84	3.45	0.36	5.9	25.0	37.5	-0.92
**Glyma.07G070600**	3.37	2.85	2.29	25.0	56.7	65.0	-0.97
**Glyma.07G072300**	1.48	0.03	0.27	52.0	84.0	72.0	-0.96
**Glyma.07G075200**	1.81	0.87	0.05	11.3	56.5	70.4	-1.00
**Glyma.07G080400**	2.47	1.59	0.94	31.0	65.5	79.3	-1.00
**Glyma.07G081700**	3.94	3.60	0.02	6.8	20.5	63.6	-1.00
**Glyma.07G082500**	1.44	1.18	0.19	0	15.4	38.9	-0.99
**Glyma.07G082700**	4.12	3.25	1.70	2.4	38.1	41.2	-0.93
**Glyma.07G082800**	2.18	2.33	1.11	2.4	0	35.7	-1.00
**Glyma.07G086400**	1.76	2.25	3.42	43.1	31.7	10.5	-0.98
**Glyma.07G086500**	1.28	1.73	0.45	0	0	33.3	-0.89
**Glyma.07G090000**	1.14	1.42	0.24	0	0	42.1	-0.95
**Glyma.07G090900**	1.96	1.64	0.61	0	0	55.0	-0.95
**Glyma.07G091800**	3.76	4.37	6.87	30.3	45.6	13.3	-0.85
**Glyma.07G096700**	2.55	1.34	0	9.1	75.0	100.0	-1.00
**Glyma.07G102500**	2.54	1.62	0.90	30.4	61.5	75.0	-1.00
**Glyma.07G103100**	1.48	0.81	0	0	5.6	41.2	-0.88
**Glyma.07G103500**	2.58	1.51	0.04	12.7	37.8	48.7	-1.00
**Glyma.07G105900**	4.78	4.37	3.46	0	5.2	50.0	-0.94
**Glyma.07G107800**	2.64	2.33	0.62	5.6	29.9	88.2	-1.00
**Glyma.07G107900**	4.83	5.12	3.08	7.1	7.1	42.9	-0.97
**Glyma.07G110900**	3.43	3.27	2.14	16.5	7.7	40.0	-0.91
**Glyma.07G115900**	8.51	7.16	6.69	11.5	47.7	52.2	-1.00
**Glyma.07G117500**	2.84	2.64	4.93E-05	4.8	17.7	58.1	-1.00
**Glyma.07G118800**	2.71	2.27	3.53	23.2	32.9	2.9	-1.00
**Glyma.07G119200**	0.59	0.63	1.70	56.9	47.4	14.3	-0.98
**Glyma.07G122000**	1.28	0.39	2.25	73.7	86.8	52.6	-1.00
**Glyma.07G124600**	1.59	0.31	0.20	7.1	37.9	25.0	-0.89
**Glyma.07G125900**	3.46	3.39	0.93	7.1	0	72.7	-0.99
**Glyma.07G126300**	4.79	4.48	3.59	60.0	76.0	91.3	-0.98
**Glyma.07G129700**	3.26	2.82	0.70	0	0	50.0	-0.95
**Glyma.07G129900**	2.18	2.55	0.01	1.5	0	36.4	-0.97
**Glyma.07G131200**	1.18	0.71	0.05	6.9	27.4	45.8	-1.00
**Glyma.07G132100**	1.50	0.27	0.02	4.7	25.0	35.0	-0.97
**Glyma.07G132900**	2.96	2.26	1.81	18.5	43.5	66.7	-0.98

**Glyma.07G133800**	2.11	1.39	0.03	41.1	66.4	78.6	-0.98
**Glyma.07G137100**	3.05	2.05	1.23	9.6	33.3	44.4	-1.00
**Glyma.07G138400**	4.13	3.93	0.95	14.3	33.6	55.0	-0.94
**Glyma.07G143100**	3.61	3.94	4.86	56.4	57.7	9.1	-0.98
**Glyma.07G144100**	1.88	0.38	4.60E-06	12.5	37.3	45.5	-0.99
**Glyma.07G144200**	1.31	0.67	0.01	28.6	51.8	71.4	-1.00
**Glyma.07G148700**	1.71	0.47	1.30E-06	2.6	32.0	41.7	-1.00
**Glyma.07G149400**	1.98	0.27	0.03	61.9	97.6	89.5	-0.96
**Glyma.07G150900**	6.39	2.75	0.39	6.2	36.1	40.0	-1.00
**Glyma.07G152600**	6.26E-05	0.45	1.69	25.1	32.1	0	-0.94
**Glyma.07G157900**	1.90	2.06	4.15	34.6	40.6	4.6	-0.98
**Glyma.07G159700**	2.61	0.57	0.01	17.8	56.7	86.7	-0.93
**Glyma.07G160400**	0.51	0.44	1.51	75.0	78.3	24.0	-1.00
**Glyma.07G160500**	6.15	4.88	2.35	19.2	38.9	80.0	-0.94
**Glyma.07G163600**	3.05	2.10	0.05	16.1	33.0	50.0	-1.00
**Glyma.07G165000**	3.18	1.75	0.77	15.8	50.7	41.7	-0.89
**Glyma.07G170000**	1.79	1.67	0.17	7.7	0	45.5	-0.96
**Glyma.07G170700**	4.19	3.60	2.23	0	21.5	57.1	-1.00
**Glyma.07G172700**	1.48	1.14	0.38	1.6	9.7	33.3	-0.99
**Glyma.07G173100**	1.88	1.76	0.21	0	10.3	33.3	-0.98
**Glyma.07G173800**	2.88	3.22	5.04	68.6	66.3	35.3	-1.00
**Glyma.07G180000**	3.31	2.92	2.15	15.9	40.0	82.4	-1.00
**Glyma.07G185200**	6.45	5.92	0.67	0	22.0	38.9	-0.96
**Glyma.07G189700**	2.46	1.83	0.78	18.7	18.9	57.9	-0.86
**Glyma.07G195600**	2.94	2.04	0.20	17.2	24.6	65.4	-0.91
**Glyma.07G198400**	2.08	1.97	0.66	0	15.0	30.0	-0.91
**Glyma.07G201100**	1.95	1.18	0.47	57.5	90.0	95.0	-0.97
**Glyma.07G201600**	2.39	1.25	0.03	30.6	70.3	71.9	-0.96
**Glyma.07G201900**	1.94	1.75	0.59	13.1	43.2	61.1	-0.89
**Glyma.07G202900**	2.17	1.88	0.92	6.7	38.1	100.0	-1.00
**Glyma.07G205100**	3.37	2.03	0.01	0	42.3	64.0	-1.00
**Glyma.07G207500**	2.58	2.50	1.40	3.3	11.0	46.7	-1.00
**Glyma.07G208000**	3.11	2.42	0.07	24.9	37.6	58.6	-1.00
**Glyma.07G208100**	1.41	1.11	0	16.9	30.7	79.2	-1.00
**Glyma.07G210800**	7.34	7.65	5.36	14.6	16.7	50.0	-0.96
**Glyma.07G217900**	1.30	1.13	0.15	6.3	16.7	50.0	-1.00
**Glyma.07G219600**	1.44	0.12	0	23.1	37.5	53.9	-0.87
**Glyma.07G221300**	5.12	3.96	0.74	22.0	37.1	72.0	-0.95
**Glyma.07G222800**	2.84	2.60	1.41	13.5	23.7	75.0	-1.00
**Glyma.07G225500**	1.30	1.05	2.36	36.8	50.0	18.2	-0.95
**Glyma.07G225800**	1.95	1.48	0.01	50.0	57.1	95.2	-0.97
**Glyma.07G227600**	1.95	0.19	0.05	25.8	88.1	71.4	-0.96
**Glyma.07G228100**	4.33	3.52	2.16	38.5	58.9	69.6	-0.99

**Glyma.07G228400**	2.27	1.82	4.98	48.1	41.7	16.7	-0.97
**Glyma.07G229200**	1.35	0.38	0.03	32.5	60.0	70.0	-1.00
**Glyma.07G238900**	4.02	3.71	0.99	0	4.5	36.4	-1.00
**Glyma.07G243000**	1.69	0.80	0.48	0	25.0	33.3	-1.00
**Glyma.07G254600**	5.66	4.33	0.42	12.5	44.6	64.3	-1.00
**Glyma.07G256500**	3.06	3.03	1.16	0	0	36.8	-1.00
**Glyma.07G260200**	5.28	5.32	4.00	72.8	60.7	92.9	-0.94
**Glyma.07G266400**	2.22	2.12	1.20	18.2	36.4	90.9	-0.99
**Glyma.07G268100**	1.60	1.06	1.13E-05	0	3.3	37.5	-0.92
**Glyma.07G272700**	0.17	0	1.41	58.0	76.0	44.0	-0.86
**Glyma.07G273800**	1.29	1.32	3.37	28.8	47.7	8.7	-0.87
**Glyma.08G002700**	5.72	3.84	3.77	27.3	60.8	59.1	-1.00
**Glyma.08G005200**	4.09	3.70	1.05	15.9	20.0	52.9	-0.99
**Glyma.08G013000**	2.32	2.54	0.99	51.8	49.4	89.3	-0.99
**Glyma.08G014800**	2.36	0.77	3.51E-04	6.6	25.3	38.1	-0.97
**Glyma.08G016100**	3.50	2.78	0.95	35.5	60.9	90.6	-1.00
**Glyma.08G016300**	0.49	0.81	3.01	73.3	73.3	3.3	-1.00
**Glyma.08G016400**	3.25	3.50	4.96	56.5	78.6	13.0	-0.92
**Glyma.08G016600**	4.48	3.34	3.09	0	75.3	61.5	-0.96
**Glyma.08G020600**	2.85	2.81	0.38	58.4	45.1	85.7	-0.94
**Glyma.08G021500**	1.05	1.32	0.03	13.2	21.5	63.6	-0.91
**Glyma.08G028400**	3.24	3.26	4.28	83.2	70.2	43.8	-0.95
**Glyma.08G032700**	5.75	4.95	0.67	0	0	38.5	-0.90
**Glyma.08G033600**	1.73	0.64	0.70	37.2	79.2	85.7	-0.99
**Glyma.08G036400**	5.59	4.80	0.26	2.8	0	46.7	-0.88
**Glyma.08G040000**	3.81	3.20	2.34	30.0	75.0	85.0	-0.95
**Glyma.08G047100**	3.41	3.03	0.91	3.3	20.0	40.0	-0.98
**Glyma.08G049400**	3.51	2.49	0.34	60.9	78.3	95.7	-1.00
**Glyma.08G052400**	2.52	1.49	3.03	34.4	57.8	3.2	-0.99
**Glyma.08G054700**	2.72	2.24	0.47	38.5	50.0	72.0	-1.00
**Glyma.08G055700**	4.54	3.86	0.99	35.4	58.3	75.0	-0.98
**Glyma.08G056700**	4.16	2.77	0.16	9.1	41.0	60.0	-1.00
**Glyma.08G059600**	3.06	2.62	0.17	0	17.0	54.6	-1.00
**Glyma.08G074300**	1.21	0.95	0.05	38.1	55.5	83.3	-1.00
**Glyma.08G081000**	2.74	1.87	0.29	10.5	36.5	52.6	-1.00
**Glyma.08G084800**	8.73	7.78	2.12	24.1	53.4	77.8	-1.00
**Glyma.08G090400**	2.76	2.26	3.47	14.9	38.8	0	-0.94
**Glyma.08G093900**	3.33	3.15	1.75	0	0	50.0	-0.99
**Glyma.08G098400**	2.15	2.65	0.39	12.5	0	40.0	-1.00
**Glyma.08G101100**	3.97	3.45	2.00	4.2	9.4	63.6	-0.95
**Glyma.08G103900**	2.45	3.14	0.16	2.6	0	50.0	-0.92
**Glyma.08G104200**	7.03	5.49	1.34	3.7	15.2	50.0	-0.89
**Glyma.08G107100**	2.15	1.68	0.04	22.7	48.0	68.2	-0.98

**Glyma.08G108000**	6.68	5.54	0.65	24.1	69.5	78.3	-0.95
**Glyma.08G116800**	1.22	0.28	1.82E-04	31.7	71.4	81.0	-1.00
**Glyma.08G120700**	4.61	4.95	0.75	0	0	60.9	-0.98
**Glyma.08G121300**	2.94	2.89	1.14	33.8	62.6	100.0	-0.92
**Glyma.08G125400**	2.61	2.07	0.01	0	0	40.0	-0.93
**Glyma.08G126100**	4.36	3.68	1.27	15.6	91.7	100.0	-0.87
**Glyma.08G126900**	3.81	3.07	0.52	2.3	2.3	43.8	-0.90
**Glyma.08G127600**	3.38	3.32	0.28	0	0	55.0	-1.00
**Glyma.08G130400**	3.77	2.66	2.06	56.7	93.3	100.0	-1.00
**Glyma.08G131700**	2.16	1.75	0.17	0	0	33.3	-0.95
**Glyma.08G132000**	2.24	2.34	0.95	39.6	49.7	79.2	-0.94
**Glyma.08G134600**	0.67	1.80	3.58	39.1	18.4	8.3	-0.85
**Glyma.08G136500**	3.18	2.69	0.77	0	0	60.0	-0.94
**Glyma.08G137000**	3.32	2.51	0.31	0	12.5	34.8	-0.99
**Glyma.08G139300**	1.90	2.23	0.42	0	0	31.8	-0.96
**Glyma.08G141400**	1.58	0.69	4.59E-04	5.2	29.3	37.9	-1.00
**Glyma.08G148100**	1.31	1.18	0	0	0	56.0	-0.99
**Glyma.08G154900**	4.24	3.34	1.89	25.0	45.0	75.0	-0.98
**Glyma.08G161600**	2.10	3.14	0.09	17.3	12.5	59.1	-0.87
**Glyma.08G161900**	1.07	0.41	0	21.1	68.4	79.0	-0.99
**Glyma.08G164700**	1.44	1.12	0.42	52.8	63.5	86.7	-1.00
**Glyma.08G165700**	2.36	1.31	0.48	23.1	54.7	78.1	-0.99
**Glyma.08G165900**	1.31	1.81	3.25	87.5	95.5	54.2	-0.95
**Glyma.08G171900**	2.97	2.75	1.46	22.1	33.8	52.9	-0.99
**Glyma.08G174700**	5.49	4.07	7.11	86.8	77.9	48.5	-0.90
**Glyma.08G177100**	2.29	1.58	0.12	48.8	60.9	90.5	-0.97
**Glyma.08G177200**	3.09	0.80	0.15	20.0	52.0	52.0	-1.00
**Glyma.08G178800**	1.24	1.28	3.21	48.0	64.7	20.0	-0.93
**Glyma.08G180700**	3.40	4.32	3.20	59.1	38.0	79.2	-0.92
**Glyma.08G181000**	5.85	5.04	1.76	12.5	21.9	42.9	-0.99
**Glyma.08G182100**	3.05	2.92	5.57	53.3	59.7	0	-1.00
**Glyma.08G190800**	3.90	2.84	1.12	10.0	65.1	100.0	-1.00
**Glyma.08G191400**	1.46	0.42	0.02	20.8	67.2	78.6	-1.00
**Glyma.08G192300**	1.86	1.56	0.25	56.0	72.7	95.5	-0.99
**Glyma.08G197500**	1.02	0.33	1.54E-05	50.0	71.7	95.2	-0.95
**Glyma.08G199800**	3.55	3.23	1.15	0	9.6	46.4	-1.00
**Glyma.08G204200**	3.95	3.07	2.94	38.3	72.0	72.0	-1.00
**Glyma.08G205700**	2.14	0.70	0.14	18.4	50.2	47.4	-0.97
**Glyma.08G208700**	1.88	1.00	0.33	30.0	76.4	77.8	-0.96
**Glyma.08G209100**	4.62	4.53	5.64	75.0	78.6	42.9	-1.00
**Glyma.08G209200**	3.24	1.87	0.07	12.5	58.0	75.0	-1.00
**Glyma.08G213100**	4.49	3.36	0.09	5.6	11.1	48.2	-0.89
**Glyma.08G213200**	1.29	0.85	7.65E-05	0	1.7	30.8	-0.92

**Glyma.08G215500**	4.31	3.79	0.06	20.7	46.6	79.3	-0.99
**Glyma.08G217900**	3.35	2.74	0.03	7.1	19.0	38.1	-1.00
**Glyma.08G219100**	4.97	3.63	3.62	40.3	71.0	61.3	-0.95
**Glyma.08G220600**	1.89	1.22	0	23.3	53.3	93.3	-1.00
**Glyma.08G220700**	1.54	0.50	0	27.6	63.6	86.4	-0.98
**Glyma.08G222400**	4.47	4.14	2.42	0	10.5	40.0	-1.00
**Glyma.08G224800**	1.78	1.17	0	10.8	13.5	43.2	-0.91
**Glyma.08G225500**	3.96	3.04	1.05	26.1	50.5	68.2	-1.00
**Glyma.08G227000**	4.49	4.12	0.08	0	15.3	50.0	-1.00
**Glyma.08G227900**	1.68	2.05	3.09	43.2	46.9	0	-0.97
**Glyma.08G229300**	1.91	1.83	0.19	24.6	18.5	74.1	-0.99
**Glyma.08G231100**	1.30	0.96	0.09	12.0	16.0	42.1	-0.97
**Glyma.08G232000**	4.26	3.96	4.98	34.7	64.2	9.4	-0.94
**Glyma.08G233500**	1.79	1.89	0.12	70.5	65.6	100.0	-1.00
**Glyma.08G234100**	3.70	0.51	0.14	16.9	52.3	59.1	-0.99
**Glyma.08G236000**	2.75	4.78	5.85	44.0	21.7	2.8	-0.99
**Glyma.08G237900**	2.26	2.38	1.05	12.9	21.6	50.0	-0.94
**Glyma.08G238100**	7.47	7.71	3.78	10.0	15.7	42.9	-0.95
**Glyma.08G238300**	3.34	2.73	0.51	1.9	16.7	44.4	-1.00
**Glyma.08G242500**	1.57	1.89	0.83	7.7	10.9	38.1	-0.89
**Glyma.08G243100**	1.65	0.27	0.05	9.1	43.1	40.9	-0.99
**Glyma.08G243600**	3.36	3.29	0.21	28.5	42.3	87.0	-0.98
**Glyma.08G244700**	0.06	0.16	2.83	90.0	65.0	35.0	-0.90
**Glyma.08G245000**	5.87	3.97	0.81	21.2	66.7	62.5	-0.95
**Glyma.08G248900**	1.30	2.67	1.37	43.3	16.7	53.3	-0.96
**Glyma.08G251400**	2.71	2.86	1.23	11.7	16.7	61.5	-0.98
**Glyma.08G255300**	2.19	1.49	0.79	27.9	46.4	73.3	-0.97
**Glyma.08G257800**	2.57	1.55	0.08	10.7	41.7	47.6	-0.97
**Glyma.08G260400**	2.23	1.65	0.26	4.0	25.0	50.0	-1.00
**Glyma.08G261800**	4.78	5.05	2.39	16.4	23.6	50.0	-0.92
**Glyma.08G261900**	4.48	4.21	0.39	6.1	28.8	43.3	-0.89
**Glyma.08G262800**	4.28	2.85	0.39	6.7	30.0	36.7	-0.99
**Glyma.08G268000**	1.16	0.08	0	5.7	30.5	59.1	-0.87
**Glyma.08G268900**	1.80	1.97	0.90	29.5	24.4	72.2	-1.00
**Glyma.08G270300**	1.61	0.23	5.45E-04	7.1	26.3	46.2	-0.90
**Glyma.08G270900**	2.98	2.78	1.65	6.8	18.2	45.5	-1.00
**Glyma.08G275900**	1.19	0.99	0.14	30.8	51.7	71.4	-0.95
**Glyma.08G276600**	1.92	1.64	0.92	49.8	68.4	88.9	-0.99
**Glyma.08G279300**	1.28	1.55	0.03	8.5	12.8	47.4	-0.94
**Glyma.08G280700**	2.93	1.57	0.59	3.6	31.1	50.0	-0.98
**Glyma.08G284500**	4.46	4.60	1.12	10.7	13.7	43.8	-0.98
**Glyma.08G285100**	2.91	1.01	0.05	25.0	80.4	76.2	-0.98
**Glyma.08G287600**	2.28	2.07	5.35	63.6	71.2	30.8	-0.99

**Glyma.08G289600**	1.14	1.73	0.08	15.2	14.0	44.8	-0.89
**Glyma.08G291600**	1.86	1.32	0.63	21.0	50.0	74.3	-1.00
**Glyma.08G292700**	3.51	2.78	0.91	33.3	60.4	83.3	-1.00
**Glyma.08G292800**	3.31	2.64	2.03	5.0	26.7	40.0	-1.00
**Glyma.08G294800**	0.21	0.77	2.02	77.1	38.9	0	-0.94
**Glyma.08G295700**	3.91	3.16	2.37	8.2	24.3	38.7	-0.99
**Glyma.08G295800**	1.34	1.69	0.31	2.4	1.8	36.7	-0.94
**Glyma.08G297000**	3.35	2.25	0.06	16.7	22.2	52.8	-0.88
**Glyma.08G308300**	1.60	0.29	0	9.1	47.3	45.5	-0.99
**Glyma.08G309300**	3.76	2.24	2.68	14.6	58.3	50.0	-1.00
**Glyma.08G311900**	4.33	3.53	0.55	12.0	32.1	50.0	-1.00
**Glyma.08G321400**	1.48	1.75	0.02	27.1	19.6	82.6	-0.99
**Glyma.08G325700**	1.94	2.20	0.96	79.2	54.2	100.0	-0.96
**Glyma.08G326600**	1.08	0.49	0.01	2.9	18.0	33.3	-0.99
**Glyma.08G328700**	3.35	3.57	1.51	68.2	68.2	100.0	-0.98
**Glyma.08G331100**	1.27	1.53	0.23	0	0	33.3	-0.96
**Glyma.08G336300**	3.08	0.75	6.64E-06	25.0	67.5	71.4	-1.00
**Glyma.08G342100**	0.33	1.61	5.83	23.5	43.0	3.6	-0.86
**Glyma.08G342300**	8.77	8.60	2.51	5.0	20.0	52.6	-0.98
**Glyma.08G343000**	0.61	1.66	0.37	20.0	4.4	37.5	-0.90
**Glyma.08G343500**	3.16	3.06	1.45	25.4	62.5	87.5	-0.85
**Glyma.08G353300**	1.34	0.04	0.05	18.3	40.0	56.7	-0.90
**Glyma.08G361100**	0.54	1.56	0.07	83.3	56.7	86.7	-0.99
**Glyma.08G365700**	3.30	2.32	0.13	5.6	31.3	43.8	-0.99
**Glyma.09G001600**	2.67	2.61	0.12	0	22.2	59.3	-0.95
**Glyma.09G002500**	1.80	0.88	0.40	28.6	50.0	66.7	-0.98
**Glyma.09G003000**	1.78	2.46	2.90	32.1	31.3	0	-0.87
**Glyma.09G004300**	5.96	6.71	4.37	33.7	15.3	50.0	-1.00
**Glyma.09G009500**	2.02	2.41	0.30	3.2	7.8	43.8	-0.92
**Glyma.09G010700**	1.30	0.25	0.02	52.8	97.2	88.9	-0.96
**Glyma.09G011300**	3.23	3.78	0.16	0	0	65.0	-0.94
**Glyma.09G012400**	2.61	1.35	0.08	8.9	26.8	39.3	-0.99
**Glyma.09G015100**	4.14	3.60	1.64	55.6	70.8	87.5	-0.99
**Glyma.09G016100**	2.98	2.36	0.27	28.1	41.2	60.0	-1.00
**Glyma.09G018700**	2.45	1.94	1.06	8.7	37.5	45.8	-0.95
**Glyma.09G024900**	1.54	0.88	0	30.4	69.1	96.4	-1.00
**Glyma.09G026100**	4.37	4.18	2.69	0	0	37.0	-0.99
**Glyma.09G028700**	2.08	0.80	0.26	0	22.2	40.0	-0.96
**Glyma.09G031100**	1.38	0.69	0.24	8.8	36.1	69.2	-0.96
**Glyma.09G037400**	4.36	4.01	3.18	13.6	18.9	61.9	-0.96
**Glyma.09G042500**	1.80	1.07	0.04	34.9	54.8	85.7	-0.98
**Glyma.09G043100**	5.13	5.06	3.61	24.5	44.3	87.5	-0.97
**Glyma.09G044100**	1.29	0.90	0.05	0	1.6	38.5	-0.93

**Glyma.09G044300**	3.17	3.45	1.21	24.7	23.7	58.8	-0.98
**Glyma.09G044700**	3.13	2.65	1.75	0	0	47.6	-0.89
**Glyma.09G045800**	2.28	3.38	5.89	59.0	75.6	21.1	-0.92
**Glyma.09G047500**	3.85	4.21	0.79	5.0	12.5	43.8	-0.91
**Glyma.09G051900**	0.01	1.05	3.94	66.7	81.3	36.4	-0.92
**Glyma.09G052100**	5.22	4.14	3.16	37.6	60.7	71.4	-1.00
**Glyma.09G054300**	2.29	0.27	0.22	6.0	20.9	37.0	-0.86
**Glyma.09G056300**	4.44	2.93	0.31	39.2	77.3	86.4	-0.99
**Glyma.09G059300**	3.70	2.45	1.35	14.5	25.0	45.5	-0.91
**Glyma.09G060300**	1.63	1.56	0.25	1.6	10.0	34.5	-0.98
**Glyma.09G061600**	3.69	3.34	1.41	3.6	26.7	68.8	-1.00
**Glyma.09G061700**	1.38	0.91	1.56E-05	12.5	27.4	54.2	-0.99
**Glyma.09G068500**	2.87	2.23	1.34	11.4	41.7	75.0	-1.00
**Glyma.09G071300**	3.17	0.85	0.01	15.5	47.4	65.6	-0.96
**Glyma.09G071400**	2.64	0.96	0.80	18.8	68.8	70.8	-1.00
**Glyma.09G073000**	4.91	3.53	0.13	33.3	59.3	77.8	-1.00
**Glyma.09G073300**	2.43	1.41	1.22	25.0	63.1	62.5	-0.99
**Glyma.09G078400**	3.81	4.02	5.75	18.8	34.5	0	-0.87
**Glyma.09G079900**	2.75	1.32	0.65	10.8	54.0	100.0	-0.93
**Glyma.09G080400**	1.92	1.65	0.15	7.9	63.2	89.5	-0.88
**Glyma.09G084600**	2.17	2.43	3.57	25.0	37.3	6.7	-0.87
**Glyma.09G085300**	2.71	2.63	1.50	7.1	15.4	50.0	-1.00
**Glyma.09G087700**	5.75	4.63	0.38	37.9	66.0	96.0	-1.00
**Glyma.09G088300**	1.14	0.61	1.70	7.1	32.7	0	-0.91
**Glyma.09G089500**	1.37	0.12	1.28E-06	8.9	71.4	60.7	-0.98
**Glyma.09G089600**	4.66	3.83	0.95	10.4	16.7	54.2	-0.94
**Glyma.09G090600**	3.67	0.47	0.06	3.1	41.2	46.7	-1.00
**Glyma.09G098300**	2.64	1.82	0.24	22.3	45.5	63.6	-1.00
**Glyma.09G107000**	4.45	4.14	2.69	5.0	25.0	65.0	-1.00
**Glyma.09G107400**	2.64	3.03	0.67	5.5	2.4	42.9	-0.98
**Glyma.09G110900**	2.34	2.23	0.04	26.0	40.0	80.0	-0.99
**Glyma.09G112400**	3.95	3.52	2.80	11.1	9.3	51.9	-0.87
**Glyma.09G117600**	2.31	1.04	0.07	6.5	19.4	51.6	-0.87
**Glyma.09G129300**	2.36	2.63	3.84	27.0	39.2	7.7	-0.88
**Glyma.09G134500**	4.48	1.61	0.61	0	47.5	46.7	-1.00
**Glyma.09G145900**	2.01	1.09	4.05	60.9	71.7	39.1	-0.98
**Glyma.09G149400**	1.32	0.37	0.05	16.8	61.7	71.4	-1.00
**Glyma.09G149600**	3.80	1.63	0.13	8.3	53.1	80.0	-0.97
**Glyma.09G154100**	1.43	1.27	0.16	10.6	15.0	61.5	-1.00
**Glyma.09G155500**	8.96	7.58	3.45	1.9	23.1	34.6	-1.00
**Glyma.09G158200**	2.32	2.73	4.62	49.8	41.5	19.4	-0.98
**Glyma.09G162400**	2.94	2.24	0.13	15.2	29.9	47.8	-1.00
**Glyma.09G166100**	3.07	2.66	0.66	9.4	15.6	40.6	-0.99

**Glyma.09G168500**	1.22	1.22	0.14	0	0	33.3	-1.00
**Glyma.09G173800**	2.22	1.11	0.04	38.0	64.0	76.0	-1.00
**Glyma.09G176100**	4.39	3.57	1.65	0	0	30.8	-0.86
**Glyma.09G178200**	3.37	2.23	0.32	7.9	46.5	50.0	-0.95
**Glyma.09G181100**	4.58	2.77	0.21	16.7	45.8	50.0	-0.99
**Glyma.09G185000**	3.53	3.03	2.22	34.8	73.3	95.7	-0.99
**Glyma.09G191300**	3.18	1.98	0.03	20.5	49.0	66.7	-1.00
**Glyma.09G192000**	2.92	2.98	0.20	14.0	0	79.4	-0.99
**Glyma.09G194900**	2.60	2.22	0.28	4.4	7.9	37.0	-0.98
**Glyma.09G196700**	2.23	2.48	0.23	11.9	0	50.0	-1.00
**Glyma.09G199300**	2.40	2.03	1.25	16.0	15.1	46.2	-0.90
**Glyma.09G200800**	2.01	0.72	0.10	7.9	55.4	60.7	-1.00
**Glyma.09G205800**	1.19	1.65	0.58	35.7	0	92.0	-0.99
**Glyma.09G209600**	2.13	2.18	3.19	45.2	39.6	0	-1.00
**Glyma.09G210900**	5.19	3.39	0.31	56.3	72.9	87.5	-0.97
**Glyma.09G216900**	2.05	1.27	0.04	26.0	62.5	76.9	-0.98
**Glyma.09G223300**	2.25	1.78	3.21	38.9	61.1	25.0	-0.90
**Glyma.09G225700**	1.49	0.72	0.39	14.6	76.8	87.5	-1.00
**Glyma.09G226300**	2.73	2.03	0.23	43.0	58.7	76.9	-1.00
**Glyma.09G226500**	0	0.63	3.18	65.1	36.1	14.3	-0.86
**Glyma.09G229200**	3.10	0.78	0.01	12.1	52.5	70.0	-0.98
**Glyma.09G234700**	5.49	5.90	4.77	0	0	57.1	-0.89
**Glyma.09G236200**	1.73	0.60	4.24E-05	27.0	74.0	100.0	-0.99
**Glyma.09G247100**	5.40	4.59	3.05	41.7	62.4	81.8	-1.00
**Glyma.09G249700**	2.72	1.87	1.64	61.7	95.1	90.5	-0.96
**Glyma.09G259200**	1.35	1.74	0.46	0	0	36.4	-0.92
**Glyma.09G260900**	5.99	4.06	0.38	30.8	51.4	60.9	-1.00
**Glyma.09G263700**	1.62	1.82	0.25	5.9	0	56.5	-1.00
**Glyma.09G264000**	2.48	2.52	0.93	13.6	5.6	63.6	-1.00
**Glyma.09G266500**	2.71	2.87	1.40	39.3	51.0	78.6	-0.90
**Glyma.09G269300**	4.85	5.02	3.12	38.1	20.7	59.5	-0.95
**Glyma.09G271500**	2.19	1.69	0.02	0	0	41.7	-0.93
**Glyma.09G277400**	4.34	3.10	2.33	49.1	70.8	84.2	-0.99
**Glyma.09G285400**	3.59	4.20	1.91	12.0	10.1	56.0	-0.92
**Glyma.10G000200**	2.12	1.04	0.30	24.3	41.1	66.7	-0.93
**Glyma.10G002500**	0.95	0.98	2.35	59.3	58.9	15.4	-1.00
**Glyma.10G004300**	7.10	6.11	6.01	13.3	47.8	66.7	-0.95
**Glyma.10G006100**	2.45	2.65	0.19	10.7	12.5	50.0	-0.98
**Glyma.10G010200**	5.18	4.38	0.30	2.8	9.8	35.7	-0.97
**Glyma.10G017700**	1.87	1.50	0.69	0	0	40.0	-0.92
**Glyma.10G017800**	2.59	2.37	1.09	0	0	62.5	-0.98
**Glyma.10G027100**	2.67	3.30	3.92	47.9	52.8	0	-0.88
**Glyma.10G027200**	1.67	0.88	0.10	45.7	60.9	82.6	-0.97

**Glyma.10G030400**	3.87	3.19	0.11	0	0	40.0	-0.92
**Glyma.10G031200**	5.08	2.52	0.18	10.0	31.4	40.0	-0.99
**Glyma.10G032000**	1.36	0.75	0.17	49.6	80.8	90.0	-0.99
**Glyma.10G035700**	2.42	1.65	0.04	13.3	48.3	50.0	-0.89
**Glyma.10G040100**	1.40	0.87	0	3.3	10.7	54.6	-0.93
**Glyma.10G042000**	5.91	4.98	0.82	4.8	5.3	45.5	-0.88
**Glyma.10G042800**	0.31	1.19	2.39	51.1	51.9	16.0	-0.96
**Glyma.10G045500**	2.23	0.41	0	9.3	30.2	42.9	-0.96
**Glyma.10G046600**	3.76	3.40	2.64	0	7.7	33.3	-0.98
**Glyma.10G047600**	1.42	1.61	3.15	53.3	65.0	20.0	-0.95
**Glyma.10G052600**	3.72	2.36	0.27	20.7	52.8	65.5	-1.00
**Glyma.10G053300**	2.72	2.54	1.42	10.8	14.7	44.4	-1.00
**Glyma.10G054200**	2.01	0.21	0.01	14.7	62.9	50.0	-0.95
**Glyma.10G054600**	2.57	1.44	0.09	27.3	57.9	80.0	-1.00
**Glyma.10G057000**	3.78	2.96	1.72	2.9	34.8	57.6	-1.00
**Glyma.10G063800**	2.48	1.53	1.04	29.2	80.5	95.8	-1.00
**Glyma.10G067500**	1.26	0.50	0	55.5	87.8	89.7	-0.97
**Glyma.10G072600**	0	1.53E-06	2.44	41.7	46.0	13.3	-0.99
**Glyma.10G075000**	2.89	1.94	0.02	25.2	49.2	79.2	-0.99
**Glyma.10G078200**	1.47	0.68	0.07	6.3	44.2	61.5	-1.00
**Glyma.10G078400**	6.84	5.59	7.02	7.7	50.0	19.2	-0.90
**Glyma.10G079400**	1.74	0.74	0.04	5.6	50.0	51.9	-0.97
**Glyma.10G088400**	2.19	0.88	0.11	0	26.7	37.5	-1.00
**Glyma.10G089200**	1.38	1.14	0.07	9.7	24.2	43.5	-0.98
**Glyma.10G089700**	1.39	0.28	0.14	4.3	25.7	37.9	-0.95
**Glyma.10G090300**	1.50	1.80	0	45.9	35.8	85.7	-1.00
**Glyma.10G091800**	2.66	1.28	2.41E-03	12.1	45.5	51.7	-0.99
**Glyma.10G094000**	3.55	1.47	0.04	46.7	93.3	90.9	-0.98
**Glyma.10G094600**	1.41	0.64	0.11	21.1	65.7	81.0	-1.00
**Glyma.10G097800**	2.70	1.88	0.07	47.5	65.0	85.0	-1.00
**Glyma.10G101200**	3.91	3.45	0.26	7.5	18.0	45.5	-1.00
**Glyma.10G104100**	2.80	2.41	0.53	16.0	29.2	60.0	-1.00
**Glyma.10G104300**	3.84	3.52	2.31	11.5	42.3	61.5	-0.94
**Glyma.10G105900**	3.58	2.70	1.84	16.8	29.2	50.0	-0.95
**Glyma.10G106500**	1.59	1.29	0.43	0	5.8	30.8	-0.99
**Glyma.10G106600**	3.58	1.76	0.06	16.7	47.6	52.4	-1.00
**Glyma.10G107700**	1.50	0.79	0.22	16.1	32.7	46.4	-0.99
**Glyma.10G108400**	3.15	1.90	0.11	25.5	58.3	68.8	-0.99
**Glyma.10G111800**	2.93	1.89	0.70	22.9	81.3	83.3	-0.95
**Glyma.10G113100**	1.77	0.74	1.11	50.0	88.9	94.4	-0.92
**Glyma.10G113500**	2.31	2.02	0.03	24.8	41.4	69.0	-0.99
**Glyma.10G118100**	2.03	0.60	6.97E-05	2.8	25.9	45.5	-0.95
**Glyma.10G119100**	1.89	2.82	0.09	37.2	15.3	55.0	-1.00

**Glyma.10G119900**	1.64	1.36	0.04	3.1	39.1	71.4	-0.96
**Glyma.10G120700**	4.12	1.71	0.04	27.7	77.8	86.4	-1.00
**Glyma.10G122200**	5.24	4.88	6.66	43.2	66.4	27.3	-0.87
**Glyma.10G124800**	5.18	4.96	6.63	33.8	35.8	3.5	-1.00
**Glyma.10G133500**	5.03	3.30	1.45	5.3	23.8	35.7	-0.99
**Glyma.10G133900**	1.41	1.38	0.04	0	16.7	59.1	-0.97
**Glyma.10G134100**	3.43	2.74	2.34	5.9	21.1	42.9	-0.94
**Glyma.10G137400**	1.37	0.39	0.03	48.6	88.7	100.0	-1.00
**Glyma.10G140900**	3.15	4.86	7.60	56.1	61.1	30.8	-0.97
**Glyma.10G141000**	3.38	3.95	4.81	38.3	22.9	4.0	-0.98
**Glyma.10G144100**	1.63	0.44	0.04	8.3	22.2	40.0	-0.90
**Glyma.10G148100**	2.83	2.74	1.26	3.3	0	33.3	-0.98
**Glyma.10G150800**	7.37	6.25	6.29	13.2	39.3	46.2	-0.98
**Glyma.10G151900**	4.62	4.54	5.80	26.6	46.9	9.4	-0.86
**Glyma.10G153200**	3.74	3.04	0.87	5.6	20.8	60.0	-0.98
**Glyma.10G157900**	3.06	1.96	3.87	51.7	82.8	44.8	-0.90
**Glyma.10G162500**	2.56	2.14	1.28	21.4	26.2	75.0	-0.94
**Glyma.10G164100**	6.01	4.13	2.55	13.8	44.8	55.2	-1.00
**Glyma.10G166400**	1.94	0.80	1.29	2.1	35.4	25.0	-1.00
**Glyma.10G173300**	3.70	4.04	6.42	26.2	43.2	0	-0.90
**Glyma.10G175300**	0.68	0.56	1.79	16.0	30.0	0	-0.91
**Glyma.10G177300**	1.40	0.34	0.13	15.2	57.2	74.2	-0.98
**Glyma.10G177600**	1.23	1.02	0.14	0	1.6	45.5	-0.98
**Glyma.10G179100**	1.40	1.83	0.26	6.0	0	38.5	-0.97
**Glyma.10G189700**	2.72	0.53	0.16	0	90.6	71.4	-0.97
**Glyma.10G190100**	2.88	3.34	0.84	2.4	0	40.9	-0.96
**Glyma.10G191300**	1.92	1.69	0	25.0	34.6	57.7	-0.99
**Glyma.10G193900**	10.80	10.98	8.54	62.9	69.4	93.6	-0.94
**Glyma.10G205200**	1.79	1.03	0.29	10.0	19.4	50.0	-0.90
**Glyma.10G208700**	2.13	1.77	1.08	11.6	14.1	45.2	-0.93
**Glyma.10G211200**	2.03	0.72	0.16	15.0	63.3	80.0	-1.00
**Glyma.10G215800**	1.99	2.17	0.41	65.8	48.5	100.0	-0.98
**Glyma.10G221000**	1.32	1.26	0	0	0	30.4	-1.00
**Glyma.10G222900**	1.61	1.54	3.06	21.0	30.2	0	-0.96
**Glyma.10G223700**	4.91	3.95	0.18	48.8	73.8	94.1	-1.00
**Glyma.10G232100**	5.39	7.67	5.65	78.9	32.9	92.3	-0.97
**Glyma.10G232300**	2.93	4.69	5.71	33.1	20.3	0	-1.00
**Glyma.10G235100**	5.71	4.06	3.19	45.0	70.5	81.8	-0.99
**Glyma.10G240700**	3.36	3.10	0.14	2.6	28.6	53.9	-0.94
**Glyma.10G241800**	2.69	2.70	0.24	30.6	55.6	100.0	-0.93
**Glyma.10G250100**	3.73	4.32	1.25	13.3	16.7	66.7	-0.90
**Glyma.10G255500**	5.65	4.55	0.94	0	25.2	35.7	-0.99
**Glyma.10G256400**	2.12	1.93	0.71	0	0	50.0	-0.98

**Glyma.10G259400**	1.59	2.07	0.51	74.4	44.2	76.9	-0.86
**Glyma.10G259500**	3.53	2.76	0.48	0	0	36.4	-0.88
**Glyma.10G259600**	2.05	1.86	0.52	0	0	36.0	-0.98
**Glyma.10G261800**	4.06	3.41	2.68	35.7	64.1	84.6	-1.00
**Glyma.10G284300**	2.54	1.94	0.30	58.9	70.3	91.7	-1.00
**Glyma.10G288700**	1.13	0.94	2.14	74.6	93.3	46.7	-0.95
**Glyma.10G294300**	5.22	4.09	3.76	2.2	26.5	45.5	-0.95
**Glyma.10G294600**	5.09	4.77	1.43	0	0	33.3	-0.98
**Glyma.10G296800**	2.28	1.80	0.51	32.3	52.8	75.0	-1.00
**Glyma.11G001500**	6.90	6.45	4.64	0	0	42.1	-0.94
**Glyma.11G005100**	1.57	1.27	0.03	37.7	41.7	71.4	-0.99
**Glyma.11G015600**	3.66	3.15	0.34	0	0	36.0	-0.95
**Glyma.11G033800**	3.93	3.37	2.29	6.0	50.0	75.8	-0.98
**Glyma.11G039700**	4.17	4.17	1.13	0	0	88.9	-1.00
**Glyma.11G042300**	1.55	0.98	0.46	27.3	56.3	73.9	-1.00
**Glyma.11G043900**	3.27	4.19	7.72	70.8	60.0	40.0	-0.95
**Glyma.11G048700**	3.18	2.62	0.15	17.9	31.8	53.3	-1.00
**Glyma.11G051500**	1.73	0.87	0.13	22.1	50.0	62.5	-1.00
**Glyma.11G053400**	6.61	5.21	0.59	26.1	64.8	63.6	-0.92
**Glyma.11G054300**	0.89	1.27	0.18	57.9	8.8	63.2	-0.87
**Glyma.11G055300**	0.02	0.41	3.58	63.2	35.7	0	-0.91
**Glyma.11G073100**	3.71	4.16	0.68	74.1	61.1	92.6	-0.99
**Glyma.11G077300**	1.75	1.99	0.57	1.8	0	32.1	-0.98
**Glyma.11G080700**	3.92	3.85	0.86	36.6	40.0	73.7	-1.00
**Glyma.11G081600**	3.16	3.46	2.14	32.7	39.0	70.4	-0.89
**Glyma.11G087400**	5.56	3.95	3.03	29.6	56.7	63.3	-1.00
**Glyma.11G089100**	1.46	1.52	2.52	32.1	34.0	0	-1.00
**Glyma.11G093700**	1.63	1.66	2.67	59.4	77.3	6.7	-0.97
**Glyma.11G096200**	4.15	3.66	0.89	2.4	26.3	81.0	-1.00
**Glyma.11G100100**	1.70	1.67	0.52	0	0	53.3	-1.00
**Glyma.11G101400**	6.18	4.39	1.51	25.0	51.9	84.6	-0.95
**Glyma.11G106100**	1.70	1.65	6.36E-05	11.0	18.9	51.4	-0.99
**Glyma.11G107700**	5.24	4.64	2.68	41.8	73.3	77.3	-0.87
**Glyma.11G119500**	0.85	0.23	3.27	42.3	40.6	0	-0.99
**Glyma.11G122400**	1.71	1.87	0.38	0	0	54.6	-0.99
**Glyma.11G122600**	1.01	0.73	4.04	73.1	81.3	50.0	-0.97
**Glyma.11G125400**	1.66	2.04	3.90	50.0	75.0	0	-0.92
**Glyma.11G130200**	1.62	0.43	0.02	41.0	76.0	88.0	-1.00
**Glyma.11G141500**	1.69	1.82	2.92	42.9	28.6	4.8	-0.95
**Glyma.11G141800**	5.40	3.20	0.49	66.2	83.3	100.0	-0.94
**Glyma.11G143200**	6.35	5.64	4.89	6.7	24.6	38.1	-1.00
**Glyma.11G145400**	3.32	3.02	2.20	0	0	70.8	-0.94
**Glyma.11G145500**	3.11	3.13	0.40	13.9	37.5	80.0	-0.93

**Glyma.11G148900**	3.76	3.04	0.99	8.1	22.6	44.8	-1.00
**Glyma.11G149300**	1.03	1.01	0	7.5	14.0	39.3	-0.98
**Glyma.11G150400**	7.54	6.24	2.63	13.8	54.2	72.2	-1.00
**Glyma.11G150700**	1.93	1.16	0.10	0	15.9	31.8	-1.00
**Glyma.11G151100**	1.71	1.93	4.86	36.2	48.1	16.7	-0.92
**Glyma.11G151300**	4.91	3.58	5.27	16.9	61.2	4.8	-0.99
**Glyma.11G151800**	4.11	3.40	0.39	13.5	32.7	76.9	-0.99
**Glyma.11G155300**	2.45	2.07	1.28	15.0	40.0	45.8	-0.90
**Glyma.11G155900**	4.18	3.41	2.31	10.3	33.8	55.9	-1.00
**Glyma.11G156500**	3.25	2.78	1.18	20.4	60.9	69.6	-0.87
**Glyma.11G158200**	0.32	0.04	2.26	74.1	91.4	55.2	-0.90
**Glyma.11G162700**	0.64	0.98	1.94	83.3	90.9	60.0	-0.92
**Glyma.11G164700**	7.08	4.37	0.39	18.2	43.3	50.0	-1.00
**Glyma.11G165100**	2.07	1.13	0.12	28.2	71.8	77.3	-0.97
**Glyma.11G166500**	3.19	2.39	0.16	5.0	33.3	76.5	-0.99
**Glyma.11G171300**	1.62	0.44	0.07	54.3	73.8	92.6	-0.93
**Glyma.11G173200**	1.84	0.08	0	2.4	48.4	47.6	-1.00
**Glyma.11G174200**	2.03	0	0.05	17.9	59.4	61.5	-1.00
**Glyma.11G177800**	4.80	2.70	0.50	30.7	56.2	66.7	-0.99
**Glyma.11G177900**	5.19	3.49	0.72	17.4	52.2	60.9	-1.00
**Glyma.11G178100**	4.91	4.08	2.65	6.5	33.0	43.5	-0.98
**Glyma.11G178200**	5.00	2.88	0.76	7.6	44.4	50.0	-1.00
**Glyma.11G182600**	1.91	0.43	0.07	17.9	59.9	50.0	-0.95
**Glyma.11G189100**	1.11	1.46	0.38	25.8	22.2	64.3	-0.94
**Glyma.11G189600**	2.62	1.82	0.28	2.1	20.0	36.4	-1.00
**Glyma.11G190200**	6.09	6.28	5.08	2.6	4.5	40.0	-0.97
**Glyma.11G190600**	1.11	0.16	0	3.1	45.0	50.0	-1.00
**Glyma.11G192100**	3.91	3.69	2.20	5.3	0	31.6	-0.94
**Glyma.11G195900**	0.02	0.54	1.32	31.5	41.3	0	-0.87
**Glyma.11G201500**	4.29	2.94	3.84	51.5	87.5	81.3	-0.91
**Glyma.11G204200**	1.70	1.41	0.18	4.4	15.6	36.4	-1.00
**Glyma.11G208600**	4.68	3.49	1.54	32.5	70.0	66.7	-0.90
**Glyma.11G209600**	2.36	1.89	0.69	56.4	75.7	100.0	-1.00
**Glyma.11G211000**	1.24	1.76	0.63	26.5	10.7	71.4	-0.94
**Glyma.11G212500**	1.44	0.95	3.89E-04	11.7	25.8	47.1	-1.00
**Glyma.11G214800**	4.43	4.06	3.10	7.4	20.7	38.2	-1.00
**Glyma.11G215600**	1.81	0.52	0.11	32.5	50.0	72.0	-0.90
**Glyma.11G216300**	2.75	2.05	1.24	17.5	33.9	81.8	-0.93
**Glyma.11G216900**	2.18	1.65	0.42	22.1	50.0	61.9	-0.95
**Glyma.11G218700**	2.81	3.03	1.39	11.7	18.2	45.5	-0.93
**Glyma.11G219600**	2.89	0.63	0.04	22.5	80.0	78.3	-1.00
**Glyma.11G222800**	2.85	2.53	0.30	44.1	63.0	92.6	-0.99
**Glyma.11G226800**	7.40	5.81	4.96	33.3	65.9	57.6	-0.91

**Glyma.11G227600**	4.47	4.04	0.13	52.1	88.2	100.0	-0.86
**Glyma.11G228300**	1.72	0.75	4.58E-03	2.5	33.6	40.7	-0.99
**Glyma.11G233700**	1.11	0.32	0.10	17.5	57.4	51.9	-0.96
**Glyma.11G234300**	1.70	1.36	4.71	33.3	56.0	5.0	-0.91
**Glyma.11G234800**	4.91	3.37	0.10	39.6	60.4	75.0	-0.99
**Glyma.11G235500**	2.46	2.48	4.82	48.1	58.4	10.0	-0.98
**Glyma.11G239200**	2.20	1.75	0.15	2.8	0	33.3	-0.91
**Glyma.11G241300**	3.82	1.70	0.07	21.9	54.4	58.6	-1.00
**Glyma.11G241700**	5.96	5.62	4.89	12.0	33.1	43.5	-0.95
**Glyma.11G243000**	3.17	3.01	1.51	14.8	8.8	43.3	-0.95
**Glyma.11G243700**	3.61	3.28	1.29	6.6	29.5	45.0	-0.93
**Glyma.11G249000**	2.22	2.48	3.33	58.3	55.4	8.3	-0.99
**Glyma.11G253200**	1.85	0.29	7.88E-04	21.7	66.7	72.7	-1.00
**Glyma.11G254000**	1.98	1.95	0.34	0	0	53.9	-1.00
**Glyma.12G005300**	5.15	4.27	2.78	22.4	52.1	68.2	-1.00
**Glyma.12G006000**	1.77	2.05	0.59	49.2	45.6	94.1	-0.98
**Glyma.12G006300**	2.67	1.27	0.06	30.0	72.0	88.0	-1.00
**Glyma.12G010500**	1.60	1.15	0.05	43.9	44.6	76.0	-0.92
**Glyma.12G015500**	1.10	0.28	5.01E-04	3.3	33.3	25.0	-0.90
**Glyma.12G025500**	6.42	5.95	5.00	19.1	29.8	60.0	-0.98
**Glyma.12G027300**	5.87	4.15	0.90	37.0	75.9	92.6	-1.00
**Glyma.12G032400**	4.07	4.36	2.75	29.9	16.7	75.0	-1.00
**Glyma.12G032500**	2.57	2.91	3.67	32.7	11.1	0	-0.89
**Glyma.12G033500**	3.53	3.51	1.03	30.9	41.7	69.2	-0.97
**Glyma.12G040500**	2.48	2.05	0.48	13.3	30.0	60.0	-1.00
**Glyma.12G040800**	3.47	3.33	0.37	2.3	20.0	50.0	-0.96
**Glyma.12G042500**	1.82	1.67	1.62E-05	44.9	54.4	85.7	-1.00
**Glyma.12G047400**	2.08	1.79	0.29	44.0	64.9	87.0	-0.97
**Glyma.12G050800**	1.21	1.00	0.18	17.3	33.3	52.2	-0.98
**Glyma.12G056700**	5.39	2.39	0.47	0	20.6	33.3	-0.95
**Glyma.12G058000**	2.72	1.51	0.50	0	40.0	70.0	-0.99
**Glyma.12G061300**	4.21	3.93	1.83	0	0	41.2	-0.98
**Glyma.12G063000**	3.03	2.75	1.50	20.0	20.1	53.1	-0.97
**Glyma.12G064300**	7.51	6.09	2.56	11.3	38.7	54.8	-1.00
**Glyma.12G064700**	2.98	2.69	1.56	11.7	24.7	50.0	-1.00
**Glyma.12G065200**	5.22	3.47	1.74	14.4	30.6	50.0	-0.94
**Glyma.12G067800**	2.15	2.25	0.18	23.4	35.1	56.3	-0.91
**Glyma.12G070300**	3.83	3.80	0.61	3.6	27.4	57.1	-0.90
**Glyma.12G074500**	5.59	3.47	1.06	51.6	84.4	91.3	-1.00
**Glyma.12G076400**	2.80	1.73	0.22	4.4	32.6	52.2	-1.00
**Glyma.12G085100**	1.61	1.54	0.14	16.7	33.8	60.0	-0.94
**Glyma.12G086700**	1.03	0.27	1.42	11.2	48.0	0	-0.98
**Glyma.12G087800**	2.70	1.79	1.19	23.5	65.9	64.7	-0.95

**Glyma.12G091700**	2.63	2.37	1.47	33.3	47.2	88.9	-1.00
**Glyma.12G092000**	6.14	4.88	0.35	29.3	61.3	71.0	-0.98
**Glyma.12G092200**	2.24	2.47	0.23	30.4	16.0	50.0	-0.97
**Glyma.12G092300**	2.23	1.94	3.20	21.5	43.9	7.1	-0.88
**Glyma.12G093100**	1.09	1.07	2.67	26.7	52.7	0	-0.87
**Glyma.12G095500**	2.52	1.10	0	2.9	39.6	53.9	-1.00
**Glyma.12G096300**	1.45	1.45	0.06	8.9	12.5	46.4	-1.00
**Glyma.12G098900**	1.80	1.29	2.80	26.4	49.2	0	-0.97
**Glyma.12G101800**	3.04	2.24	0.14	55.0	72.0	86.7	-1.00
**Glyma.12G102600**	1.07	0.39	0	8.6	32.6	47.1	-0.99
**Glyma.12G105300**	4.45	3.67	0.96	33.2	45.5	66.7	-0.99
**Glyma.12G105900**	3.93	2.21	0.31	0	18.2	50.0	-0.90
**Glyma.12G108500**	2.22	0.76	0.03	2.3	16.1	36.4	-0.90
**Glyma.12G110700**	2.05	1.42	1.98E-04	45.2	57.5	95.2	-0.97
**Glyma.12G112900**	4.09	3.08	2.08	50.0	73.9	92.9	-0.99
**Glyma.12G114000**	0.60	1.26	2.47	55.3	36.8	10.5	-0.98
**Glyma.12G115400**	3.60	2.61	1.90	37.9	87.0	83.3	-0.94
**Glyma.12G116400**	1.19	0.01	0.02	0	17.0	30.4	-0.90
**Glyma.12G116900**	6.14	6.69	7.75	47.4	57.9	5.3	-0.92
**Glyma.12G117000**	1.78	4.29	6.48	33.3	12.5	0	-0.88
**Glyma.12G120500**	1.82	1.79	3.16	85.7	96.4	50.0	-0.98
**Glyma.12G121200**	2.31	1.84	0.07	0	6.2	31.6	-0.99
**Glyma.12G122900**	6.81	5.71	2.53	45.0	69.1	88.5	-1.00
**Glyma.12G129900**	0.04	0.01	2.44	77.5	60.0	40.0	-0.88
**Glyma.12G132500**	1.11	1.33	0.04	9.8	9.3	76.5	-0.97
**Glyma.12G133700**	1.51	0.58	0.12	5.4	26.8	36.8	-1.00
**Glyma.12G134500**	3.88	3.80	2.63	0	0	36.8	-1.00
**Glyma.12G137100**	0.42	0.55	2.96	24.1	39.7	6.9	-0.87
**Glyma.12G142900**	1.75	0.50	0	0	18.6	33.3	-0.96
**Glyma.12G148200**	2.78	2.65	0.06	18.8	31.8	93.8	-1.00
**Glyma.12G150300**	1.81	0.97	3.39E-06	40.7	64.8	77.8	-1.00
**Glyma.12G151500**	2.51	2.92	0.69	0	0	30.8	-0.95
**Glyma.12G156800**	3.02	2.19	1.02	13.0	42.3	60.0	-1.00
**Glyma.12G158800**	4.67	4.22	2.63	3.1	29.2	57.9	-0.99
**Glyma.12G159300**	6.86	5.95	3.79	13.9	36.4	54.6	-1.00
**Glyma.12G160800**	3.90	4.08	1.84	40.0	37.8	75.0	-1.00
**Glyma.12G161500**	6.28	4.90	0.32	13.8	37.4	55.2	-1.00
**Glyma.12G162700**	3.10	2.47	0.89	48.1	66.4	86.7	-1.00
**Glyma.12G167300**	1.90	1.95	0.86	43.0	50.0	87.0	-0.98
**Glyma.12G168500**	6.19	4.90	2.91	2.2	22.1	34.8	-1.00
**Glyma.12G169800**	2.82	3.50	1.51	23.7	15.1	47.4	-0.96
**Glyma.12G171400**	5.32	5.07	2.84	0	0	58.8	-0.98
**Glyma.12G172600**	2.87	2.60	0.01	11.1	31.3	100.0	-1.00

**Glyma.12G172800**	3.78	3.62	2.77	35.0	44.0	70.0	-1.00
**Glyma.12G179100**	4.24	2.12	0.09	38.8	68.0	70.8	-1.00
**Glyma.12G180000**	2.69	1.54	0.29	36.4	69.2	92.3	-0.99
**Glyma.12G180400**	1.41	0.64	0.30	53.1	89.8	100.0	-1.00
**Glyma.12G185700**	3.16	2.99	1.89	13.7	13.1	45.5	-0.98
**Glyma.12G191000**	2.01	0.46	0.01	10.7	33.3	58.3	-0.90
**Glyma.12G192100**	1.55	2.41	3.28	28.9	35.7	0	-0.86
**Glyma.12G193500**	3.12	2.72	0.42	23.7	58.1	72.7	-0.89
**Glyma.12G193700**	2.66	1.34	0.13	20.5	76.0	95.7	-1.00
**Glyma.12G197200**	2.66	2.74	0.24	9.3	10.7	63.6	-1.00
**Glyma.12G198500**	2.48	1.07	0.31	36.4	60.9	66.7	-1.00
**Glyma.12G199300**	1.24	0.24	0	33.3	67.5	72.7	-1.00
**Glyma.12G199400**	6.19	4.69	0.32	30.0	53.3	63.3	-1.00
**Glyma.12G204800**	2.15	2.64	0	30.0	35.0	90.0	-0.91
**Glyma.12G206500**	1.33	0.40	0	56.2	90.9	79.0	-0.85
**Glyma.12G212100**	2.55	1.90	0.20	8.1	17.7	40.0	-0.99
**Glyma.12G216500**	2.18	0.46	0	7.1	29.2	46.2	-0.94
**Glyma.12G216900**	2.21	2.34	0.09	50.7	35.2	66.7	-0.91
**Glyma.12G219000**	6.10	6.12	3.33	22.5	34.3	70.0	-0.97
**Glyma.12G224500**	2.95	2.47	1.19	19.6	52.2	82.6	-0.99
**Glyma.12G224600**	0.85	1.17	1.96	30.6	20.5	0	-0.99
**Glyma.12G224700**	1.64	2.05	0.19	8.1	14.5	38.5	-0.86
**Glyma.12G226100**	3.38	3.47	0.47	75.2	67.6	100.0	-0.99
**Glyma.12G226300**	2.34	2.45	0.64	0	0	93.3	-1.00
**Glyma.12G227400**	3.88	3.70	2.24	27.0	44.6	75.7	-0.98
**Glyma.12G228700**	1.75	1.71	0.53	10.1	10.4	61.0	-1.00
**Glyma.12G236900**	2.40	2.50	4.07	44.7	31.9	0	-0.97
**Glyma.12G242000**	1.95	1.76	2.85	43.9	61.9	28.6	-0.90
**Glyma.13G001200**	2.22	1.57	0.81	4.2	28.4	52.9	-1.00
**Glyma.13G008000**	2.99	1.20	0.13	36.5	65.7	70.3	-1.00
**Glyma.13G010100**	2.43	1.70	0.52	18.5	38.9	55.6	-1.00
**Glyma.13G010500**	3.20	3.12	4.15	33.9	33.0	0	-1.00
**Glyma.13G011100**	1.16	0.31	0.05	1.3	45.7	57.9	-1.00
**Glyma.13G025800**	2.94	1.76	0.47	16.7	56.3	85.7	-0.99
**Glyma.13G028400**	6.40	6.43	7.91	46.5	34.1	8.3	-0.95
**Glyma.13G028500**	1.10	0.46	0.01	3.7	35.2	33.3	-0.93
**Glyma.13G030700**	1.91	1.87	0.33	0	2.9	63.6	-1.00
**Glyma.13G031200**	5.83	5.04	4.49	21.9	45.3	62.5	-0.99
**Glyma.13G031500**	1.87	0.32	0	19.4	54.2	60.0	-1.00
**Glyma.13G037000**	0.15	0.97	2.43	64.3	71.4	30.0	-0.94
**Glyma.13G038600**	2.08	1.92	0.07	3.8	29.3	65.0	-0.96
**Glyma.13G039600**	2.24	0.16	0	0	28.3	61.1	-0.86
**Glyma.13G041500**	3.77	3.60	4.77	45.7	60.0	10.0	-0.98

**Glyma.13G042800**	2.54	1.13	0.94	64.1	84.7	100.0	-0.93
**Glyma.13G042900**	0.96	0.53	2.30	48.0	38.7	15.0	-0.92
**Glyma.13G047300**	1.46	1.18	0.04	37.9	53.2	78.6	-0.99
**Glyma.13G048200**	3.77	2.72	0.42	0	23.8	38.9	-1.00
**Glyma.13G048500**	2.91	1.91	0.66	0	17.5	30.0	-1.00
**Glyma.13G049400**	1.31	0.82	0.12	25.0	41.1	63.6	-0.99
**Glyma.13G051000**	3.13	2.88	0.07	22.4	62.8	84.6	-0.87
**Glyma.13G055400**	6.93	6.36	3.93	2.9	21.1	54.6	-1.00
**Glyma.13G058800**	2.02	1.87	0.18	5.6	0	30.0	-0.95
**Glyma.13G059200**	3.03	2.89	3.90	83.0	90.2	60.0	-0.99
**Glyma.13G059400**	3.77	3.31	1.63	11.5	25.0	62.5	-1.00
**Glyma.13G061200**	3.29	2.52	1.82	6.1	12.1	45.5	-0.85
**Glyma.13G062800**	2.57	1.26	0.22	9.8	31.8	47.8	-0.98
**Glyma.13G063600**	2.69	2.97	4.45	77.3	67.5	23.1	-1.00
**Glyma.13G063700**	2.68	1.45	3.49E-06	29.5	74.0	88.5	-1.00
**Glyma.13G064000**	2.02	1.42	0.62	2.9	30.0	44.1	-0.99
**Glyma.13G068100**	1.77	1.11	0.45	7.1	35.8	76.0	-0.97
**Glyma.13G069000**	1.16	1.46	3.95	66.6	81.5	32.0	-0.94
**Glyma.13G069400**	3.16	2.89	0.82	28.6	28.0	75.0	-0.98
**Glyma.13G069800**	3.69	3.37	2.39	0	12.5	39.3	-1.00
**Glyma.13G071800**	5.91	4.77	1.40	6.8	47.7	54.6	-0.95
**Glyma.13G072800**	1.90	2.43	4.02	69.9	63.8	35.9	-1.00
**Glyma.13G074700**	2.60	1.59	0.02	25.0	39.6	55.0	-0.99
**Glyma.13G075100**	4.21	3.32	0.07	31.8	50.0	72.7	-1.00
**Glyma.13G077100**	3.99	3.88	2.75	62.6	63.6	93.8	-1.00
**Glyma.13G078500**	1.29	0.20	0	5.0	39.4	27.3	-0.90
**Glyma.13G080500**	5.44	4.38	0.09	2.6	34.0	43.6	-0.97
**Glyma.13G081400**	2.16	1.44	0.40	10.5	30.7	46.7	-1.00
**Glyma.13G083000**	3.05	3.42	0.18	11.5	0	64.3	-1.00
**Glyma.13G094100**	1.54	1.66	0.04	3.1	0	40.9	-1.00
**Glyma.13G097600**	3.59	3.73	4.89	38.6	58.9	11.1	-0.88
**Glyma.13G097800**	2.07	1.34	0	32.1	56.7	71.4	-0.99
**Glyma.13G101500**	3.80	5.89	1.87	58.1	42.6	82.1	-0.89
**Glyma.13G101700**	1.63	0.29	1.28	40.8	81.0	62.1	-0.98
**Glyma.13G107900**	3.13	2.82	1.04	4.6	11.4	64.7	-0.99
**Glyma.13G110800**	4.25	4.30	2.39	0	0	39.1	-1.00
**Glyma.13G112200**	2.92	2.50	0.18	70.0	82.5	100.0	-0.99
**Glyma.13G113000**	1.59	0.19	0	0	34.5	37.9	-1.00
**Glyma.13G114200**	1.26	0.25	4.70E-07	10.5	45.2	50.0	-1.00
**Glyma.13G119500**	0.54	0.68	2.22	68.8	81.3	33.3	-0.96
**Glyma.13G128200**	1.15	0.14	0.04	4.5	34.3	72.7	-0.86
**Glyma.13G129500**	3.68	1.82	0.16	13.9	33.4	44.4	-0.99
**Glyma.13G130800**	1.11	1.74	3.37	79.3	61.0	43.8	-0.92

**Glyma.13G135200**	4.02	1.71	0.25	17.9	50.8	62.5	-0.99
**Glyma.13G144100**	1.65	1.62	0.52	27.6	31.1	60.0	-1.00
**Glyma.13G144600**	0.89	1.38	0.25	23.4	0	33.3	-0.98
**Glyma.13G146600**	2.24	1.60	0.15	6.8	33.2	41.4	-0.95
**Glyma.13G148600**	1.85	2.21	0.65	53.3	48.2	84.6	-0.98
**Glyma.13G150900**	3.49	3.03	1.49	36.2	64.3	75.0	-0.92
**Glyma.13G161600**	3.01	3.24	5.06	42.9	64.3	19.1	-0.86
**Glyma.13G162500**	2.80	2.51	1.30	10.7	35.7	70.0	-0.99
**Glyma.13G165500**	1.30	2.01	2.91	32.4	10.9	0	-0.92
**Glyma.13G165600**	1.18	0.25	0.01	1.9	59.3	44.4	-0.93
**Glyma.13G167600**	3.96	3.88	2.91	15.3	14.9	54.1	-1.00
**Glyma.13G169500**	1.87	0.80	1.52	0	31.7	16.7	-0.99
**Glyma.13G170700**	2.77	2.71	0.49	0	39.5	79.0	-0.89
**Glyma.13G171100**	3.40	1.86	0.43	1.7	33.2	27.3	-0.91
**Glyma.13G172200**	2.87	2.61	4.15	39.6	27.5	5.0	-0.90
**Glyma.13G174400**	1.80	1.10	0.22	0	5.0	42.3	-0.88
**Glyma.13G174700**	1.45	0.89	0.38	4.5	35.3	31.8	-0.88
**Glyma.13G174900**	2.23	1.38	0.03	20.0	36.0	54.2	-0.99
**Glyma.13G180800**	1.32	0.41	2.02	33.5	50.7	15.2	-1.00
**Glyma.13G184000**	1.65	0.77	0.33	56.0	89.0	90.9	-0.98
**Glyma.13G185300**	3.76	2.93	1.80	15.4	50.0	76.0	-1.00
**Glyma.13G198000**	2.72	2.61	1.48	48.3	53.3	83.3	-1.00
**Glyma.13G199300**	3.70	3.18	2.36	1.9	11.5	34.6	-0.97
**Glyma.13G201300**	5.25	7.55	9.90	70.0	75.0	45.0	-0.95
**Glyma.13G201600**	1.42	0.49	0.02	21.8	38.1	61.1	-0.92
**Glyma.13G202100**	2.46	1.10	0.86	19.4	59.7	54.8	-0.98
**Glyma.13G205200**	0.15	0.87	4.77	40.9	29.4	10.0	-0.94
**Glyma.13G205300**	3.40	3.26	0.19	2.0	16.1	53.6	-0.98
**Glyma.13G206500**	4.51	3.20	1.03	13.6	42.5	50.0	-0.99
**Glyma.13G214600**	9.25	7.52	4.74	33.3	64.2	85.7	-0.99
**Glyma.13G220000**	2.98	1.70	1.52	12.5	40.0	45.0	-1.00
**Glyma.13G220600**	3.54	3.85	4.77	39.3	29.3	0	-1.00
**Glyma.13G224900**	3.51	2.82	0.21	0	0	31.3	-0.91
**Glyma.13G229800**	1.03	0.16	0	32.4	82.6	90.9	-1.00
**Glyma.13G231000**	2.20	0.62	0.04	17.1	75.9	85.2	-1.00
**Glyma.13G237100**	1.13	0.28	0	45.6	64.7	75.8	-0.98
**Glyma.13G241100**	2.51	2.46	0.30	0	0	52.9	-1.00
**Glyma.13G241600**	1.40	0.55	0.11	5.0	25.0	40.0	-0.98
**Glyma.13G243400**	3.96	3.76	5.04	67.5	84.4	33.3	-0.97
**Glyma.13G249900**	3.73	3.98	6.53	54.4	75.7	17.4	-0.92
**Glyma.13G251100**	2.30	0.76	0.40	25.9	59.5	78.6	-0.96
**Glyma.13G255200**	1.29	1.87	0.54	14.5	6.3	36.8	-0.95
**Glyma.13G257100**	1.86	2.17	0.39	73.3	64.2	94.4	-1.00

**Glyma.13G257200**	1.71	1.12	0.48	31.3	57.2	73.9	-1.00
**Glyma.13G260800**	3.46	0.50	0	10.0	49.1	61.5	-0.98
**Glyma.13G266300**	1.10	0.62	0.03	6.3	43.8	66.7	-1.00
**Glyma.13G266500**	2.67	2.42	0.10	0	1.7	40.6	-0.99
**Glyma.13G266700**	2.98	3.04	4.25	59.1	72.1	40.9	-0.90
**Glyma.13G268500**	4.03	3.88	0.98	34.0	58.3	79.2	-0.89
**Glyma.13G270900**	1.96	1.27	0.06	0	0	53.1	-0.86
**Glyma.13G273000**	4.53	3.68	0.18	45.6	76.7	81.3	-0.90
**Glyma.13G275400**	3.43	3.97	1.87	31.9	34.9	90.0	-0.89
**Glyma.13G275800**	1.08	0.58	0.04	9.5	13.1	50.0	-0.86
**Glyma.13G276600**	1.11	1.33	0.05	22.2	5.6	50.0	-0.99
**Glyma.13G276800**	2.16	1.53	0.17	50.0	68.5	81.0	-0.99
**Glyma.13G282000**	7.60	5.40	0.94	39.0	69.4	76.9	-1.00
**Glyma.13G285800**	2.17	1.02	0.01	19.9	59.9	72.7	-1.00
**Glyma.13G289000**	1.79	0.94	0	47.5	94.1	100.0	-0.96
**Glyma.13G289100**	3.64	2.65	0.48	28.6	68.1	73.1	-0.94
**Glyma.13G289800**	3.71	3.72	1.93	0	2.5	31.3	-1.00
**Glyma.13G290900**	6.50	5.94	5.08	8.7	25.9	48.3	-1.00
**Glyma.13G301000**	2.54	2.70	3.59	52.2	64.0	11.8	-0.95
**Glyma.13G303000**	1.64	0.68	0	0	52.5	58.3	-0.98
**Glyma.13G304100**	2.89	1.68	0.01	51.5	68.9	83.3	-0.99
**Glyma.13G308800**	1.56	0.72	0.06	6.3	49.3	77.8	-1.00
**Glyma.13G310700**	2.05	0.25	0.01	11.8	38.2	47.1	-0.98
**Glyma.13G314800**	2.91	2.50	0.91	19.2	33.0	57.7	-1.00
**Glyma.13G317000**	4.63	5.43	0.32	55.5	55.4	89.3	-0.90
**Glyma.13G323500**	2.81	2.82	1.75	0	33.6	100.0	-0.94
**Glyma.13G333000**	4.44	4.26	2.81	19.4	26.1	56.3	-1.00
**Glyma.13G334300**	6.23	4.99	3.50	18.2	42.0	50.0	-1.00
**Glyma.13G337900**	1.98	1.11	0.08	5.3	21.1	36.8	-0.99
**Glyma.13G342300**	4.20	4.11	0.13	1.4	0	32.3	-1.00
**Glyma.13G342800**	4.48	4.02	2.92	42.3	71.4	80.0	-0.92
**Glyma.13G344200**	3.08	2.61	0.17	0	0	30.4	-0.95
**Glyma.13G347800**	2.04	0.27	0.02	58.3	94.7	84.2	-0.94
**Glyma.13G350700**	1.23	0.59	0.15	65.1	95.8	100.0	-0.98
**Glyma.13G359900**	2.90	2.90	0.27	0	0	56.0	-1.00
**Glyma.13G360000**	1.16	0.22	0	26.0	54.5	68.0	-0.98
**Glyma.13G362100**	2.26	0.62	0.03	0	37.2	25.0	-0.90
**Glyma.13G363500**	3.41	3.13	0.54	61.8	68.2	94.1	-1.00
**Glyma.13G365500**	3.24	1.26	1.05	39.3	81.3	83.3	-1.00
**Glyma.13G365800**	6.45	8.77	10.05	83.3	76.0	42.3	-0.98
**Glyma.13G366100**	4.54	4.50	5.92	42.9	24.7	6.3	-0.86
**Glyma.13G367900**	4.10	3.56	1.65	19.6	26.8	53.6	-0.98
**Glyma.13G371400**	7.28	6.40	1.47	46.3	65.5	77.8	-0.99

**Glyma.14G003400**	6.72	5.55	1.13	2.4	18.6	52.4	-0.96
**Glyma.14G007800**	2.78	2.85	0.11	3.3	0	30.0	-1.00
**Glyma.14G010700**	4.52	4.14	1.33	0	0	64.7	-0.97
**Glyma.14G017800**	3.62	4.05	5.83	63.2	60.5	31.6	-1.00
**Glyma.14G023600**	4.79	3.89	2.14	14.3	43.8	64.3	-1.00
**Glyma.14G025900**	4.20	3.62	2.89	15.4	48.1	61.5	-0.99
**Glyma.14G026400**	0.32	0.90	3.44	42.1	59.3	9.4	-0.92
**Glyma.14G031200**	2.86	2.09	0.32	16.0	70.0	80.0	-0.93
**Glyma.14G033000**	6.58	1.56	0.57	18.8	59.7	66.7	-0.99
**Glyma.14G033500**	2.56	1.93	0.73	17.7	40.3	74.2	-0.99
**Glyma.14G040600**	6.77	6.54	5.69	53.6	66.7	85.0	-0.99
**Glyma.14G044000**	4.07	4.24	5.35	51.9	59.4	12.5	-0.97
**Glyma.14G047700**	1.06	0.77	0.04	0	2.9	58.8	-0.95
**Glyma.14G048000**	2.04	1.60	0.13	22.6	38.9	70.8	-1.00
**Glyma.14G053900**	1.59	0.78	0	13.5	48.3	52.6	-0.97
**Glyma.14G061900**	4.88	3.94	2.13	6.8	45.1	51.6	-0.95
**Glyma.14G063800**	1.68	3.82	6.44	43.3	67.5	10.0	-0.85
**Glyma.14G063900**	4.00	3.33	1.10	23.8	44.9	65.0	-1.00
**Glyma.14G064000**	1.90	0.32	4.48E-04	9.0	45.8	36.4	-0.95
**Glyma.14G068100**	1.04	0.99	0.03	0	2.0	71.4	-1.00
**Glyma.14G074200**	1.82	1.48	0.18	48.1	61.9	85.7	-1.00
**Glyma.14G074300**	1.49	0.93	0.17	40.9	82.6	83.3	-0.90
**Glyma.14G074400**	1.49	1.56	0.23	5.8	3.1	56.5	-1.00
**Glyma.14G079100**	4.03	1.98	0	29.2	54.5	68.2	-0.99
**Glyma.14G081700**	5.24	4.84	4.15	12.1	43.1	48.3	-0.90
**Glyma.14G084400**	1.60	1.41	0.40	14.8	15.7	46.4	-0.98
**Glyma.14G085200**	1.45	1.08	0.05	29.2	43.2	60.0	-0.99
**Glyma.14G086200**	1.51	0.32	0	31.7	55.7	91.7	-0.87
**Glyma.14G086900**	2.06	2.38	1.28	2.1	0	58.1	-0.94
**Glyma.14G087100**	2.24	1.18	0.99	4.1	27.4	48.4	-0.92
**Glyma.14G087400**	0.61	0.76	2.67	40.0	54.3	23.5	-0.87
**Glyma.14G088700**	0.76	0.86	2.44	68.3	64.6	19.1	-1.00
**Glyma.14G091500**	1.62	1.12	0.05	50.0	67.6	92.0	-1.00
**Glyma.14G092500**	4.60	3.37	0.15	19.8	49.0	54.6	-0.97
**Glyma.14G095300**	2.12	1.49	0.20	37.1	83.7	83.3	-0.85
**Glyma.14G098600**	4.71	4.65	0.45	0	2.9	31.3	-1.00
**Glyma.14G099200**	2.01	1.50	0.03	3.2	21.0	46.9	-1.00
**Glyma.14G102000**	3.62	2.66	0.29	4.7	25.0	43.8	-1.00
**Glyma.14G103700**	2.75	2.95	1.40	0	0	35.7	-0.98
**Glyma.14G104600**	3.12	3.26	2.12	33.3	4.9	60.0	-0.93
**Glyma.14G105800**	1.62	0.88	0.51	3.1	39.1	47.6	-1.00
**Glyma.14G106700**	0.87	2.45	4.71	39.3	38.1	8.3	-0.99
**Glyma.14G112400**	2.68	2.36	1.05	10.0	18.0	48.0	-1.00

**Glyma.14G115500**	5.53	2.75	0.34	35.3	91.2	100.0	-1.00
**Glyma.14G116100**	1.09	0.55	4.88E-03	11.8	29.4	64.7	-0.96
**Glyma.14G116500**	0.04	0.41	2.63	23.6	35.0	5.0	-0.91
**Glyma.14G117100**	2.10	2.65	4.61	50.7	42.9	8.7	-1.00
**Glyma.14G117500**	2.61	1.65	1.22	5.2	43.1	51.7	-1.00
**Glyma.14G118200**	0.30	0.65	2.71	45.0	40.0	9.1	-1.00
**Glyma.14G122200**	2.91	2.97	1.69	7.3	12.0	90.0	-0.99
**Glyma.14G122600**	1.36	0.92	0.06	39.2	53.0	75.8	-1.00
**Glyma.14G124300**	1.94	1.55	0.55	19.6	41.1	67.9	-1.00
**Glyma.14G128900**	1.44	0.99	0.14	28.5	43.5	62.1	-1.00
**Glyma.14G130000**	1.46	1.49	0.16	3.8	15.2	40.0	-0.94
**Glyma.14G130800**	1.85	1.95	0.84	0	0	51.7	-0.99
**Glyma.14G131300**	3.84	3.50	1.41	8.3	23.3	63.6	-1.00
**Glyma.14G133000**	1.29	0.17	3.70E-04	17.6	39.6	56.5	-0.93
**Glyma.14G133600**	2.63	2.34	0.94	9.6	19.4	45.8	-1.00
**Glyma.14G135400**	2.40	1.57	0.12	17.5	30.9	52.9	-0.98
**Glyma.14G138200**	3.12	2.93	0.16	2.9	5.9	35.3	-1.00
**Glyma.14G146400**	1.13	1.11	0.06	13.4	5.9	50.0	-0.98
**Glyma.14G147200**	2.58	2.31	0.61	22.5	35.9	80.0	-1.00
**Glyma.14G150900**	1.10	0.23	2.28E-05	26.8	65.6	66.7	-0.99
**Glyma.14G154200**	1.75	0.11	6.10E-05	7.6	43.0	34.8	-0.97
**Glyma.14G156500**	2.58	3.04	1.96	42.6	14.3	93.8	-0.98
**Glyma.14G158500**	3.16	1.82	2.35	12.5	69.8	56.3	-1.00
**Glyma.14G158800**	1.92	1.26	0.02	2.0	4.9	40.0	-0.90
**Glyma.14G160600**	1.84	2.22	0.52	36.0	44.0	84.0	-0.88
**Glyma.14G166500**	1.41	1.41	0.16	11.3	29.2	70.8	-0.96
**Glyma.14G170900**	4.81	2.49	0.52	33.9	58.9	72.0	-0.98
**Glyma.14G174200**	0.54	0.63	2.17	49.1	47.8	13.0	-1.00
**Glyma.14G174600**	1.62	0.36	0.02	5.6	35.2	56.5	-0.95
**Glyma.14G176600**	4.40	4.54	2.29	43.2	43.2	100.0	-0.99
**Glyma.14G176900**	5.89	5.02	3.06	39.7	67.2	86.2	-1.00
**Glyma.14G177200**	4.09	1.98	0.76	8.3	71.1	68.4	-0.98
**Glyma.14G178000**	3.13	1.95	0.04	14.7	40.0	52.9	-1.00
**Glyma.14G179000**	3.35	3.15	0.83	3.8	25.0	42.3	-0.91
**Glyma.14G179600**	3.38	1.67	1.33	0	56.7	73.3	-0.99
**Glyma.14G182300**	2.22	0.75	0.03	38.9	72.2	94.4	-0.97
**Glyma.14G182900**	1.79	1.10	0.35	34.5	69.0	86.2	-1.00
**Glyma.14G192700**	1.39	1.08	0.08	62.1	85.8	94.7	-0.90
**Glyma.14G192900**	2.06	2.29	3.70	48.7	46.2	15.4	-1.00
**Glyma.14G193100**	3.08	1.01	0.20	4.5	63.6	80.0	-1.00
**Glyma.14G194500**	4.38	4.93	3.20	0	0	35.3	-0.89
**Glyma.14G197400**	1.19	1.90	2.19	35.1	0	0	-0.93
**Glyma.14G197600**	3.39	2.78	1.31	12.5	42.2	71.9	-1.00

**Glyma.14G209700**	3.01	1.11	0.38	60.6	83.3	91.7	-0.99
**Glyma.14G216200**	2.71	1.55	0.06	16.1	44.4	70.3	-0.99
**Glyma.14G217000**	1.86	2.12	0.35	0	0	45.5	-0.98
**Glyma.14G218800**	1.92	1.66	0.20	13.9	4.2	38.9	-0.87
**Glyma.14G221100**	2.41	2.08	0.70	8.5	27.4	48.3	-0.98
**Glyma.15G001500**	2.33	1.93	0.10	7.1	27.1	70.8	-1.00
**Glyma.15G003400**	4.19	3.27	1.65	34.2	60.5	68.4	-0.98
**Glyma.15G003600**	4.94	4.82	2.39	24.0	50.0	80.0	-0.92
**Glyma.15G007700**	4.48	2.32	2.92	22.6	73.3	54.6	-0.97
**Glyma.15G012400**	1.04	1.99	1.02E-04	52.0	10.5	68.4	-1.00
**Glyma.15G020500**	1.84	0	0	20.0	69.1	71.4	-1.00
**Glyma.15G022300**	3.46	3.15	1.27	40.9	56.4	71.4	-0.96
**Glyma.15G034300**	1.53	0.80	0.07	37.3	78.5	95.2	-1.00
**Glyma.15G038700**	1.84	0.90	0.06	13.9	59.4	87.5	-1.00
**Glyma.15G039100**	3.45	3.08	0.17	60.3	73.5	94.1	-0.99
**Glyma.15G040300**	5.99	4.88	2.02	53.4	82.0	96.0	-0.99
**Glyma.15G042100**	5.90	4.67	2.63	30.4	41.1	60.7	-0.95
**Glyma.15G045400**	2.96	1.96	0.02	32.8	61.0	72.7	-0.99
**Glyma.15G051700**	4.37	4.30	1.82	0	5.0	100.0	-1.00
**Glyma.15G055600**	2.49	2.02	0.47	44.4	66.3	100.0	-1.00
**Glyma.15G061600**	4.75	3.55	2.04	50.0	80.0	93.3	-1.00
**Glyma.15G061700**	1.59	0.24	0	63.4	95.2	100.0	-1.00
**Glyma.15G065700**	2.62	0.78	0.06	34.4	50.8	73.1	-0.88
**Glyma.15G067000**	0.46	0.27	1.56	32.4	56.7	11.1	-0.89
**Glyma.15G067700**	1.84	1.93	0.22	12.5	11.1	83.3	-1.00
**Glyma.15G071800**	1.81	2.06	0.72	0	0	73.7	-0.97
**Glyma.15G074800**	2.02	0.30	0.04	45.4	86.7	78.6	-0.97
**Glyma.15G081500**	2.38	1.85	0.05	25.6	45.1	63.4	-0.99
**Glyma.15G083500**	1.54	0.39	1.14E-04	8.8	32.7	54.6	-0.94
**Glyma.15G089000**	3.74	0.89	1.89E-05	42.9	82.1	87.5	-1.00
**Glyma.15G099300**	1.51	2.73E-04	2.01E-06	16.1	61.8	75.0	-0.98
**Glyma.15G104400**	3.01	3.49	0.95	60.9	50.0	82.6	-1.00
**Glyma.15G106200**	1.84	1.94	0.11	0	0	33.3	-1.00
**Glyma.15G114100**	2.48	0.62	0.03	1.6	24.7	33.3	-0.99
**Glyma.15G123000**	4.21	3.99	2.83	0	0	72.7	-0.98
**Glyma.15G123200**	1.06	0.40	0	25.1	65.6	81.8	-1.00
**Glyma.15G126600**	2.86	2.64	0.74	0	40.5	66.7	-0.89
**Glyma.15G128300**	1.30	0.79	0.03	7.1	41.3	44.4	-0.90
**Glyma.15G128400**	3.82	4.80	6.42	30.0	42.7	5.9	-0.86
**Glyma.15G133900**	2.08	0.62	0.01	50.0	77.3	81.8	-1.00
**Glyma.15G135200**	3.90	3.07	0.57	8.9	23.2	46.4	-0.99
**Glyma.15G135800**	4.36	4.64	3.49	58.3	40.0	80.0	-0.99
**Glyma.15G136500**	1.51	0.78	0.12	11.4	34.3	51.4	-1.00

**Glyma.15G140000**	3.40	3.07	2.10	19.8	28.7	56.0	-0.99
**Glyma.15G140300**	3.56	3.11	1.86	0	17.5	34.6	-0.99
**Glyma.15G143100**	4.90	3.39	0.57	41.6	73.5	82.4	-0.99
**Glyma.15G147700**	8.09	7.64	6.89	7.8	27.3	45.5	-1.00
**Glyma.15G148300**	3.40	1.07	0.02	2.5	39.0	36.8	-0.99
**Glyma.15G149200**	1.85	0.42	0.12	45.3	80.1	83.3	-1.00
**Glyma.15G155600**	2.08	1.22	0.03	1.9	52.0	86.4	-1.00
**Glyma.15G156500**	2.14	1.82	3.41	54.9	89.5	0	-0.96
**Glyma.15G157800**	2.68	2.28	0.93	17.9	24.7	68.4	-0.98
**Glyma.15G162600**	4.04	2.21	0.11	43.2	89.0	93.3	-0.99
**Glyma.15G167900**	3.74	3.10	0.70	16.0	42.1	79.0	-1.00
**Glyma.15G168000**	1.89	1.95	0.40	6.7	9.4	53.9	-0.99
**Glyma.15G170900**	2.32	1.09	0.03	26.7	82.1	100.0	-1.00
**Glyma.15G172200**	1.56	0.68	9.61E-04	1.9	22.6	33.3	-1.00
**Glyma.15G173300**	4.71	2.76	0.08	20.6	48.8	72.2	-0.97
**Glyma.15G183000**	2.67	1.18	0.02	5.6	25.9	37.0	-0.99
**Glyma.15G184400**	2.40	1.60	0.16	16.1	33.8	54.6	-1.00
**Glyma.15G188900**	2.84	2.35	1.47	31.7	71.7	79.0	-0.91
**Glyma.15G192100**	3.08	1.63	0.04	13.5	39.2	45.5	-1.00
**Glyma.15G193300**	2.48	1.74	1.07	0	7.1	33.3	-0.89
**Glyma.15G193400**	2.33	2.38	1.27	10.0	0	70.0	-1.00
**Glyma.15G194300**	5.66	4.26	0.28	20.2	43.9	54.6	-1.00
**Glyma.15G197200**	4.70	4.12	1.21	40.0	56.3	70.0	-0.98
**Glyma.15G199500**	2.36	1.69	0.51	16.0	29.3	46.3	-1.00
**Glyma.15G201100**	1.93	3.43	4.41	73.9	78.5	36.8	-0.87
**Glyma.15G201700**	2.78	0.46	0.05	31.0	71.4	66.7	-0.99
**Glyma.15G202800**	4.77	4.22	3.12	0	14.0	30.4	-1.00
**Glyma.15G203000**	3.23	3.30	0.40	8.9	7.1	45.5	-1.00
**Glyma.15G203600**	2.77	1.70	0.47	5.2	13.1	37.9	-0.89
**Glyma.15G208600**	2.42	2.51	0.06	7.1	21.4	38.9	-0.86
**Glyma.15G210300**	2.49	1.45	3.45E-05	2.2	31.2	42.9	-1.00
**Glyma.15G211700**	3.77	3.11	0.16	13.8	35.2	76.9	-1.00
**Glyma.15G211800**	2.24	1.31	0.46	9.0	51.2	65.6	-1.00
**Glyma.15G212200**	2.47	2.16	3.67	27.9	48.5	8.8	-0.91
**Glyma.15G213400**	1.87	2.27	3.36	35.9	47.4	17.2	-0.85
**Glyma.15G213700**	0.38	1.29	0.11	76.4	63.1	94.4	-0.90
**Glyma.15G215600**	2.19	1.60	3.12E-05	2.8	19.4	60.0	-0.99
**Glyma.15G217100**	2.49	0.48	0.02	17.9	52.6	60.0	-1.00
**Glyma.15G217500**	7.89	7.00	2.85	9.5	24.1	41.9	-1.00
**Glyma.15G219100**	1.34	1.14	0.08	0	0	31.6	-0.98
**Glyma.15G219400**	2.70	2.57	3.56E-05	4.5	12.3	38.1	-0.99
**Glyma.15G221300**	5.18	4.05	0.81	19.4	25.0	88.9	-0.86
**Glyma.15G221600**	4.32	3.24	0.07	28.8	52.2	73.1	-1.00

**Glyma.15G222400**	4.12	4.51	0.35	5.9	10.0	53.3	-0.95
**Glyma.15G223100**	2.70	2.23	1.26	0	0	70.0	-0.90
**Glyma.15G227600**	5.50	4.43	4.26	10.8	40.9	43.3	-1.00
**Glyma.15G230600**	2.60	2.46	0.47	3.6	10.7	35.7	-0.99
**Glyma.15G234500**	2.03	1.50	1.01	44.5	57.3	76.2	-0.97
**Glyma.15G235900**	1.81	1.49	0.01	15.6	23.3	64.4	-0.99
**Glyma.15G238200**	3.49	1.53	0.47	9.6	48.8	57.1	-1.00
**Glyma.15G243000**	3.38	1.52	0.02	31.6	58.1	66.7	-1.00
**Glyma.15G252800**	1.20	0.10	1.98E-05	32.8	77.6	82.8	-1.00
**Glyma.15G253000**	2.83	2.93	3.91	34.6	19.2	4.6	-0.89
**Glyma.15G253500**	6.38E-03	5.84E-05	3.93	17.9	32.6	0	-0.89
**Glyma.15G253700**	9.01	7.78	4.69	50.0	79.2	91.7	-0.99
**Glyma.15G254400**	3.41	3.02	4.27	18.3	35.0	0	-0.96
**Glyma.15G258800**	1.38	3.01	0.73	20.0	3.3	46.2	-0.87
**Glyma.15G260100**	1.43	1.79	0.43	2.4	0	50.0	-0.95
**Glyma.15G260300**	6.01	6.22	4.27	58.0	43.4	96.0	-1.00
**Glyma.15G262400**	2.01	0.85	0.06	7.4	38.9	41.7	-0.98
**Glyma.15G262500**	1.05	0.16	6.75E-05	20.0	44.0	54.2	-0.98
**Glyma.15G263500**	4.98	4.24	2.42	17.7	54.1	56.7	-0.89
**Glyma.15G266900**	2.05	1.75	1.04	15.9	25.6	50.0	-1.00
**Glyma.15G275000**	2.51	3.31	0.79	52.0	28.7	79.0	-1.00
**Glyma.15G275300**	1.69	1.10	0.27	3.5	19.2	37.9	-1.00
**Glyma.15G275600**	5.88	4.58	0.28	10.1	36.5	50.0	-1.00
**Glyma.16G000200**	1.80	1.62	0.20	0	7.1	52.6	-1.00
**Glyma.16G007700**	1.31	0.72	1.75E-05	38.5	73.3	80.0	-0.95
**Glyma.16G015300**	2.13	1.70	3.72E-06	0	0	46.0	-0.95
**Glyma.16G015700**	3.57	3.79	4.97	68.2	63.6	27.3	-1.00
**Glyma.16G017700**	3.86	2.93	0.27	19.0	60.8	77.8	-0.98
**Glyma.16G019700**	3.20	3.23	0.51	24.1	49.4	86.4	-0.90
**Glyma.16G023500**	3.02	1.87	1.44E-05	12.9	30.3	47.6	-0.99
**Glyma.16G026700**	2.22	1.71	1.18	0	32.3	83.3	-0.98
**Glyma.16G026900**	0.16	0.32	5.53	31.4	47.6	13.3	-0.88
**Glyma.16G031100**	0.91	1.14	0.08	0	0	50.0	-0.96
**Glyma.16G035000**	2.63	3.27	5.72	55.4	70.8	30.8	-0.90
**Glyma.16G043700**	3.17	2.90	4.04E-03	39.6	60.2	100.0	-0.99
**Glyma.16G046800**	1.40	1.96	0.07	0	0	62.1	-0.90
**Glyma.16G047300**	1.63	1.21	0.49	46.3	61.1	85.2	-1.00
**Glyma.16G047800**	2.29	1.98	1.18	2.3	7.8	50.0	-0.97
**Glyma.16G048100**	2.73	3.09	0.38	34.8	47.8	78.3	-0.85
**Glyma.16G049300**	3.62	3.28	0.53	24.6	15.1	72.7	-0.93
**Glyma.16G049700**	1.93	2.24	0.04	9.4	15.0	41.2	-0.92
**Glyma.16G050200**	4.41	4.23	3.24	38.1	52.4	71.4	-0.97
**Glyma.16G050500**	4.53	4.26	3.05	0	0	45.5	-0.96

**Glyma.16G050800**	8.01	6.81	4.07	14.1	37.5	53.3	-1.00
**Glyma.16G052100**	2.40	0.07	0.06	48.2	75.0	85.7	-0.96
**Glyma.16G053200**	4.94	4.26	0.27	7.8	17.6	43.5	-0.99
**Glyma.16G053900**	1.79	2.97	4.43	35.0	22.7	0	-0.99
**Glyma.16G058500**	1.20	2.00	3.60	52.8	58.1	23.5	-0.95
**Glyma.16G059700**	2.44	1.54	0.05	18.4	33.1	53.3	-0.99
**Glyma.16G060300**	2.53	0.23	0.02	8.0	24.7	40.0	-0.89
**Glyma.16G060400**	1.34	0.30	0.15	38.5	63.5	69.6	-0.99
**Glyma.16G062700**	1.47	0.37	2.32E-06	42.0	90.9	100.0	-1.00
**Glyma.16G071800**	12.78	13.07	10.98	0	0	40.0	-0.97
**Glyma.16G075100**	1.07	0	0.03	10.0	51.3	36.4	-0.94
**Glyma.16G076700**	1.95	1.70	0.70	0	0	52.6	-0.96
**Glyma.16G077800**	3.25	2.43	2.11	44.7	75.6	79.0	-0.99
**Glyma.16G077900**	1.11	1.29	3.44	32.8	41.7	10.0	-0.95
**Glyma.16G078300**	2.00	1.30	0.04	3.7	21.5	41.5	-1.00
**Glyma.16G082400**	3.07	2.52	2.00	5.3	36.4	71.4	-0.99
**Glyma.16G082900**	1.58	0.85	0.12	10.4	23.4	61.5	-0.91
**Glyma.16G083300**	3.37	2.90	1.53	0	22.0	33.3	-0.95
**Glyma.16G090800**	1.40	0	0	2.0	19.0	36.0	-0.87
**Glyma.16G093600**	3.41	3.26	2.22	0	6.5	38.5	-1.00
**Glyma.16G094400**	1.18	0.90	0.01	28.0	36.0	60.0	-1.00
**Glyma.16G096600**	1.64	1.07	3.31	36.1	31.2	0	-0.97
**Glyma.16G101800**	1.15	0.61	2.34E-06	27.0	50.0	64.9	-1.00
**Glyma.16G102400**	3.53	3.65	2.16	50.0	24.7	65.4	-0.86
**Glyma.16G102500**	1.72	1.35	0.44	16.7	33.3	53.3	-1.00
**Glyma.16G104000**	1.76	1.66	0.12	9.1	21.7	41.2	-0.95
**Glyma.16G107700**	2.23	1.81	0.08	5.9	13.5	42.3	-0.99
**Glyma.16G110100**	3.86E-03	1.04	3.09	26.5	39.5	6.7	-0.86
**Glyma.16G111200**	2.21	1.94	0.17	5.6	17.4	58.8	-1.00
**Glyma.16G111900**	2.11	1.68	0.06	3.2	23.6	67.7	-1.00
**Glyma.16G115500**	1.07	0.13	0.02	5.6	30.9	39.3	-0.98
**Glyma.16G118900**	2.63	1.89	0.97	13.7	33.5	54.8	-0.99
**Glyma.16G120200**	2.76	1.92	1.22	13.6	32.5	44.8	-1.00
**Glyma.16G129200**	0.68	0.12	1.21	32.4	57.5	16.7	-0.97
**Glyma.16G130900**	3.28	4.13	6.73	55.0	76.7	10.0	-0.92
**Glyma.16G131600**	2.36	2.24	3.28	48.7	69.9	17.7	-0.94
**Glyma.16G131800**	4.26	3.92	0.22	18.8	43.8	100.0	-1.00
**Glyma.16G131900**	2.50	2.78	4.33	29.8	31.7	0	-0.99
**Glyma.16G133100**	0.15	0.41	1.41	59.3	68.6	25.0	-0.94
**Glyma.16G138100**	2.84	2.76	1.64	35.5	35.5	74.2	-1.00
**Glyma.16G138200**	1.66	1.69	0.47	27.5	32.5	73.1	-0.99
**Glyma.16G141200**	0	7.55E-05	2.52	46.1	38.4	13.8	-0.97
**Glyma.16G145600**	0.01	0.02	4.30	25.0	43.2	8.3	-0.85

**Glyma.16G146000**	3.22	4.12	4.49	33.3	10.0	0	-0.99
**Glyma.16G147200**	2.28	1.65	0.08	4.4	19.6	35.7	-1.00
**Glyma.16G147600**	6.20	5.50	4.94	21.7	38.9	52.2	-0.99
**Glyma.16G151200**	3.89	1.27	0.04	21.1	82.5	87.5	-1.00
**Glyma.16G151700**	2.92	2.96	5.05	81.3	90.6	56.3	-0.96
**Glyma.16G153200**	5.52	4.81	4.50	13.6	60.4	60.0	-0.97
**Glyma.16G165600**	1.48	0.32	0.41	33.7	67.5	52.0	-0.91
**Glyma.16G172600**	1.54	0.43	5.14E-05	36.3	71.0	87.5	-0.99
**Glyma.16G176100**	0.21	0.10	1.32	53.3	70.0	30.0	-0.93
**Glyma.16G179500**	3.07	3.03	1.52	44.4	34.0	72.7	-0.96
**Glyma.16G179900**	2.72	2.31	1.25	10.0	35.0	76.7	-1.00
**Glyma.16G180500**	1.83	1.37	0.20	16.7	55.8	69.2	-0.92
**Glyma.16G181900**	1.81	0.20	0.10	30.9	77.0	90.9	-0.98
**Glyma.16G182100**	1.74	1.50	0.57	0	13.6	38.9	-1.00
**Glyma.16G200400**	3.55	2.64	1.91	20.3	76.6	90.6	-0.99
**Glyma.16G203400**	2.34	1.48	0.94	26.5	72.8	70.6	-0.95
**Glyma.17G000300**	1.97	1.90	0.58	17.3	24.5	75.0	-1.00
**Glyma.17G001400**	2.13	2.05	0.16	8.8	26.7	65.5	-0.97
**Glyma.17G002100**	2.46	2.05	1.34	45.1	88.5	90.9	-0.86
**Glyma.17G004300**	6.05	5.02	4.94	18.8	70.5	55.0	-0.94
**Glyma.17G009500**	2.31	2.42	1.09	34.6	19.1	61.8	-0.97
**Glyma.17G009900**	3.89	5.00	3.02	13.1	5.3	35.3	-0.87
**Glyma.17G013800**	5.52	5.59	2.13	2.3	16.2	58.3	-0.96
**Glyma.17G014900**	1.87	0.49	0.61	5.9	37.8	50.0	-0.95
**Glyma.17G016600**	1.21	0.99	0.09	0	0	36.8	-0.97
**Glyma.17G027600**	4.03	3.71	0.47	0	21.4	65.0	-0.99
**Glyma.17G027800**	3.52	3.78	0.69	0	0	33.3	-0.98
**Glyma.17G032300**	3.67	2.40	0.98	6.5	26.1	73.9	-0.90
**Glyma.17G033400**	2.60	1.85	0.01	0	0	42.9	-0.87
**Glyma.17G033700**	1.78	1.13	0.49	20.0	49.4	55.6	-0.97
**Glyma.17G037400**	1.41	1.02	0.34	6.3	54.8	66.7	-0.93
**Glyma.17G038700**	1.27	0.24	0.02	6.0	41.5	50.0	-1.00
**Glyma.17G038800**	2.41	3.00	0.21	0	0	41.2	-0.92
**Glyma.17G041100**	3.07	2.14	0.82	29.2	55.6	69.2	-1.00
**Glyma.17G043500**	5.59	5.77	4.64	2.2	14.0	50.0	-0.90
**Glyma.17G047000**	1.30	1.17	0.23	26.7	35.3	72.7	-1.00
**Glyma.17G048600**	2.67	2.18	0.15	47.0	57.1	82.1	-1.00
**Glyma.17G049800**	4.12	0.77	0.13	53.4	76.1	87.0	-0.96
**Glyma.17G056200**	2.26	2.47	0.76	35.7	45.6	94.1	-0.94
**Glyma.17G057800**	1.05	1.52E-03	0	62.5	95.8	97.2	-1.00
**Glyma.17G061900**	1.86	1.70	0.12	0	0	50.0	-0.99
**Glyma.17G062600**	1.22	0.51	2.03	48.5	39.7	0	-0.87
**Glyma.17G066400**	3.69	3.67	1.63	0	0	44.4	-1.00

**Glyma.17G066900**	3.48	2.87	0.25	40.6	67.3	76.5	-0.92
**Glyma.17G071200**	4.97	3.98	5.06	30.6	81.6	50.0	-0.88
**Glyma.17G073900**	3.07	2.38	0.56	0	0	37.5	-0.89
**Glyma.17G078900**	1.55	0.49	0.06	2.2	32.5	46.7	-0.99
**Glyma.17G080600**	2.01	2.75	0.16	1.7	0	57.1	-0.89
**Glyma.17G081700**	1.27	1.15	0.04	0	0	60.0	-0.99
**Glyma.17G083100**	2.82	2.79	1.42	20.0	26.2	77.3	-1.00
**Glyma.17G084700**	3.93	3.80	4.88	85.2	94.4	51.9	-0.99
**Glyma.17G087100**	1.76	1.97	0.26	0	0	40.0	-0.98
**Glyma.17G089700**	2.39	4.63	6.24	77.1	91.3	41.7	-0.85
**Glyma.17G092400**	1.17	1.02	3.22	79.2	92.2	50.0	-0.96
**Glyma.17G096400**	0.15	0.13	1.90	76.4	81.8	36.4	-0.99
**Glyma.17G096500**	1.81	0.39	0.69	45.3	87.0	82.6	-1.00
**Glyma.17G096700**	4.16	2.86	3.81	22.6	58.1	25.8	-0.96
**Glyma.17G098300**	1.22	0.68	2.64E-05	47.9	60.4	79.2	-0.99
**Glyma.17G098700**	2.19	1.52	0	33.3	53.0	81.8	-1.00
**Glyma.17G103400**	0.15	0.24	1.81	69.9	96.6	6.5	-0.95
**Glyma.17G104000**	2.69	1.60	0.01	58.6	83.7	90.0	-0.98
**Glyma.17G107800**	3.43	0.86	0.04	0	39.3	45.5	-1.00
**Glyma.17G111100**	9.28	8.94	3.53	42.3	60.7	76.9	-0.94
**Glyma.17G112200**	1.38	1.39	0.13	0	0	41.2	-1.00
**Glyma.17G115700**	2.05	0.55	3.40E-04	21.9	53.4	50.0	-0.97
**Glyma.17G120200**	2.84	2.41	1.14	13.8	13.9	75.0	-0.93
**Glyma.17G120400**	1.72	1.39	0.26	59.1	69.6	91.3	-1.00
**Glyma.17G123400**	2.40	0.45	0.01	14.6	65.2	100.0	-0.94
**Glyma.17G126500**	1.04	0.25	0	0	17.4	35.0	-0.94
**Glyma.17G128300**	4.48	3.86	1.17	0	17.9	53.3	-1.00
**Glyma.17G128600**	4.21	3.69	0.71	0	3.8	33.3	-0.97
**Glyma.17G130200**	4.28	3.57	0.77	2.5	12.3	72.7	-0.95
**Glyma.17G131500**	4.69	3.98	3.29	12.5	29.2	66.7	-0.94
**Glyma.17G135900**	2.35	0.69	0.01	10.3	25.9	41.4	-0.93
**Glyma.17G138300**	2.91	1.21	0.07	26.1	78.3	78.3	-0.98
**Glyma.17G139500**	2.89	0.65	0.02	52.5	87.2	95.0	-1.00
**Glyma.17G141100**	5.92	5.13	4.28	19.5	46.3	78.1	-0.98
**Glyma.17G143000**	3.21	3.22	2.13	0	0	37.0	-1.00
**Glyma.17G145400**	0.78	0.74	2.33	46.4	57.2	21.4	-0.96
**Glyma.17G147900**	4.10	3.28	2.70	0	18.8	35.7	-0.98
**Glyma.17G148400**	4.30	2.76	0.27	0	29.2	33.3	-0.98
**Glyma.17G149300**	1.58	0.62	0.18	8.3	52.5	57.1	-0.99
**Glyma.17G151800**	1.25	0.10	0.25	1.9	42.9	66.7	-0.90
**Glyma.17G152800**	2.46	1.72	0.24	19.9	48.9	66.7	-0.99
**Glyma.17G154700**	1.29	1.22	0.28	6.7	27.4	50.0	-0.91
**Glyma.17G154900**	4.53	4.12	2.96	11.4	27.6	50.0	-1.00

**Glyma.17G155000**	6.42	9.04	12.10	38.6	56.3	10.0	-0.89
**Glyma.17G156000**	1.73	1.81	0.01	24.3	50.0	82.4	-0.87
**Glyma.17G157100**	3.79	3.47	1.38	2.1	19.2	45.0	-0.99
**Glyma.17G158500**	1.65	1.97	2.66	75.0	92.9	26.7	-0.88
**Glyma.17G160700**	1.14	0.80	0.02	12.0	43.4	72.0	-0.99
**Glyma.17G161400**	1.66	0.72	0.01	16.6	59.0	69.2	-0.99
**Glyma.17G164100**	5.23	4.58	2.40	6.7	25.0	41.2	-0.99
**Glyma.17G164400**	0.12	0.28	1.95	58.2	43.7	27.3	-0.90
**Glyma.17G165200**	6.29	3.16	0.82	13.5	56.8	63.2	-1.00
**Glyma.17G166400**	5.18	4.42	2.96	4.2	27.9	45.5	-1.00
**Glyma.17G167200**	3.52	5.56	6.98	42.2	40.8	0	-0.96
**Glyma.17G168900**	2.37	2.25	4.42	37.5	65.8	7.1	-0.89
**Glyma.17G169400**	3.38	1.77	2.51	34.9	86.8	68.4	-1.00
**Glyma.17G170200**	1.85	1.65	0.20	14.6	25.3	63.6	-1.00
**Glyma.17G170600**	2.41	2.05	0.14	17.4	58.3	100.0	-0.97
**Glyma.17G171100**	3.40	1.79	2.12	9.6	76.9	65.4	-1.00
**Glyma.17G177600**	2.68	1.82	0.94	29.6	68.8	95.8	-1.00
**Glyma.17G191300**	0.85	1.47	0.02	49.5	37.5	72.7	-0.97
**Glyma.17G194400**	2.05	1.36	2.61	17.9	35.8	3.7	-0.99
**Glyma.17G197900**	1.37	1.11	0.02	0	10.0	42.9	-1.00
**Glyma.17G200500**	2.47	2.24	1.43	0	23.4	47.4	-0.97
**Glyma.17G204800**	3.22	2.70	1.63	7.4	22.1	44.1	-1.00
**Glyma.17G205800**	1.50	0.98	0.05	55.2	87.8	93.8	-0.92
**Glyma.17G206500**	4.25	3.97	3.22	10.0	25.9	54.6	-1.00
**Glyma.17G206600**	4.25	4.29	2.86	0	11.5	42.3	-0.95
**Glyma.17G212200**	2.34	0.54	0.11	15.3	56.9	41.7	-0.90
**Glyma.17G215900**	1.28	0.57	9.54E-07	42.3	63.8	79.3	-1.00
**Glyma.17G218000**	1.70	0.44	0	3.1	51.1	75.0	-0.98
**Glyma.17G219400**	1.14	0.90	0.11	14.4	44.0	91.7	-1.00
**Glyma.17G221200**	4.09	3.80	3.00	0	0	66.7	-0.94
**Glyma.17G222600**	2.06	1.53	0.58	20.3	36.4	51.5	-1.00
**Glyma.17G223200**	2.98	2.53	0.50	12.0	41.4	65.5	-0.97
**Glyma.17G224100**	3.46	3.30	2.12	4.0	14.0	62.5	-1.00
**Glyma.17G227400**	1.80	0.27	0	7.5	32.8	37.9	-1.00
**Glyma.17G230300**	2.34	1.99	1.21	58.3	70.5	90.9	-1.00
**Glyma.17G230700**	3.38	1.47	2.48	25.3	68.4	72.2	-0.90
**Glyma.17G231400**	3.38	2.34	0.94	0	36.0	33.3	-0.91
**Glyma.17G231700**	0.79	0.34	1.46	13.6	47.4	0	-0.90
**Glyma.17G237000**	2.70	2.57	1.26	34.2	48.5	84.9	-0.99
**Glyma.17G237100**	9.29	9.11	3.84	10.9	25.0	66.7	-0.99
**Glyma.17G238800**	3.49	3.25	2.12	0	0	30.0	-0.97
**Glyma.17G242200**	1.71	1.38	0.01	38.3	72.2	91.7	-0.92
**Glyma.17G245900**	3.17	2.75	1.68	13.4	32.5	44.4	-0.97

**Glyma.17G247300**	3.58	2.88	4.36	7.1	42.3	0	-0.86
**Glyma.17G253100**	0.02	1.76E-04	2.08	75.9	71.8	45.8	-0.99
**Glyma.17G257400**	3.47	2.98	0.87	0	1.3	62.9	-0.94
**Glyma.17G258700**	6.42	4.77	4.47	12.5	25.0	42.9	-0.85
**Glyma.18G007600**	3.82	3.69	2.45	37.5	45.5	95.8	-1.00
**Glyma.18G009500**	1.10	0.89	0.01	0	0	39.3	-0.97
**Glyma.18G012500**	1.19	1.28	0.12	32.7	31.0	61.5	-1.00
**Glyma.18G014100**	2.71	2.96	1.15	9.4	0	30.0	-0.99
**Glyma.18G016700**	2.55	2.65	0.68	7.1	10.1	53.9	-0.99
**Glyma.18G019400**	1.13	0.47	2.54E-06	25.0	56.8	63.6	-0.99
**Glyma.18G022800**	2.57	1.98	0.89	30.4	58.7	82.6	-1.00
**Glyma.18G030900**	3.23	1.92	0.05	41.7	100.0	90.0	-0.88
**Glyma.18G031800**	2.90	2.27	0.54	10.5	32.5	52.9	-1.00
**Glyma.18G040000**	5.65	5.43	2.47	29.8	26.8	67.9	-0.98
**Glyma.18G043500**	1.25	0.93	0.10	0	0	50.0	-0.94
**Glyma.18G043700**	0.85	0.27	1.41	33.9	42.7	11.5	-0.99
**Glyma.18G044900**	3.94	1.79	0.15	45.3	95.5	81.8	-0.91
**Glyma.18G045300**	3.46	1.98	1.03	31.0	73.0	81.8	-1.00
**Glyma.18G048900**	1.74	2.10	0.88	44.4	35.2	74.1	-0.99
**Glyma.18G049600**	2.73	1.85	0.09	34.6	60.3	69.0	-0.97
**Glyma.18G059300**	3.87	3.13	1.35	3.1	33.1	60.0	-1.00
**Glyma.18G061600**	1.58	1.73	0.03	14.3	7.1	59.3	-1.00
**Glyma.18G062000**	3.90	3.74	0.13	34.5	37.9	69.2	-1.00
**Glyma.18G063200**	4.03	0.95	0.01	43.6	75.0	75.0	-1.00
**Glyma.18G065700**	3.19	1.53	0.06	19.2	57.5	76.5	-0.99
**Glyma.18G070800**	3.78	3.44	2.23	8.3	0	57.9	-0.90
**Glyma.18G072800**	2.03	0.37	0.07	32.9	94.7	89.5	-0.99
**Glyma.18G073400**	3.45	3.11	1.65	20.3	37.5	83.3	-1.00
**Glyma.18G073600**	2.08	1.03	6.30E-04	40.1	78.8	92.9	-1.00
**Glyma.18G077000**	4.58	1.63	0.04	21.5	52.8	50.0	-0.99
**Glyma.18G078300**	1.68	1.11	0.14	32.1	60.3	71.4	-0.97
**Glyma.18G081700**	1.41	1.16	0.09	41.0	56.3	73.3	-0.97
**Glyma.18G081800**	1.69	0.98	0.02	13.3	29.5	47.1	-1.00
**Glyma.18G081900**	2.20	1.70	0.53	11.8	33.3	50.0	-0.99
**Glyma.18G082000**	1.68	0.30	0.02	48.9	79.6	81.5	-1.00
**Glyma.18G086000**	2.73	3.17	4.07	52.2	59.4	27.3	-0.90
**Glyma.18G089500**	2.66	2.23	1.37	0	5.3	39.1	-0.95
**Glyma.18G091000**	2.07	2.09	3.41	34.3	55.0	0	-0.93
**Glyma.18G092800**	1.57	0.70	0.05	35.6	51.4	66.7	-0.98
**Glyma.18G095400**	2.96	3.21	1.98	18.2	22.7	51.5	-0.92
**Glyma.18G100500**	5.04	6.38	1.05	18.0	3.2	36.4	-0.98
**Glyma.18G102400**	1.33	1.15	2.93	57.4	61.1	11.1	-1.00
**Glyma.18G102700**	1.50	0	0.06	38.9	74.8	73.1	-1.00

**Glyma.18G104000**	1.37	0.68	0.01	9.6	23.6	46.2	-0.96
**Glyma.18G106300**	3.15	3.60	0.01	50.0	30.4	85.7	-1.00
**Glyma.18G107500**	1.81	1.52	0.32	0	7.1	31.8	-1.00
**Glyma.18G108700**	1.20	0.13	0	47.3	82.4	93.3	-0.99
**Glyma.18G111200**	5.69	5.67	4.45	53.1	55.6	88.9	-1.00
**Glyma.18G111600**	1.74	0.71	0.82	22.5	72.1	62.5	-0.99
**Glyma.18G111900**	1.57	0.59	0.43	47.1	75.2	82.4	-0.99
**Glyma.18G114900**	5.06	2.64	0.22	0	15.7	30.0	-0.94
**Glyma.18G115400**	1.23	1.39	0.04	7.4	3.7	51.9	-1.00
**Glyma.18G116500**	2.97	5.55	5.97	34.8	18.4	0	-0.96
**Glyma.18G121600**	3.81	3.48	0.38	15.3	24.3	55.6	-1.00
**Glyma.18G124900**	0.26	0.45	3.36	32.9	50.0	5.7	-0.92
**Glyma.18G125600**	3.26	3.06	0.39	14.0	24.0	60.0	-1.00
**Glyma.18G126800**	1.31	0.81	1.50E-05	25.9	32.6	64.5	-0.94
**Glyma.18G130700**	2.18	1.06	0.45	11.4	43.2	64.7	-0.98
**Glyma.18G132500**	1.85	1.16	0.84	21.0	37.1	66.7	-0.90
**Glyma.18G133100**	1.97	0.75	0.40	40.9	90.9	81.8	-0.96
**Glyma.18G137100**	2.64	3.03	4.26	51.4	21.4	0	-0.89
**Glyma.18G139100**	1.81	1.54	0.44	6.3	25.0	47.4	-0.98
**Glyma.18G139400**	5.68	5.22	4.20	0	23.4	68.4	-1.00
**Glyma.18G145400**	2.02	2.08	0.60	6.3	3.1	37.5	-1.00
**Glyma.18G145700**	0.87	1.37	2.85	30.7	14.7	0	-0.91
**Glyma.18G148500**	3.46	2.79	0.53	0	11.5	37.5	-0.99
**Glyma.18G152900**	2.97	1.35	0.18	24.1	61.3	51.7	-0.90
**Glyma.18G156100**	0.74	1.01	0	12.2	17.7	55.6	-0.90
**Glyma.18G158900**	3.49	2.75	0.20	12.9	16.7	54.6	-0.93
**Glyma.18G164800**	3.81	3.57	2.59	15.8	35.5	68.4	-0.99
**Glyma.18G167000**	1.38	0.61	0.24	0	21.7	30.4	-1.00
**Glyma.18G173000**	2.59	3.14	5.56	32.1	50.0	8.8	-0.87
**Glyma.18G174200**	1.09	0.44	0	28.9	58.8	94.1	-0.97
**Glyma.18G179800**	1.92	1.20	0.14	46.7	60.0	80.0	-0.98
**Glyma.18G181300**	2.40	1.78	0.25	20.5	36.4	59.1	-1.00
**Glyma.18G182400**	0.15	0.68	1.15	40.5	16.6	5.9	-0.96
**Glyma.18G184000**	1.87	1.22	0.13	20.5	38.6	54.6	-1.00
**Glyma.18G184900**	4.90	3.98	0.03	27.8	47.1	62.5	-1.00
**Glyma.18G185200**	4.11	3.63	3.07	25.0	53.6	80.8	-1.00
**Glyma.18G187500**	0.39	2.09	3.79	61.3	29.0	16.1	-0.86
**Glyma.18G191600**	3.18	1.35	0.02	2.2	33.1	28.6	-0.95
**Glyma.18G193600**	4.21	3.04	0.34	20.8	50.0	52.2	-0.94
**Glyma.18G193700**	1.21	0.66	0.04	23.1	52.1	80.8	-1.00
**Glyma.18G193900**	2.75	2.30	1.25	32.5	60.5	73.7	-0.95
**Glyma.18G195900**	4.58	2.92	2.00	30.6	54.8	61.3	-1.00
**Glyma.18G196000**	3.83	2.88	1.82	0	38.0	53.3	-1.00

**Glyma.18G200300**	3.82	4.09	1.31	0	0	41.7	-0.98
**Glyma.18G202100**	3.27	2.86	1.05	0	7.7	41.7	-0.99
**Glyma.18G209300**	2.54	3.24	1.03	0	0	34.8	-0.87
**Glyma.18G210900**	6.80	4.23	0.33	20.8	50.0	60.0	-1.00
**Glyma.18G211400**	3.83	2.94	2.47	19.3	32.8	62.5	-0.88
**Glyma.18G212700**	1.12	0.57	0.06	1.6	23.2	33.3	-1.00
**Glyma.18G215800**	3.48	3.40	0.64	39.1	62.5	80.0	-0.85
**Glyma.18G216400**	1.67	1.51	0.25	12.1	26.7	72.7	-1.00
**Glyma.18G221200**	1.41	0.84	0.25	2.8	26.4	38.9	-1.00
**Glyma.18G225700**	1.13	1.82	0.03	28.8	7.7	38.5	-0.99
**Glyma.18G232500**	3.52	0.28	0.07	42.2	60.3	76.0	-0.89
**Glyma.18G234700**	2.29	1.75	1.16	9.5	30.3	50.0	-1.00
**Glyma.18G236300**	1.31	0.45	0.02	13.8	54.2	75.0	-0.99
**Glyma.18G243200**	3.66	3.38	0.88	24.7	14.3	56.3	-0.91
**Glyma.18G246300**	4.59	3.98	3.49	17.5	63.9	57.1	-0.88
**Glyma.18G255600**	2.02	1.81	0.80	15.8	57.7	100.0	-0.96
**Glyma.18G258000**	7.22	6.12	0.92	10.0	22.8	54.6	-0.96
**Glyma.18G258600**	2.14	0.92	0.10	40.3	67.4	72.7	-1.00
**Glyma.18G263900**	4.42	3.94	1.02	0	0	32.3	-0.95
**Glyma.18G264000**	1.95	2.16	0.54	0	5.6	45.5	-0.96
**Glyma.18G268400**	3.89	3.27	2.73	37.0	73.8	69.0	-0.88
**Glyma.18G271000**	2.00	0.47	0.01	34.0	71.7	79.2	-1.00
**Glyma.18G273100**	2.10	2.07	1.00	44.0	60.0	76.0	-0.88
**Glyma.18G280900**	2.38	1.63	0.04	15.5	15.6	57.9	-0.87
**Glyma.18G284500**	2.77	2.01	0.28	0	0	68.4	-0.87
**Glyma.18G284600**	2.89	2.42	1.61	0	0	37.5	-0.88
**Glyma.18G286200**	3.58	3.51	2.12	0	5.6	57.1	-1.00
**Glyma.18G287300**	3.03	2.81	1.02	54.2	45.8	94.4	-0.94
**Glyma.18G297400**	3.81	3.33	2.14	6.3	19.2	38.5	-1.00
**Glyma.18G297900**	2.82	2.76	1.01	12.5	18.8	52.2	-1.00
**Glyma.18G302100**	2.40	1.92	0.88	7.5	27.5	55.0	-1.00
**Glyma.19G005900**	1.36	0.95	9.72E-06	42.3	56.9	91.7	-0.99
**Glyma.19G007800**	2.80	2.60	1.51	0	20.8	51.7	-0.98
**Glyma.19G017000**	2.40	1.45	0.09	25.0	61.2	85.7	-1.00
**Glyma.19G018100**	3.14	3.37	1.82	0	0	50.0	-0.98
**Glyma.19G019000**	2.15	1.84	0.93	6.4	35.4	47.1	-0.91
**Glyma.19G020600**	0.02	0.19	3.54	47.9	47.9	15.4	-1.00
**Glyma.19G021500**	2.90	1.46	0.15	7.7	15.4	38.5	-0.85
**Glyma.19G022500**	3.72	2.15	0.26	16.0	54.0	69.6	-1.00
**Glyma.19G025300**	3.27	2.71	0.84	40.0	60.0	93.3	-1.00
**Glyma.19G026100**	2.32	0.20	0.32	17.2	74.0	80.0	-0.99
**Glyma.19G026600**	2.43	1.68	0.12	2.4	21.4	47.6	-0.99
**Glyma.19G027100**	3.86	3.67	0.82	55.6	68.6	94.7	-0.98

**Glyma.19G027700**	2.29	1.93	0.20	1.7	19.6	40.0	-0.98
**Glyma.19G027800**	2.64	2.62	0.68	17.0	10.0	41.7	-0.97
**Glyma.19G028600**	1.29	0.36	0.03	38.1	90.9	95.5	-0.99
**Glyma.19G030800**	3.53	2.40	0.03	2.3	32.6	60.9	-1.00
**Glyma.19G031100**	1.60	1.05	0.35	10.0	54.3	82.8	-1.00
**Glyma.19G031200**	1.22	0.45	0.07	2.3	29.6	35.0	-1.00
**Glyma.19G034700**	1.93	3.04	3.98	63.1	50.0	24.2	-1.00
**Glyma.19G035800**	1.28	0.53	0.01	11.4	47.7	59.1	-1.00
**Glyma.19G036100**	2.79	1.97	0.70	5.2	24.4	47.4	-0.99
**Glyma.19G036400**	1.00	1.04	1.63E-06	0	26.3	69.6	-0.91
**Glyma.19G039100**	1.02	0.22	2.09E-06	38.0	90.0	96.0	-1.00
**Glyma.19G040200**	2.04	1.95	3.39	33.8	32.9	0	-1.00
**Glyma.19G041200**	4.11	3.74	2.00	15.8	18.4	47.4	-0.98
**Glyma.19G043200**	2.91	3.04	2.00	32.8	37.5	68.8	-0.96
**Glyma.19G044200**	1.91	0.48	0.95	16.7	48.5	50.0	-0.97
**Glyma.19G044900**	1.24	0.88	0.03	9.5	27.3	57.6	-1.00
**Glyma.19G045800**	1.18	0.62	0	33.4	56.3	68.8	-1.00
**Glyma.19G046000**	0.93	1.09	0.02	1.7	10.0	33.3	-0.90
**Glyma.19G046600**	4.31	1.76	0.26	16.7	50.0	91.7	-0.89
**Glyma.19G049200**	6.07	5.27	4.11	2.1	20.8	33.3	-1.00
**Glyma.19G055400**	1.95	0.28	3.81E-05	0	41.6	33.3	-0.97
**Glyma.19G060000**	0.97	1.00	0	2.3	13.6	36.4	-0.93
**Glyma.19G061200**	1.79	0.87	2.76	51.8	64.4	29.6	-1.00
**Glyma.19G061900**	1.03	1.10	0.03	0	0	30.0	-1.00
**Glyma.19G062700**	0.25	0.08	1.85	20.0	42.9	0	-0.87
**Glyma.19G063400**	8.30	8.16	4.15	13.5	11.8	42.3	-0.99
**Glyma.19G063800**	2.20	1.11	0.06	6.3	27.7	38.1	-1.00
**Glyma.19G067800**	5.82	5.33	2.90	3.8	19.0	41.4	-1.00
**Glyma.19G068300**	5.30	5.17	3.77	7.1	11.9	52.4	-1.00
**Glyma.19G069200**	2.15	2.10	0.90	0	0	33.3	-1.00
**Glyma.19G071300**	3.35	3.24	5.06	51.4	34.3	14.3	-0.87
**Glyma.19G072100**	4.61	3.73	3.46	6.1	37.2	56.1	-0.97
**Glyma.19G075100**	2.95	1.90	2.03	12.5	50.0	44.4	-1.00
**Glyma.19G076300**	1.50	0.80	0.42	6.8	31.1	54.6	-0.97
**Glyma.19G082000**	1.27	0.26	0.02	9.3	61.4	81.8	-0.99
**Glyma.19G084900**	2.09	1.03	1.53	24.0	61.5	57.1	-0.96
**Glyma.19G085500**	1.15	1.20	0.10	20.2	25.0	73.3	-0.99
**Glyma.19G086700**	1.47	0.38	0.03	33.8	89.3	100.0	-1.00
**Glyma.19G087900**	1.61	1.67	0.03	11.5	18.4	57.9	-0.98
**Glyma.19G089100**	2.44	1.92	0.83	38.1	67.9	100.0	-1.00
**Glyma.19G098300**	3.03	3.35	1.09	15.4	17.3	46.2	-0.96
**Glyma.19G099400**	0.95	1.15	3.86	80.0	62.1	12.0	-0.97
**Glyma.19G100000**	1.63	2.80	7.40	47.1	60.7	7.7	-0.96

**Glyma.19G101600**	2.82	2.27	1.73	45.8	66.7	80.6	-1.00
**Glyma.19G105100**	2.63	1.10	0.05	2.9	45.0	47.8	-0.99
**Glyma.19G105400**	3.58	3.10	0.33	3.8	14.7	37.5	-1.00
**Glyma.19G105500**	1.42	1.05	2.57E-05	21.6	32.1	67.6	-0.99
**Glyma.19G108300**	2.03	1.84	0.65	10.3	26.5	47.1	-0.96
**Glyma.19G109500**	1.78	1.30	0.77	6.7	35.2	53.1	-1.00
**Glyma.19G112500**	1.98	2.02	3.42	25.2	32.7	0	-0.97
**Glyma.19G116400**	1.66	2.15	3.58	40.0	51.5	3.2	-0.93
**Glyma.19G117000**	2.36	1.71	0.13	26.5	41.2	58.8	-1.00
**Glyma.19G117800**	1.57	3.39	5.76	53.7	58.8	27.3	-0.96
**Glyma.19G120000**	3.73	3.78	2.18	13.0	23.9	60.9	-0.97
**Glyma.19G120400**	6.52	5.13	2.04	22.0	48.7	69.2	-1.00
**Glyma.19G123900**	3.31	2.87	2.14	0	0	31.6	-0.88
**Glyma.19G125700**	2.54	2.96	0.78	0	0	35.3	-0.95
**Glyma.19G126700**	1.99	0.80	0.28	38.1	70.0	96.2	-0.96
**Glyma.19G127400**	2.62	2.03	0.02	30.7	69.4	83.3	-0.93
**Glyma.19G128800**	1.44	0.71	0.34	20.1	70.6	81.3	-1.00
**Glyma.19G130100**	3.40	2.60	0.38	11.6	14.8	63.0	-0.90
**Glyma.19G131500**	5.10	5.93	4.42	50.0	40.4	76.9	-0.89
**Glyma.19G131800**	1.14	0.44	0.14	10.9	51.4	59.4	-1.00
**Glyma.19G132700**	1.16	0.99	0.02	0	0	43.8	-0.98
**Glyma.19G132800**	3.77	2.73	1.48	25.0	69.2	66.7	-0.92
**Glyma.19G133000**	1.92	2.40	0.16	10.4	12.7	40.7	-0.91
**Glyma.19G139300**	3.23	1.80	0.22	38.6	63.6	77.8	-1.00
**Glyma.19G139800**	3.06	2.92	1.22	47.5	74.5	94.1	-0.88
**Glyma.19G140100**	2.69	2.16	0.13	30.0	75.0	84.2	-0.87
**Glyma.19G140500**	1.26	0.29	0	21.2	63.5	69.2	-1.00
**Glyma.19G144500**	1.90	0.57	0	28.4	72.9	60.0	-0.90
**Glyma.19G145800**	2.36	1.21	0.30	8.3	68.4	83.3	-1.00
**Glyma.19G148200**	1.58	0.93	0.30	35.2	75.9	70.4	-0.87
**Glyma.19G152000**	0.77	1.11	2.21	74.6	86.7	46.7	-0.90
**Glyma.19G156900**	4.40	3.57	2.18	25.6	55.6	77.8	-1.00
**Glyma.19G157000**	2.73	1.68	3.40E-05	23.5	74.2	76.9	-0.94
**Glyma.19G163200**	4.90	4.68	0.57	34.8	49.7	100.0	-1.00
**Glyma.19G172200**	7.35	6.36	6.14	52.4	85.7	95.2	-1.00
**Glyma.19G172400**	5.86	5.68	4.70	47.7	61.4	90.9	-0.99
**Glyma.19G172800**	2.41	1.66	0.35	39.8	57.1	71.4	-1.00
**Glyma.19G176600**	0.91	2.17	1.79	40.0	5.3	0	-0.86
**Glyma.19G186100**	9.82	6.66	5.65	27.2	81.0	100.0	-0.98
**Glyma.19G190800**	5.04	3.81	2.40	2.4	19.1	38.5	-0.97
**Glyma.19G190900**	7.33	6.14	4.68	38.1	61.4	72.7	-1.00
**Glyma.19G201000**	1.64	1.39	0.11	28.6	47.1	85.7	-1.00
**Glyma.19G201500**	3.59	3.30	0.52	0	0	46.7	-0.98

**Glyma.19G204400**	4.06	3.66	1.08	5.9	27.9	54.6	-0.98
**Glyma.19G210700**	1.30	1.22	8.99E-04	8.9	13.0	47.8	-1.00
**Glyma.19G210900**	3.94	3.97	2.03	17.1	14.8	48.4	-1.00
**Glyma.19G211200**	5.05	4.21	3.11	13.9	79.6	100.0	-0.98
**Glyma.19G211700**	2.57	1.81	0.05	34.8	57.7	75.0	-1.00
**Glyma.19G214900**	1.10	0.20	0	12.9	45.2	45.2	-0.99
**Glyma.19G215100**	2.75	1.41	1.11	16.1	43.1	71.0	-0.91
**Glyma.19G217200**	2.96	3.19	4.47	28.2	32.5	0	-0.98
**Glyma.19G221600**	3.46	1.63	0.02	20.5	81.7	91.7	-1.00
**Glyma.19G221900**	0.85	1.15	0.01	13.5	8.9	53.6	-0.97
**Glyma.19G222400**	3.00	2.38	1.05	32.2	79.0	100.0	-0.97
**Glyma.19G223200**	2.81	1.95	0.31	24.5	74.3	81.3	-0.94
**Glyma.19G223400**	2.48	2.17	1.25	3.3	21.7	50.0	-1.00
**Glyma.19G223600**	4.08	4.08	6.40	81.5	83.3	51.9	-1.00
**Glyma.19G224000**	1.90	2.57	3.53	40.0	41.3	10.0	-0.95
**Glyma.19G228000**	3.40	4.26	2.85	37.5	21.0	72.7	-0.90
**Glyma.19G236400**	3.14	1.98	1.26	33.3	75.4	87.9	-1.00
**Glyma.19G236500**	5.83	4.75	2.82	12.9	47.9	70.8	-1.00
**Glyma.19G236700**	0.30	0.67	6.24	66.7	46.9	22.2	-0.90
**Glyma.19G240100**	6.64	5.60	3.56	7.9	44.7	64.7	-1.00
**Glyma.19G240800**	6.39	4.05	0.59	2.2	13.5	36.8	-0.85
**Glyma.19G240900**	2.63	1.59	0.24	0	13.2	62.5	-0.89
**Glyma.19G244800**	2.20	0.25	0.71	15.0	77.5	95.0	-0.95
**Glyma.19G246800**	3.01	3.09	1.73	36.1	31.3	63.6	-1.00
**Glyma.19G254200**	1.20	0.65	0.15	0	14.3	71.4	-0.89
**Glyma.19G255000**	0.51	0.75	4.10	79.4	64.7	29.4	-0.96
**Glyma.19G258600**	2.10	1.62	0.79	0	0	46.2	-0.88
**Glyma.19G258900**	2.65	2.94	0.09	32.4	12.5	70.6	-0.99
**Glyma.19G259400**	1.34	0.10	0.02	7.7	40.6	31.8	-0.96
**Glyma.19G259900**	2.62	1.09	1.36	13.6	54.1	45.5	-0.99
**Glyma.19G261600**	2.41	0.76	0	16.1	49.3	64.3	-0.99
**Glyma.20G006600**	1.52	0.28	0.02	60.2	91.6	96.2	-1.00
**Glyma.20G007800**	3.73	3.41	0.22	28.2	50.1	67.5	-0.93
**Glyma.20G010600**	1.15	0.48	0.13	42.1	65.7	79.6	-0.99
**Glyma.20G018600**	0.15	0.12	1.37	15.6	33.3	0	-0.86
**Glyma.20G019300**	3.77	3.48	2.71	41.7	54.2	87.5	-1.00
**Glyma.20G019700**	4.40	3.29	1.70E-05	21.4	71.0	78.6	-0.95
**Glyma.20G021700**	3.47	3.47	2.40	37.5	52.5	75.0	-0.92
**Glyma.20G022900**	2.51	2.14	0.88	12.5	14.7	64.0	-0.96
**Glyma.20G025500**	4.67	4.38	6.16	97.1	88.6	28.6	-0.98
**Glyma.20G025700**	1.87	0.47	0.02	23.0	82.6	87.0	-1.00
**Glyma.20G030100**	1.50	0.79	0.08	26.6	58.0	62.5	-0.96
**Glyma.20G031400**	4.92	4.15	3.58	25.6	45.0	69.0	-0.96

**Glyma.20G045500**	6.24	5.66	4.70	0	13.6	34.8	-0.99
**Glyma.20G051900**	3.69	1.29	0.02	0	39.8	75.0	-0.93
**Glyma.20G053200**	3.63	3.27	2.05	5.9	18.2	40.9	-1.00
**Glyma.20G058100**	4.79	3.27	0.93	24.8	61.6	71.4	-1.00
**Glyma.20G059200**	2.76	1.36	0.06	16.5	36.3	50.0	-0.99
**Glyma.20G064400**	2.20	1.46	1.00	16.1	62.5	86.2	-1.00
**Glyma.20G065900**	4.07	3.34	4.90	56.1	69.9	20.0	-1.00
**Glyma.20G066500**	1.76	0.62	0.12	19.5	61.5	82.4	-0.99
**Glyma.20G066600**	4.50	4.44	5.71	28.6	50.6	0	-0.91
**Glyma.20G070200**	2.09	2.28	5.51	21.9	35.9	3.1	-0.90
**Glyma.20G076200**	1.22	0.12	0	12.0	74.0	75.0	-1.00
**Glyma.20G081000**	4.88	3.91	0.50	5.3	30.3	50.0	-1.00
**Glyma.20G082000**	3.78	3.95	2.63	16.5	8.0	40.7	-1.00
**Glyma.20G082800**	4.39	2.90	1.55	13.6	27.3	59.4	-0.87
**Glyma.20G083000**	2.37	2.36	0.24	7.4	16.7	59.3	-0.99
**Glyma.20G083500**	1.05	1.79	4.90	50.2	66.6	31.3	-0.87
**Glyma.20G087600**	2.05	1.94	0.65	15.6	23.4	46.9	-0.99
**Glyma.20G090400**	1.56	0.31	0.09	15.6	92.1	88.2	-0.99
**Glyma.20G091400**	2.56	1.44	0.62	0	59.9	80.8	-1.00
**Glyma.20G092000**	2.59	1.51	0.38	45.9	81.2	92.3	-1.00
**Glyma.20G092400**	3.78	3.29	1.85	3.3	22.4	52.9	-1.00
**Glyma.20G095500**	4.35	4.04	1.11	34.1	38.6	68.2	-1.00
**Glyma.20G095900**	3.28	1.48	0.03	30.8	61.5	61.5	-0.98
**Glyma.20G098400**	2.46	1.41	0.29	40.6	83.6	91.3	-0.98
**Glyma.20G100600**	4.24	2.49	0.85	7.1	41.2	50.0	-1.00
**Glyma.20G103300**	3.92	3.96	5.65	45.0	50.0	17.7	-0.99
**Glyma.20G104900**	1.19	0.85	2.03	75.0	87.5	50.0	-0.99
**Glyma.20G106500**	4.20	4.23	2.42	17.9	16.7	50.0	-1.00
**Glyma.20G108800**	3.04	2.52	1.57	2.4	0	45.0	-0.86
**Glyma.20G111200**	4.89	4.69	3.76	11.5	26.0	68.8	-1.00
**Glyma.20G112600**	2.33	1.59	0.49	17.4	43.1	79.2	-0.99
**Glyma.20G112800**	4.49	4.44	2.77	21.4	21.7	53.6	-1.00
**Glyma.20G119100**	1.88	1.00	0.05	0	16.7	40.0	-0.97
**Glyma.20G120700**	3.36	2.99	0.50	22.2	27.4	57.7	-0.99
**Glyma.20G123600**	6.61	6.05	4.67	8.8	18.2	46.7	-0.98
**Glyma.20G125200**	3.37	2.14	1.39	45.2	73.0	85.0	-1.00
**Glyma.20G126300**	3.39	3.31	0.26	14.3	9.5	47.1	-0.99
**Glyma.20G127900**	1.33	0.44	0.05	13.2	36.9	54.6	-0.97
**Glyma.20G136000**	7.60	6.86	1.70	28.8	83.8	91.7	-0.87
**Glyma.20G145800**	2.02	0.34	0.02	42.5	74.2	81.8	-0.99
**Glyma.20G146900**	1.58	1.78	0.48	0	0	33.3	-0.98
**Glyma.20G151400**	2.46	1.39	1.05	29.2	76.7	70.8	-0.97
**Glyma.20G152500**	0.08	0.33	1.87	34.4	50.0	0	-0.93

**Glyma.20G153200**	4.18	3.42	0.49	0	0	70.0	-0.90
**Glyma.20G153600**	1.47	0.80	0.02	38.3	56.7	73.3	-1.00
**Glyma.20G155300**	4.94E-03	0.73	4.27	36.2	33.6	0	-1.00
**Glyma.20G162000**	2.42	2.28	0.77	0	0	30.0	-0.99
**Glyma.20G162200**	2.90	1.63	1.52	4.7	34.3	35.3	-1.00
**Glyma.20G164700**	9.83	8.48	2.87	10.6	40.0	60.0	-1.00
**Glyma.20G166400**	3.20	1.90	0.03	0	36.8	52.9	-1.00
**Glyma.20G167300**	2.11	2.08	1.07	0	0	50.0	-1.00
**Glyma.20G168200**	4.62	3.27	0.10	28.6	51.8	85.7	-0.97
**Glyma.20G169200**	2.13	1.84	0.57	40.7	44.4	74.1	-0.99
**Glyma.20G170300**	1.26	1.85	0.04	0	0	31.3	-0.88
**Glyma.20G170500**	1.06	0.82	1.94E-04	0	0	33.3	-0.96
**Glyma.20G172900**	2.73	1.70	0.07	7.5	27.3	45.8	-0.99
**Glyma.20G175300**	4.64	3.99	5.16	50.0	75.0	28.6	-1.00
**Glyma.20G176900**	3.55	3.33	2.51	15.9	42.0	84.0	-0.99
**Glyma.20G177700**	6.85	5.94	5.47	6.0	23.9	37.9	-0.98
**Glyma.20G178300**	1.60	0.99	0.14	21.4	44.2	64.3	-1.00
**Glyma.20G183600**	1.80	1.02	0	20.3	42.2	53.1	-1.00
**Glyma.20G189600**	5.12	3.13	0.28	42.7	77.3	72.2	-0.94
**Glyma.20G189900**	2.85	3.19	1.00	5.9	26.8	92.3	-0.88
**Glyma.20G191600**	3.67	2.14	0.43	2.4	36.7	31.6	-0.92
**Glyma.20G193800**	1.95	1.08	0.17	2.5	60.7	60.7	-0.93
**Glyma.20G195600**	2.01	1.46	0.59	7.8	46.0	69.2	-0.99
**Glyma.20G202100**	1.88	2.16	3.97	48.8	52.7	17.7	-0.99
**Glyma.20G202700**	2.90	2.24	0.31	0	36.5	63.6	-0.99
**Glyma.20G207500**	6.40	5.31	4.13	36.7	52.1	87.5	-0.92
**Glyma.20G212000**	1.79	1.25	6.88E-08	40.0	55.0	85.0	-0.99
**Glyma.20G217500**	3.80	3.57	2.28	10.6	15.3	58.8	-0.99
**Glyma.20G224400**	3.65	3.20	5.71	59.5	71.1	39.1	-0.96
**Glyma.20G226500**	5.31	5.05	3.82	5.3	23.7	66.7	-1.00
**Glyma.20G233900**	3.25	3.11	0.03	0	5.0	38.5	-1.00
**Glyma.20G242200**	2.17	2.26	4.74	51.9	57.4	25.9	-0.98
**Glyma.20G247000**	2.67	2.34	1.29	9.6	19.2	50.0	-0.99

Table S5. The Gene Ontology (GO) analysis of the 733 genes with CHH DMRs and decreased expression during late seed maturation

GO ID	Genome GO Count	Observed no. of genes	Expected no. of genes	Presentation	Corrected Probability	GO Description
**GO:0070300**	17	5	0.311034761519806	Overrepresented	0.001024058	pattern specification process
**GO:0043481**	310	28	6.05496916471261	Overrepresented	0.026264565	anthocyanin accumulation in tissues in response to UV

Table S6. Seventy-seven genes with seed-specific CHH DMRs

Genes	S2 FPKM	S6 FPKM	S8 FPKM	Cluster Group	Image Order in Fig S3	Putative Functions or Homologs
**Glyma.04G110000**	43.57	20.22	0.10	1	1	Pyridoxal phosphatase / Vitamin B6-phosphate phosphatase
**Glyma.13G317000**	21.39	29.44	0.10	1	2	PROTEIN ABSCISIC ACID-INSENSITIVE 5
**Glyma.09G260900**	35.85	16.49	0.15	1	3	Cytochrome P450
**Glyma.03G112100**	19.64	24.82	0.13	1	4	ETHYLENE-RESPONSIVE TRANSCRIPTION FACTOR 13
**Glyma.11G227600**	19.97	16.30	0.02	1	5	Transferase/Catalysis of the transfer of an acyl group
**Glyma.04G241800**	20.96	21.32	0.23	1	6	Unknown
**Glyma.01G046300**	20.78	13.90	0.03	1	7	GLUCOSYL/GLUCURONOSYL TRANSFERASES
**Glyma.08G261900**	20.10	17.72	0.15	1	8	LOB DOMAIN-CONTAINING PROTEIN 42
**Glyma.03G065900**	22.76	9.59	0.02	1	9	Pyridoxal phosphatase / Vitamin B6-phosphate phosphatase
**Glyma.19G120400**	42.49	26.32	4.17	1	10	2-isopropylmalate synthase / Isopropylmalate synthetase // Homocitrate synthase
**Glyma.10G030400**	15.00	10.21	0.01	1	11	Unknown
**Glyma.08G284500**	19.90	21.18	1.26	1	12	PROTEIN TRICHOME BIREFRINGENCE-LIKE 2
**Glyma.09G047500**	14.85	17.73	0.62	1	13	PURINE PERMEASE 4-RELATED
**Glyma.07G117500**	8.08	6.99	2.43E-09	2	14	Transcription factor HAND2/Transcription factor TAL1/TAL2/LYL1
**Glyma.15G208600**	5.86	6.29	3.40E-03	2	15	AUXIN EFFLUX CARRIER COMPONENT 2
**Glyma.09G110900**	5.50	4.98	1.54E-03	2	16	Unknown
**Glyma.20G172900**	7.47	2.90	4.71E-03	2	17	Unknown
**Glyma.06G235900**	6.17	1.99	2.29E-03	2	18	AMINOTRANSFERASE TAT2-RELATED
**Glyma.19G240900**	6.89	2.52	0.06	2	19	Unknown
**Glyma.18G209300**	6.43	10.53	1.06	2	20	Unknown
**Glyma.17G156000**	2.99	3.28	5.38E-05	2	21	TRANSCRIPTION FACTOR BHLH18-RELATED
**Glyma.15G155600**	4.31	1.48	9.26E-04	2	22	LRR RECEPTOR-LIKE SERINE/THREONINE-PROTEIN KINASE GSO1-RELATED
**Glyma.10G119900**	2.70	1.84	1.46E-03	2	23	Glycerol-3-phosphate 1-O-acyltransferase
**Glyma.18G145400**	4.06	4.34	0.36	2	24	ZF-HD protein dimerisation region (ZF-HD_dimer)
**Glyma.02G126200**	2.16	1.78	5.63E-04	2	25	PHYTOSULFOKINES 2-RELATED
**Glyma.13G289000**	3.20	0.88	0	2	26	GLUCOSYL/GLUCURONOSYL TRANSFERASES

**Glyma.07G268100**	2.56	1.11	1.28E-10	2	27	NUCLEAR TRANSCRIPTION FACTOR Y SUBUNIT B-6-RELATED
**Glyma.14G130000**	2.13	2.21	0.03	2	28	MOLYBDATE TRANSPORTER 1
**Glyma.02G287300**	2.86	0.79	4.71E-05	2	29	Cytochrome P450 CYP4/CYP19/CYP26 subfamilies
**Glyma.11G100100**	2.88	2.79	0.27	2	30	CYTOCHROME P450 86B1-RELATED
**Glyma.01G144900**	14.79	11.31	6.18	2	31	GROWTH-REGULATING FACTOR 9
**Glyma.14G146400**	1.28	1.24	4.11E-03	2	32	EXOSTOSIN FAMILY PROTEIN
**Glyma.19G221900**	0.72	1.32	1.00E-04	2	33	AUXIN-RESPONSIVE PROTEIN IAA20-RELATED
**Glyma.19G060000**	0.94	1.00	0	2	34	SERINE/THREONINE-PROTEIN KINASE WNK11-RELATED
**Glyma.08G164700**	2.07	1.26	0.18	2	35	METAL TOLERANCE PROTEIN 10-RELATED
**Glyma.17G139500**	8.38	0.42	3.14E-04	3	36	BIFUNCTIONAL INHIBITOR/LIPID-TRANSFER PROTEIN/SEED STORAGE 2S ALBUMIN SUPERFAMILY PROTEIN
**Glyma.04G241600**	6.25	0.38	0	3	37	Unknown
**Glyma.15G262400**	4.02	0.72	3.39E-03	3	38	METAL TOLERANCE PROTEIN 10-RELATED
**Glyma.16G060300**	6.39	0.05	5.89E-04	3	39	Unknown
**Glyma.13G039600**	5.01	0.03	0	3	40	GIBBERELLIN-REGULATED PROTEIN 12-RELATED
**Glyma.16G052100**	5.76	0.01	3.51E-03	3	41	Unknown
**Glyma.07G148700**	2.92	0.22	1.69E-12	3	42	Predicted Yippee-type zinc-binding protein, involved in DNA-binding
**Glyma.20G066500**	3.10	0.38	0.01	3	43	RAG1-ACTIVATING PROTEIN 1
**Glyma.07G227600**	3.81	0.04	2.49E-03	3	44	involved in N-terminal protein myristoylation, and regulation of anion channel activity
**Glyma.08G220700**	2.37	0.25	0	3	45	cyclin-dependent protein kinase holoenzyme complex
**Glyma.03G154300**	4.18	0.01	3.81E-03	3	46	Unknown
**Glyma.11G173200**	3.40	0.01	0	3	47	Unknown
**Glyma.19G026100**	5.38	0.04	0.10	3	48	serine/threonine protein kinase
**Glyma.02G099100**	3.30	0.25	0.05	3	49	C2H2-like zinc finger protein
**Glyma.13G201600**	2.03	0.24	4.68E-04	3	50	Unknown
**Glyma.18G236300**	1.71	0.20	4.29E-04	3	51	Unknown
**Glyma.13G028500**	1.21	0.21	4.47E-05	3	52	serine-type carboxypeptidase
**Glyma.19G140500**	1.59	0.08	0	3	53	Unknown
**Glyma.07G124600**	2.53	0.10	0.04	3	54	MATE efflux family protein
**Glyma.15G099300**	2.28	7.44E-08	4.04E-12	3	55	1-deoxy-D-xylulose-5-phosphate synthase
**Glyma.16G090800**	1.95	0	0	3	56	Unknown
**Glyma.12G015500**	1.22	0.08	2.51E-07	3	57	Zinc-binding alcohol dehydrogenase family protein
**Glyma.08G353300**	1.80	1.32E-03	2.84E-03	3	58	Plant self-incompatibility protein S1 family
**Glyma.01G089800**	1.24	0.02	0	3	59	GAMMA-BUTYROBETAINE HYDROXYLASE-RELATED protein
**Glyma.13G229800**	1.05	0.03	0	3	60	GDSL/SGNH-like Acyl-Esterase family
**Glyma.16G075100**	1.15	0	7.73E-04	3	61	Unknown
**Glyma.05G126800**	1.85E-03	0.53	4.05	4	62	Stress up-regulated Nod 19 (SURNod19)
**Glyma.20G152500**	0.01	0.11	3.49	4	63	Very-long-chain 3-oxoacyl-CoA synthase
**Glyma.19G062700**	0.06	0.01	3.42	4	64	Unknown
**Glyma.12G129900**	1.98E-03	1.07E-04	5.97	4	65	Unknown
**Glyma.10G072600**	0	2.34E-12	5.98	4	66	L-ascorbate peroxidase

**Glyma.09G145900**	4.03	1.18	16.39	4	67	Unknown
**Glyma.06G090900**	0.05	0.41	10.71	4	68	predicted CAMP-RESPONSE ELEMENT BINDING PROTEIN
**Glyma.16G145600**	1.96E-04	3.17E-04	18.50	4	69	Seven transmembrane MLO family protein
**Glyma.04G139800**	45.77	7.34	0.06	5	70	Disease resistance-responsive (dirigent-like protein) family protein, NUCLEOPORIN-RELATED protein
**Glyma.14G033000**	43.24	2.44	0.32	5	71	Unknown
**Glyma.10G120700**	16.99	2.92	1.58E-03	5	72	Granulin repeat cysteine protease family protein
**Glyma.09G090600**	13.48	0.22	4.03E-03	5	73	Unknown
**Glyma.13G260800**	11.96	0.25	0	5	74	Unknown
**Glyma.08G234100**	13.69	0.26	0.02	5	75	S locus-related glycoprotein 1 binding pollen coat protei (SLR1-BP)
**Glyma.18G232500**	12.38	0.08	0.01	5	76	Plant self-incompatibility protein S1 family
**Glyma.02G084800**	10.93	0.07	2.39E-03	5	77	Unknown

Table S7. Over-representative DMRs in Leaves

GO_id	Genome_GO_count	Expressed_GO	Expected_expression	Status	Corrected_P	GO_desc
**GO:0006633**	237	34	17.67480947	Overrepresented	0.079638701	fatty acid biosynthetic process
**GO:0040007**	201	30	14.99002829	Overrepresented	0.10214142	growth
**GO:0007018**	184	28	13.72221495	Overrepresented	0.147384912	microtubule-based movement
**GO:0008361**	144	23	10.73912474	Overrepresented	0.266215003	regulation of cell size
**GO:0010015**	138	22	10.29166121	Overrepresented	0.381653362	root morphogenesis
**GO:0007389**	179	26	13.34932867	Overrepresented	0.466101237	pattern specification process
**GO:0006468**	2386	211	177.9413308	Overrepresented	0.590775577	protein phosphorylation
**GO:0016126**	363	43	27.07154362	Overrepresented	0.858730018	sterol biosynthetic process
**GO:0006084**	178	25	13.27475142	Overrepresented	1.286719337	acetyl-CoA metabolic process
**GO:0000271**	274	34	20.43416791	Overrepresented	1.624823983	polysaccharide biosynthetic process
**GO:0010199**	19	6	1.416967848	Overrepresented	1.957224557	boundary between organs and meristem
**GO:0006098**	421	47	31.39702442	Overrepresented	2.029195841	pentose-phosphate shunt
